# Morphological and phylogenetic analyses in *Auriculariales* (*Basidiomycota*, *Agaricomycetes*) reveal nine new species in *Basidiodendron*

**DOI:** 10.3897/imafungus.17.204252

**Published:** 2026-09-03

**Authors:** Qi Yuan, Shichun You, Subodini Nuwanthika Wijesinghe, Lu Wang, Xiangfu Liu, Masoomeh Ghobad-Nejhad, Changlin Zhao

**Affiliations:** 1 The Key Laboratory of Forest Resources Conservation and Utilization in the Southwest Mountains of China Ministry of Education, Yunnan Provincial Key Laboratory for Conservation and Utilization of In-forest Resource, Southwest Forestry University, Kunming 650224, China The Key Laboratory of Forest Resources Conservation and Utilization in the Southwest Mountains of China Ministry of Education, Yunnan Provincial Key Laboratory for Conservation and Utilization of In-forest Resource, Southwest Forestry University Kunming China; 2 College of Forestry, Southwest Forestry University, Kunming 650224, China College of Forestry, Southwest Forestry University Kunming China; 3 Modern Industry School of Edible Fungi, Southwest Forestry University, Kunming 650224, China Modern Industry School of Edible Fungi, Southwest Forestry University Kunming China; 4 Royal Botanic Gardens Kew, London, TW9 3DS, UK Royal Botanic Gardens Kew London United Kingdom; 5 Department Microbial Drugs (MWIS), Helmholtz-Centre for Infection Research, 38124 Braunschweig, Germany Department Microbial Drugs (MWIS), Helmholtz-Centre for Infection Research Braunschweig Germany

**Keywords:** *
Heterobasidiomycetes
*, novel taxa, systematics, taxonomy, typification, wood fungi

## Abstract

*Auriculariales* is an important order of wood-inhabiting fungi, accommodating a high diversity of taxa with complex evolutionary relationships. Within this order, the generic boundaries and species diversity of *Basidiodendron* have remained incompletely resolved. Morphologically, the genus is characterized by annual, resupinate basidiomata, a monomitic hyphal system with clamped generative hyphae, longitudinally septate four-celled basidia with well-developed or indistinct involucres, and globose to subglobose basidiospores exhibiting smooth, warted, or spiny walls. Based on an integrative approach combining morphological characterization and multigene phylogeny inferred from ITS, nrLSU, *RPB*1, *RPB*2, and *TEF*1 sequence data, nine new species of *Basidiodendron* are formally introduced, namely *B. album***sp. nov**., *B. arachnoideum***sp. nov**., *B. dehongense***sp. nov**., *B. fragilissimum***sp. nov**., *B. odontoideum***sp. nov**., *B. rigidum***sp. nov**., *B. ruiliense***sp. nov**., *B. tenissimum***sp. nov**., and *B. zhaotongense***sp. nov**. Detailed morphological descriptions, illustrations, and phylogenetic evaluations are provided for all newly described taxa. The generated multilocus dataset represents a substantial advance in understanding species diversity and phylogenetic relationships within the genus. This study significantly enriches knowledge of wood-inhabiting fungi and provides a more robust taxonomic framework for future research on *Basidiodendron* and related taxa in *Auriculariales*.

## Introduction

Wood-inhabiting fungi are primary agents of lignocellulose decomposition, driving essential nutrient cycling and global biogeochemical dynamics in terrestrial ecosystems (Larsson 2007; Pawlowski et al. 2012; Dai et al. 2021; Crous et al. 2025, 2026). By efficiently dismantling complex wood polymers, this specialized ecological group facilitates the crucial recycling of carbon, nitrogen, and phosphorus, thereby sustaining forest ecosystem functions (Savchenko et al. 2021; Nagy et al. 2023; Hibbett et al. 2025).

The order *Auriculariales (Agaricomycetes)* encompasses a highly diverse group of wood-inhabiting fungi that exhibit considerable variability in both macroscopic fruiting body structures and basidial morphology (Bromhead 1840; Weiss and Oberwinkler 2001). Historically, the internal familial classification of this order has fluctuated significantly, as early taxonomic frameworks relied heavily on highly variable basidial types, such as transversely, longitudinally, or obliquely septate, as well as entirely non-septate (single-celled) basidia (Talbot 1965; Bandoni 1984; Roberts 1998). The diagnostic significance of these basidial features was subsequently re-evaluated through the integration of ultrastructural characteristics, particularly the spindle pole body and septal pore architecture (Wells 1994; Nitare 2014; Alvarenga et al. 2019). Later, with the introduction of molecular phylogenetics, extensive realignments of its constituent taxa occurred (Weiss and Oberwinkler 2001). To date, only two families within the order *Auriculariales*, viz., *Auriculariaceae* and *Hyaloriaceae*, are widely recognized (He et al. 2024). Despite these molecular advancements, significant portions of the order’s diversity, notably the genus *Basidiodendron* Rick and its allied lineages, remain taxonomically challenging. While recent phylogenetic studies have expanded the knowledge of some related genera (Spirin et al. 2020, 2021, 2025), the generic boundaries and species-level diversity within the *Basidiodendron* complex are still incompletely resolved. The internal topology and species delimitation within this group require further investigation using robust multilocus datasets to accurately evaluate their evolutionary relationships.

Several unknown specimens morphologically conforming to the genus *Basidiodendron* were collected. To accurately determine their taxonomic placements and comprehensively assess the species diversity within the genus, detailed morphological examinations combined with multigene phylogenetic analyses based on ITS+nrLSU+*RPB*1+*RPB*2+*TEF*1 sequences, as well as reduced datasets of ITS+nrLSU+*TEF*1, were conducted. Based on these integrative analyses, nine novel species of *Basidiodendron* are formally introduced. This study aims to provide a highly resolved, genus-level taxonomic framework based on multilocus evidence, significantly enriching the global understanding of species diversity and phylogenetic relationships within the *Basidiodendron* clade in *Auriculariales*.

## Materials and methods

### Sample collection and herbarium specimen preparation

The samples were photographed *in situ*, and detailed collection information was recorded (Dong et al. 2025d). Fresh macroscopic details were also recorded in the field. Photographs were taken with a Jianeng 80D camera (Tokyo, Japan). All photos were focus-stacked and merged using Helicon Focus Pro 7.7.5 software. Macroscopic details were recorded, and the specimens were transported to a field station, where the fresh basidiomata were dried in an electric food dryer at 40 °C (Deng et al. 2026). Once dried, the specimens were sealed in envelopes and zip-lock plastic bags and labeled (Yang et al. 2025). The dried specimens were deposited in the herbarium of Southwest Forestry University (SWFC), Kunming, Yunnan Province, China.

### Morphological studies

The macromorphological descriptions were based on field notes and photographs captured in the field and laboratory. Petersen (1996) was followed for the color terminology. The micromorphological data were obtained from dried specimens observed using a light microscope at 1000× magnification with oil immersion (Wang et al. 2026). Sections were mounted in 5% KOH and 1% phloxine B (C_20_H_2_Br_4_Cl_4_Na_2_O_5_), and Cotton Blue and Melzer’s reagent were used where necessary to observe micromorphology, following the method of Wang et al. (2026). To present the variations in spore sizes, 5% of the measurements at each end of the range were excluded from the main range and shown in parentheses. A minimum of 30 basidiospores from each specimen was measured. Stalks were excluded from basidia measurements, and the hilar appendage was excluded from basidiospore measurements. The MycoBank numbers were registered in the MycoBank database (http://www.mycobank.org).

The following abbreviations are used: CB = Cotton Blue; CB+ = cyanophilous; CB–; IKI = Melzer’s reagent; IKI– = both inamyloid and non dextrinoid; KOH = 5% aqueous potassium hydroxide solution; *L* = mean spore length (arithmetic average for all spores); *W* = mean spore width (arithmetic average for all spores); *n* = *a*/*b* (number of spores [*a*] measured from the given number [*b*] of specimens); *Q* = variation in the *L*/*W* ratios between the specimens studied. If not stated otherwise, the number of measured spores [*a*] was 30.

### Phylogenetic analyses

A CTAB rapid fungal genome extraction kit-DN14 (Aidlab Biotechnologies Co., Ltd., Beijing) was used to obtain genomic DNA from dried specimens according to the manufacturer’s instructions. The gene fragments employed in this study are detailed in Table 1.

**Table 1.** Loci, primers, PCR amplification procedures, and references used in this study.

**Name**	**Abbreviation**	**Primer**	**Direction**	**Sequence (5'-3')**	**PCR amplification procedures**	**References**
Internal transcribed spacer region of the rDNA	ITS	ITS5	Forward	GGAAGTAAAAGTCGTAACAAGG	94 °C 3 min; 35 cycles of 94 °C 40 s, 58 °C 45 s, 72 °C 1 min; 72 °C 10 min.	White et al. (1990)
		ITS4	Reverse	TCCTCCGCTTATTGATATGC		
Nuclear ribosomal large subunit	nrLSU	LR0R	Forward	ACCCGCTGAACTTAAGC	94 °C 1 min; 35 cycles of 94 °C 30 s, 48 °C 1 min, 72 °C 1.5 min; 72 °C 10 min.	Vilgalys and Hester (1990)
		LR7	Reverse	TACTACCACCAAGATCT		
RNA polymerase II largest subunit	*RPB*1	RPB1-Af	Forward	GARTGYCCDGGDCAYTTYGG	94 °C 2 min; nine cycles of 94 °C 40 s; 60 °C 40 s; 72 °C 2 min; 10 cycles of 94 °C 40 s; 94 °C 45 s; 55 °C 1.5 min; 72 °C 5 min; 37 cycles 94 °C 45 s; 72 °C 10 min	Matheny et al. (2002)
		RPB1-Cf	Reverse	CCNGCDATNTCRTTRTCCATRTA		
RNA polymerase II second largest subunit	*RPB*2	bRPB2-6F	Forward	TGGGGYATGGTNTGYCCYGC	95 °C 2.5 min; nine cycles of 95 °C 30 s, 52 °C 1 min; 72 °C 1 min; 40 cycles of 95 °C 30 s; 72 °C 1.5 min; 40 cycles 72 °C 1.5 min; 72 °C 5 min.	Matheny (2005)
		bRPB2-7.1R	Reverse	CCCATRGCTTGYTTRCCCAT		
Translation elongation factor 1-alpha	*TEF*1	EF1-983F	Forward	GCYCCYGGHCAYCGTGAYTTYAT	94 °C 2.5 min; 94 °C 45 s; 60 °C 50 s; 72 °C 2 min; six cycles of 94 °C 45 s; 94 °C 30 s; 55 °C 50 s; 72 °C 1.5 min; 34 cycles 94 °C 30 s; 72 °C 5 min.	Rehner and Samuels (1994)
		EF1-2218R	Reverse	ATGACACCRACRGCRACRGTYTG		

The PCR products were purified and sequenced at Kunming Tsingke Biological Technology Limited Company, Kunming, Yunnan Province, P.R. China. All newly generated sequences were deposited in GenBank (Table 2).

**Table 2.** Names, specimen numbers, references, and corresponding GenBank accession numbers of the taxa used in this study. Newly introduced taxa are indicated in bold black font.

**Species name**	**Specimen no**.	**ITS**	**nrLSU**	***RPB*1**	***RPB*2**	***TEF*1**	**Country**	**References**
*Adustochaete nivea*	RLMA 531	MN165954	MN165989	—	—	—	BRAZIL	Alvarenga et al. (2019)
*Adustochaete rava*	KHL 15526	MK391517	MK391526	—	—	—	BRAZIL	Alvarenga et al. (2019)
*Alloexidiopsis australiensis*	LWZ 20180513-22	OM801933	OM801918	—	—	—	AUSTRALIA	Liu et al. (2022)
*Alloexidiopsis australiensis*	LWZ 20180514-18	OM801934	OM801919	—			CHINA	Liu et al. (2022)
*Alloexidiopsis schistacea*	LWZ 20200819-21a	OM801939	OM801932	—	—	—	CHINA	Liu et al. (2022)
*Amphistereum leveilleanum*	FP-106715	KX262119	KX262168	—	—	—	USA	Malysheva and Spirin (2017)
*Amphistereum schrenkii*	HHB 8476	KX262130	KX262178	—	—	—	USA	Malysheva and Spirin (2017)
*Aporpium caryae*	WD 2207	AB871751	AB871730	—	—	—	JAPAN	Sotome et al. (2014)
*Aporpium hexagonoides*	ML 297	AB871754	—	—	—	—	MALAYSIA	Sotome et al. (2014)
*Auricularia heimuer*	Xiaoheimao	KM396781	KM396834	—	KY419035	KY419083	CHINA	Wang et al. (2023)
*Auricularia mesenterica*	Kytovuori-89-333	KP729284	KP729302	—	KP729321	—	ESTONIA	Wu et al. (2021)
***Basidiodendron album***	**CLZhao 33842**	**PV577724**	**PX724331**	—	**PX739860**	—	**CHINA**	**Present study**
***Basidiodendron album***	**CLZhao 50615**	**PX620086**	**PZ403663**	—	—	—	**CHINA**	**Present study**
*Basidiodendron alni*	HK 26050	MT040878	—	—	—	MT055847	RUSSIA	Spirin et al. (2020)
*Basidiodendron alni*	VS 10913	MT040869	—	—	—	—	RUSSIA	Spirin et al. (2020)
***Basidiodendron arachnoideum***	**CLZhao 39268**	**PZ324411**	—	—	—	—	**CHINA**	**Present study**
***Basidiodendron arachnoideum***	**CLZhao 50722**	**PZ324412**	—	—	—	—	**CHINA**	**Present study**
*Basidiodendron caesiocinereum*	VS 13663	MW136105	MW136140	—	—	MW187110	ITALY	Spirin et al. (2021)
*Basidiodendron caesiocinereum*	VS 12515	MW136100	MW136135	—	—	MW187105	NORWAY	Spirin et al. (2021)
*Basidiodendron caesiocinereum*	VS 12465	MW136102	MW136137	—	—	MW187107	NORWAY	Spirin et al. (2021)
*Basidiodendron caesiocinereum*	VS 12511	MW136083	—	—	—	MW187094	NORWAY	Spirin et al. (2021)
*Basidiodendron caesiocinereum*	VS 11115	MW136103	MW136138	—	—	MW187108	NORWAY	Spirin et al. (2021)
*Basidiodendron caucasicum*	HK 22569	MT040877	MT040856	—	—	MT055855	RUSSIA	Spirin et al. (2020)
*Basidiodendron cinerellum*	VS 11188	MW136104	MW136139	—	—	MW187109	NORWAY	Spirin et al. (2021)
*Basidiodendron cinerellum*	VS 13317	MW136097	MW136131	—	—	MW187102	SLOVENIA	Spirin et al. (2021)
*Basidiodendron cinerellum*	VS 13275	MW136093	MW136130	—	—	MW187101	SLOVENIA	Spirin et al. (2021)
*Basidiodendron cinerellum*	VS 13485	MW136088	MW136125	—	—	MW187097	BELGIUM	Spirin et al. (2021)
*Basidiodendron cinereum*	MW 377	—	AF291295	—	—	—	GERMANY	Weiss and Oberwinkler (2001)
***Basidiodendron dehongense***	**CLZhao 42161**	**PX413148**	—	—	—	—	**CHINA**	**Present study**
***Basidiodendron dehongense***	**CLZhao 50617**	**PZ321701**	—	—	—	—	**CHINA**	**Present study**
*Basidiodendron deminutum*	VS 12589	MT040885	MT040865	—	—	MT074092	SLOVENIA	Spirin et al. (2020)
*Basidiodendron eyrei*	ENZ 18-101	MW139274	—	—	—	—	NETHERLANDS	Spirin et al. (2021)
*Basidiodendron eyrei*	ENZ 18-103	MW139275	—	—	—	—	NETHERLANDS	Spirin et al. (2021)
*Basidiodendron eyrei*	ENZ 13-100	MW139273	—	—	—	—	NETHERLANDS	Spirin et al. (2021)
*Basidiodendron eyrei*	VS 10579	MT040866	—	—	—	MT055844	RUSSIA	Spirin et al. (2020)
***Basidiodendron fragilissimum***	**CLZhao 49779**	**PZ324413**	—	—	—	—	**CHINA**	**Present study**
***Basidiodendron fragilissimum***	**CLZhao 49840**	**PZ324414**	—	—	—	—	**CHINA**	**Present study**
*Basidiodendron gelatinosum*	LIP:0001678	MZ336015	MZ336014	—	—	—	FRANCE	—
*Basidiodendron glaucum*	JN 9080	MW259228	—	—	—	—	NORWAY	Spirin et al. (2021)
*Basidiodendron glaucum*	SS 863	MW136078	MW136119	—	—	MW187090	NORWAY	Spirin et al. (2021)
*Basidiodendron glaucum*	JN 9815	MW259227	—	—	—	—	NORWAY	Spirin et al. (2021)
*Basidiodendron glaucum*	JN 9683	MW259233	—	—	—	—	NORWAY	Spirin et al. (2021)
*Basidiodendron globisporum*	VS 7040	MT040872	—	—	—	MT055852	RUSSIA	Spirin et al. (2020)
*Basidiodendron globisporum*	VS 12929	MT040884	MT040864	—	—	—	RUSSIA	Spirin et al. (2020)
*Basidiodendron globisporum*	VS 13450	MT040888	—	—	—	MT055859	SLOVENIA	Spirin et al. (2020)
*Basidiodendron globisporum*	OM 15584	MT040886	—	—	—	—	USA	Spirin et al. (2020)
*Basidiodendron grandinioides*	VS 12437	MT040873	MT040863	—	—	MT055854	NORWAY	Spirin et al. (2020)
*Basidiodendron grandinioides*	AS 11315	MT040887	—	—	—	—	NORWAY	Spirin et al. (2020)
*Basidiodendron groningae*	GVA 20-040	MW139280	—	—	—	—	BELGIUM	Spirin et al. (2021)
*Basidiodendron groningae*	ENZ 18-001	MW139278	MW136483	—	—	—	NETHERLANDS	Spirin et al. (2021)
*Basidiodendron groningae*	ENZ 19-073	MW139276	MW136482	—	—	—	NETHERLANDS	Spirin et al. (2021)
*Basidiodendron groningae*	NS 18-1325	MW139265	—	—	—	—	NETHERLANDS	Spirin et al. (2021)
*Basidiodendron inconspicuum*	VS 8171	MW136098	MW136132	—	—	MW187103	USA	Spirin et al. (2021)
*Basidiodendron iniquum*	KHL 15469	MT040876	—	—	—	—	BRAZIL	Spirin et al. (2020)
*Basidiodendron luteogriseum*	KHL 16022	MT040881	MT040861	—	—	MT055857	BRAZIL	Spirin et al. (2020)
*Basidiodendron mexicanum*	LR 23131	MW136068	—	—	—	—	MEXICO	Spirin et al. (2021)
*Basidiodendron microsporum*	HK 28653	—	MT040859	—	—	MT055856	RUSSIA	Spirin et al. (2020)
***Basidiodendron odontoideum***	**CLZhao 32441**	**PZ324415**	**PZ403664**	—	—	—	**CHINA**	**Present study**
***Basidiodendron odontoideum***	**CLZhao 50721**	**PZ324416**	—	—	—	—	**CHINA**	**Present study**
*Basidiodendron olivaceum*	VS 8969	MT040870	—	—	—	MT055848	CANADA	Spirin et al. (2020)
*Basidiodendron olivaceum*	VS 4304	MT040883	MT040857	—	—	MT055851	RUSSIA	Spirin et al. (2020)
*Basidiodendron olivaceum*	VS 10780	—	MT040858	—	—	MT055849	RUSSIA	Spirin et al. (2020)
*Basidiodendron olivaceum*	VS 11014	MT040867	—	—	—	MT055850	RUSSIA	Spirin et al. (2020)
*Basidiodendron parile*	VS 11099	MT040890	—	—	—	—	NORWAY	Spirin et al. (2020)
*Basidiodendron parile*	VS 11607	MT040889	—	—	—	MT055860	NORWAY	Spirin et al. (2020)
*Basidiodendron pelinum*	KHL 16014	MT040875	MT040862	—	—	MT055858	BRAZIL	Spirin et al. (2020)
*Basidiodendron* sp.	CLZhao 37380	PX700915	PX724327	—	—	—	CHINA	Present study
*Basidiodendron* sp.	CLZhao 39521	PX700916	PX724328	—	—	—	CHINA	Present study
*Basidiodendron* sp.	CLZhao 42319	PX700917	PX724329	—	—	—	CHINA	Present study
*Basidiodendron* sp.	CLZhao 42453	PX700918	PX724330	PZ414686	—	PZ439657	CHINA	Present study
*Basidiodendron remotum*	HK 16308	—	MT040860	—	—	MT055853	RUSSIA	Spirin et al. (2020)
***Basidiodendron rigidum***	**CLZhao 31785**	**PV577720**	**PX724318**	—	—	—	**CHINA**	**Present study**
***Basidiodendron rigidum***	**CLZhao 33334**	**PV577721**	—	—	—	—	**CHINA**	**Present study**
*Basidiodendron rimosum*	160890	—	AF291298	—	—	—	COSTA RICA	Weiss and Oberwinkler (2001)
*Basidiodendron robenae*	OM 16910.2	MW270998	MW271001	—	—	—	USA	Spirin et al. (2021)
*Basidiodendron robenae*	OM 19650	MW270997	MW271000	—	—	—	USA	Spirin et al. (2021)
***Basidiodendron ruiliense***	**CLZhao 36504**	**PX628482**	**PZ403662**	**PZ414682**	—	—	**CHINA**	**Present study**
***Basidiodendron ruiliense***	**CLZhao 41383**	**PX628483**	**PX724314**	**PZ414683**	—	—	**CHINA**	**Present study**
*Basidiodendron salebrosum*	VS 5024	MT040871	—	—	—	—	RUSSIA	Spirin et al. (2020)
*Basidiodendron salebrosum*	VS 10900	MT040891	—	—	—	—	RUSSIA	Spirin et al. (2020)
*Basidiodendron spiculosum*	LR 23324	MW136076	MW136117	—	—	MW187088	MEXICO	Spirin et al. (2021)
*Basidiodendron* sp.	CLZhao 38891	PX700910	PX724315	PZ414684	—	PZ439654	CHINA	Present study
*Basidiodendron* sp.	CLZhao 39103	PX700911	PX724316	PZ414685	—	PZ439655	CHINA	Present study
*Basidiodendron* sp.	CLZhao 39330	PX700912	PX724317	—	—	PZ439656	CHINA	Present study
***Basidiodendron tenissimum***	**CLZhao 30794**	**PV501097**	**PX724310**	—	—	—	**CHINA**	**Present study**
***Basidiodendron tenissimum***	**CLZhao 30844**	**PV501098**	—	—	—	—	**CHINA**	**Present study**
*Basidiodendron trachysporum*	VS 8740	MW136075	MW136116	—	—	MW187087	US	Spirin et al. (2021)
*Basidiodendron trachysporum*	VS 11886	MW152419	MW136128	—	—	MW187099	RUSSIA	Spirin et al. (2021)
*Basidiodendron trachysporum*	VS 12528	MW136067	MW136113	—	—	MW187083	NORWAY	Spirin et al. (2021)
*Basidiodendron trachysporum*	VS 11803	MW136090	MW136127	—	—	MW187098	NORWAY	Spirin et al. (2021)
*Basidiodendron trachysporum*	VS 12548	MW136099	MW136133	—	—	MW187104	SLOVENIA	Spirin et al. (2021)
*Basidiodendron trachysporum*	VS 11801	MW136091	MW136129	—	—	MW187100	NORWAY	Spirin et al. (2021)
*Basidiodendron walleynii*	WR 3081	MW139279	—	—	—	—	Belgium	Spirin et al. (2021)
*Basidiodendron walleynii*	VS 9697	MW136066	MW136112	—	—	—	RUSSIA	Spirin et al. (2021)
*Basidiodendron widdringtoniae*	LR 11307a	MW136073	MW136114	—	—	—	MALAWI	Spirin et al. (2021)
*Basidiodendron yunnanense*	CLZhao 17858	OM677312	—	—	—	—	CHINA	Duan and Zhao (2022)
*Basidiodendron yunnanense*	CLZhao 17805	OM677311	—	—	—	—	CHINA	Duan and Zhao (2022)
***Basidiodendron zhaotongense***	**CLZhao 32305**	**PV539440**	—	—	—	—	**CHINA**	**Present study**
***Basidiodendron zhaotongense***	**CLZhao 32672**	**PV539442**	**PX724308**	—	—	—	**CHINA**	**Present study**
***Basidiodendron zhaotongense***	**CLZhao 33274**	**PV539443**	**PX724309**	—	—	—	**CHINA**	**Present study**
*Eichleriella alliciens*	HHB 7194	KX262120	KX262169	—	—	—	USA	Malysheva and Spirin (2017)
*Eichleriella alpina*	He 20120916-1	MH178245	MH178268	—	—	—	CHINA	Li et al. (2023)
*Eichleriella bactriana*	TAAM 55071	KX262121	KX262170	—	—	—	TURKMENISTAN	Malysheva and Spirin (2017)
*Elmericium alabastrinum*	Spirin 5311	PV241605	PV241619	—	—	—	RUSSIA	Spirin et al. (2025)
*Elmerina brasiliensis*	SP446226	ON373990	ON373987	—	—	—	BRAZIL	Alvarenga et al. (2023)
*Elmerina cladophora*	X1902	MG757509	MG757509	—	—	—	INDONESIA	Malysheva et al. (2018)
*Endoperplexa dartmorica*	VS 11781	MT235621	MT235602	—	—	—	NORWAY	Spirin et al. (2025)
*Exidia glandulosa*	MW 355	AF291273	AF291319	—	—	—	GERMANY	Weiss and Oberwinkler (2001)
*Exidia glandulosa*	Dai 21232	MT663362	MT664781	—	—	—	CHINA	Wu et al. (2020)
*Exidia pithya*	MW 313	AF291275	AF291321	—	—	—	GERMANY	Weiss and Oberwinkler (2001)
*Exidiopsis effusa*	Miettinen 19136	KX262145	KX262193	—	—	—	FINLAND	Tohtirjap et al. (2023)
*Exidiopsis succinea*	Spirin 5836	PV241617	PV241632	—	—	—	RUSSIA	Spirin et al. (2025)
*Gelacantha pura*	LE 254018	MK098882	MK098930	—	—	—	RUSSIA	Spirin et al. (2019a)
*Grammatus labyrinthinus*	Yuan 1600	KM379139	KM379140	—	—	—	CHINA	Alvarenga et al. (2019)
*Grammatus semis*	OM10618	KX262146	KX262194	—	—	—	CHINA	Malysheva and Spirin (2017)
*Guepinia montana*	Yang6743	OR822233	OR803013	—	—	—	CHINA	Cui et al. (2024)
*Helicomyxa everhartioides*	TNM	—	AY640107	—	—	—	CHINA	He et al. (2019)
*Heteroacanthella acanthophysa*	ID 7083-C1	MW586897	—	—	—	—	BELGIUM	—
*Heterochaete andina*	Lagerheim	—	KX262187	—	—	—	ECUADOR	Malysheva and Spirin (2017)
*Heterocorticium bambusicola*	He4604	MH178260	MH178284	—	—	—	CHINA	Li et al. (2023)
*Heterocorticium latisporum*	He20120923-20	MH178261	MH178285	—	—	—	CHINA	Li et al. (2023)
*Heteroradulum adnatum*	LR 23453	KX262116	KX262165	—	—	—	MEXICO	Malysheva and Spirin (2017)
*Heteroradulum australiense*	LWZ 20180515-26	MZ325256	MZ310426	—	—	—	AUSTRALIA	Li et al. (2023)
*Heteroradulum kmetii*	VS 6466	KX262104	KX262152	—	—	—	RUSSIA	Malysheva and Spirin (2017)
*Hyalodon antui*	Niemelä 6389	MG735416	MG735424	—	—	—	CHINA	Spirin et al. (2019b)
*Hyalodon piceicola*	Spirin 11063	MG735415	MG735423	—	—	—	RUSSIA	Spirin et al. (2019b)
*Hyaloria pilacre*	TI 2768	—	AF291338	—	—	—	VENEZUELA	Weiss and Oberwinkler (2001)
*Hydrophana fessula*	Viner 2021/289	PV394849	PV394849	—	—	—	FI	Spirin et al. (2025)
*Hydrophana sphaerospora*	VS 11622	MK098884	MK098932	—	—	—	NORWAY	Spirin et al. (2019a)
*Hydrophana sphaerospora*	VS 11133	MK098883	MK098931	—	—	—	NORWAY	Spirin et al. (2019a)
*Metulochaete sanctae-catharinae*	AM 0678	MK484065	MK480575	—	—	—	RUSSIA	Spirin et al. (2019b)
*Mycostilla chromatica*	Spirin 14839	PV394846	PV394846	—	—	—	SI	Spirin et al. (2025)
*Mycostilla vermiformis*	Spirin 11330	MG735417	MG735425	—	—	—	RUSSIA	Spirin et al. (2019b)
*Myxariellum concinnum*	VS 8393c	MK098885	MK098933	—	—	—	USA	Spirin et al. (2019a)
*Myxarium nucleatum*	Spirin 10013	KY801879	KY801906	—	—	—	NORWAY	Spirin et al. (2018)
*Nigrochaete bambusicola*	CLZhao 11307	PQ571172	PQ571167	—	—	—	CHINA	Dong et al. (2025c)
*Nigrochaete bambusicola*	CLZhao 11314	PQ571173	PQ571168	—	—	—	CHINA	Dong et al. (2025c)
*Nodulochaete fissurata*	CLZhao 27533	PQ166573	PQ166565	—	—	—	CHINA	Dong et al. (2025b)
*Nodulochaete fissurata*	CLZhao 27528	PQ166572	PQ166564	—	—	—	CHINA	Dong et al. (2025b)
*Nodulochaete punctata*	CLZhao 22803	PQ166569	PQ166561	—	—	—	CHINA	Dong et al. (2025c)
*Ofella glaira*	VS 11809	MK098920	MK098964	—	—	—	NORWAY	Spirin et al. (2019a)
*Oliveonia fibrillosa*	Spirin 8257	MT235628	MT235607	—	—	—	USA	Spirin et al. (2025)
*Ovipoculum album*	HKAS57058	—	GU292813	—	—	—	CHINA	Kirschner et al. (2010)
*Porpopycnis lubae*	R. Kirschner et al. 2745 (M)	—	HQ914244	—	—	—	PANAMA	Kirschner et al. (2012)
*Proterochaete adusta*	VS 9021	MK391520	MK391528	—	—	—	CANADA	Alvarenga et al. (2019)
*Proterochaete adusta*	CNOM 10519	MK391519	—	—	—	—	CHINA	Alvarenga et al. (2019)
*Protoacia crispans*	Spirin 17688	PV394880	—	—	—	—	SE	Spirin et al. (2025)
*Protoacia delicata*	VS 4615	MK098923	MK098967	—	—	—	RUSSIA	Spirin et al. (2019a)
*Protodaedalea foliacea*	Miettinen 13054	MG757507	MG757507	—	—	—	FINLAND	Spirin et al. (2019b)
*Protodaedalea hispida*	Spirin 5139	MG757510	MG757510	—	—	—	FINLAND	Spirin et al. (2019b)
*Protodontia africana*	AS 171126/1104	MK098978	MK098973	—	—	—	RUSSIA	Spirin et al. (2019a)
*Protohydnum album*	LE 23063	PV241608	PV241622	—	—	—	US	Spirin et al. (2025)
*Protohydnum cartilagineum*	SP 467240	MG735419	MG735426	—	—	—	BRAZIL	Spirin et al. (2019b)
*Protohydnum galzinii*	Otto MiettinenX3067	MG757511	MG757511	—	—	—	SPAIN	Malysheva et al. (2018)
*Protohydnum pululahuana*	KW 1733	—	AF291315	—	—	—	USA	Weiss and Oberwinkler (2001)
*Protomerulius brasiliensis*	Ryv. 19735	—	AF291359	—	—	—	ARGENTINA	Weiss and Oberwinkler (2001)
*Protomerulius substuppeus*	O 19171	JX134482	JQ764649	—	—	—	CHINA	Zhou and Dai (2013)
*Pseudohydnum gelatinosum*	AFTOL ID1875	DQ520094	DQ520094	—	—	—	GERMANY	Lutzoni et al. (2004)
*Psilochaete multifora*	VS 11596	MK484066	MK480576	—	—	MK497246	NORWAY	Spirin et al. (2019b)
*Punctochaete murina*	CLZhao 32118	PP819689	PP819701	—	—	—	CHINA	Dong et al. (2025a)
*Renatobasidium notabile*	VS 11118	MT235654	MT353648	—	—	MT273005	NORWAY	—
*Sclerotrema griseobrunnea*	TN 2722	KX262144	KX262192	—	—	—	CANADA	Malysheva and Spirin (2017)
*Sclerotrema griseobrunnea*	VS 7674	KX262140	KX262188	—	—	—	RUSSIA	Malysheva and Spirin (2017)
*Sebacina incrustans*	MW 573	DQ520095	—	—	—	—	—	—
*Stypella papillata*	KHL 11751	EU118672	—	—	—	—	FINLAND	Larsson (2007)
*Stypellopsis farlowii*	Larsson 12337	MG857095	MG857099	—	—	—	RUSSIA	Spirin et al. (2019c)
*Stypellopsis hyperborea*	J Norden 9751	MG857097	MG857101	—	—	—	RUSSIA	Spirin et al. (2019c)
*Tremellochaete atlantica*	URM90199	MG594381	MG594383	—	—	—	BRAZIL	Alvarenga et al. (2019)
*Tremellochaete japonica*	TAA 42689	AF291274	AF291320	—	—	—	RUSSIA	Weiss and Oberwinkler (2001)
*Tremiscus helvelloides*	MW 337	DQ520100	—	—	—	—	GERMANY	Spirin et al. (2025)

Phylogenetic analyses followed the methods described in Dissanayake et al. (2020). Newly generated sequence data were initially subjected to a BLAST search in NCBI to obtain the most probable closely related taxa in GenBank (http://blast.ncbi.nlm.nih.gov/). Sequence data were retrieved from GenBank based on recent publications (https://www.ncbi.nlm.nih.gov/nuccore/). The sequences were aligned using MAFFT version 7 (Katoh et al. 2019) with the G-INS-I strategy. The alignments were adjusted manually using AliView version 1.27 (Larsson 2014). The dataset of ITS, nrLSU, *RPB*1, *RPB*2, and *TEF*1 sequences was combined using Mesquite version 3.51. FASTA data file formats were converted to PHYLIP and NEXUS formats using the online tool available on the ALTER website (http://sing.ei.uvigo.es/ALTER/, Glez-Peña et al. 2010). Phylogenetic trees were constructed based on randomized accelerated maximum likelihood (ML) and Bayesian inference (BI) analyses. Sequence alignments were deposited at Figshare (https://figshare.com/; submission doi: 10.6084/m9.figshare.32324271).

ML analyses were performed using the CIPRES Science Gateway (https://www.phylo.org/portal2/login!input.action; Miller et al. 2012) based on the dataset using the RAxML-HPC BlackBox tool, with settings to halt bootstrapping automatically, a maximum runtime of 0.25 h, and the best tree obtained using an ML search. Other parameters in the ML analysis were set to default settings, and statistical support values were obtained using nonparametric bootstrapping with 1000 replicates. Bayesian inference analysis was performed on the same dataset using MrBayes v3.2.7a (Ronquist et al. 2012). The best substitution model for the dataset was selected by ModelFinder v2.2.0 (Kalyaanamoorthy et al. 2017) using the Bayesian information criterion, and the model was used for Bayesian analysis. Four Markov chains were run from random starting trees. Trees were sampled every 1000 generations. The first 25% of sampled trees were discarded as burn-in, while the remaining trees were used to construct a 50% majority consensus tree and to calculate Bayesian posterior probabilities (BPPs).

Phylogenetic trees were visualized and adjusted using FigTree v1.4.4 (http://tree.bio.ed.ac.uk/software/figtree), and the exports were edited using Adobe Illustrator CS6 software (Adobe Systems, USA). Branches of the consensus tree with ML bootstrap support equal to or above 70% and BI posterior probabilities equal to or above 0.90 were considered supported.

## Result

### Phylogenetic analyses

The phylogeny of *Auriculariales* based on a five-gene dataset

**Figure 1.**
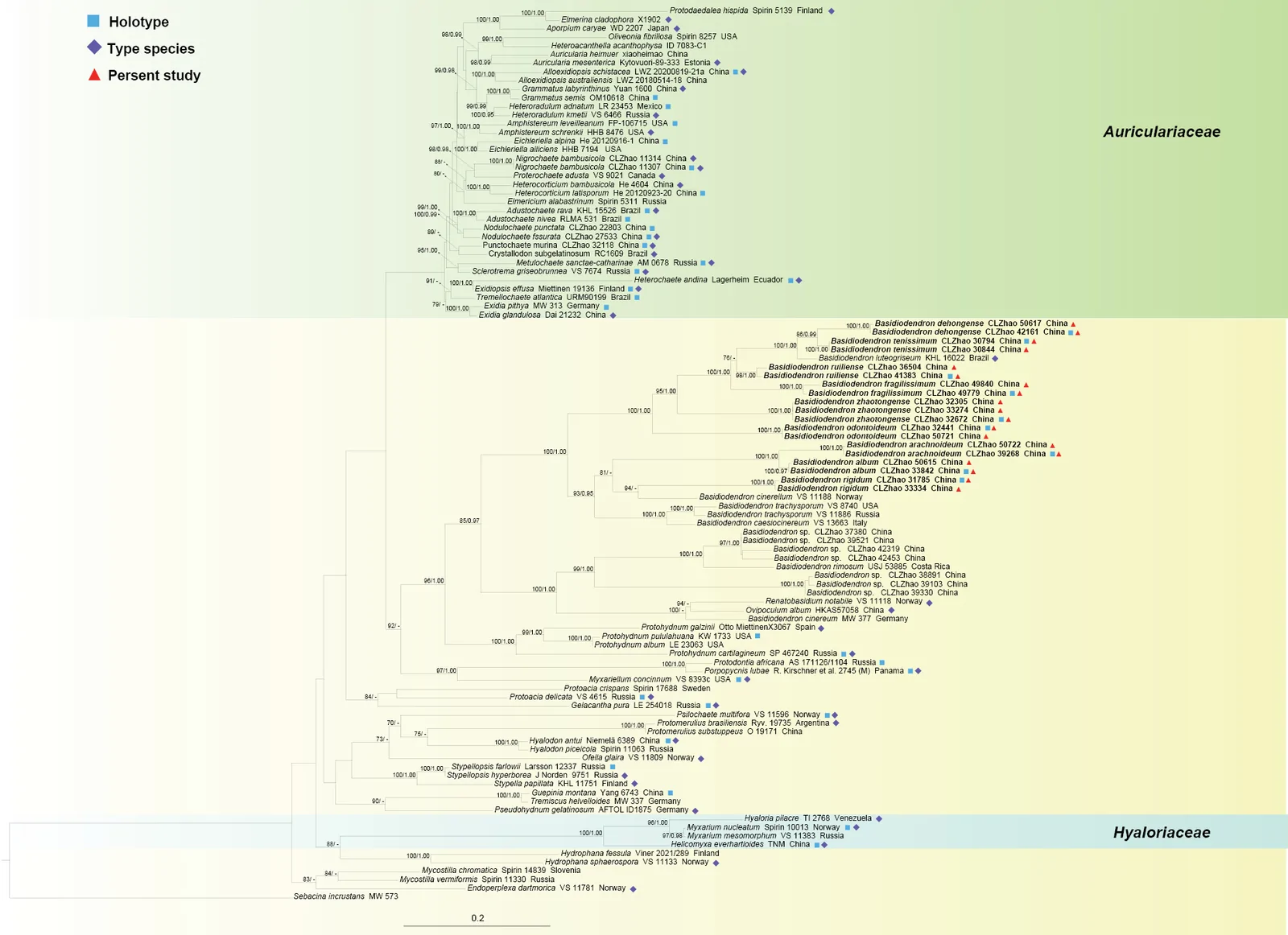
Maximum likelihood phylogenetic tree of *Auriculariales* inferred from ITS+nrLSU+*RPB*1+*RPB*2+*TEF*1 sequence data. *Sebacina incrustans* was used as the outgroup. Branches are labeled with ML bootstrap values ≥ 70% and BPPs ≥ 0.90. Newly described species are highlighted in bold.

The combined ITS+nrLSU+*RPB*1+*RPB*2+*TEF*1 dataset (Fig. 1) included sequences from 103 fungal specimens representing 87 species. Sequences of *Sebacina incrustans* (Pers.) Tul. & C. Tul. belonging to the order *Sebacinales* were retrieved from GenBank and used as the outgroup (Spirin et al. 2025). The best model for this dataset, estimated and applied in the Bayesian analysis, was GTR+I+G, with an average standard deviation of split frequencies of 0.008452 (BI) and an effective sample size (ESS) across the two runs that was double the average ESS (av. ESS = 3155.5). The dataset comprised an aligned length of 4694 characters, of which 2605 were constant, 855 were variable and parsimony-uninformative, and 1234 were parsimony-informative. Four Markov chains were run twice from random starting trees, each for 10 million generations (Fig. 1). The ML analysis resulted in a tree with a log-likelihood value of –38823.760. Bayesian and ML analyses generated final trees with similar topologies.

The phylogram based on the analysis of the combined ITS+nrLSU+*RPB*1+*RPB*2+*TEF*1 sequences (Fig. 1) revealed that nine newly established species, viz., *Basidiodendron album*, *B. arachnoideum*, *B. dehongense*, *B. fragilissimum*, *B. odontoideum*, *B. rigidum*, *B. ruiliense*, *B. tenissimum*, and *B. zhaotongense*, were closely nested with the previously described species in the genus *Basidiodendron*.

#### The phylogeny of *Basidiodendron* based on the combined ITS+nrLSU+*TEF*1 sequence data

**Figure 2.**
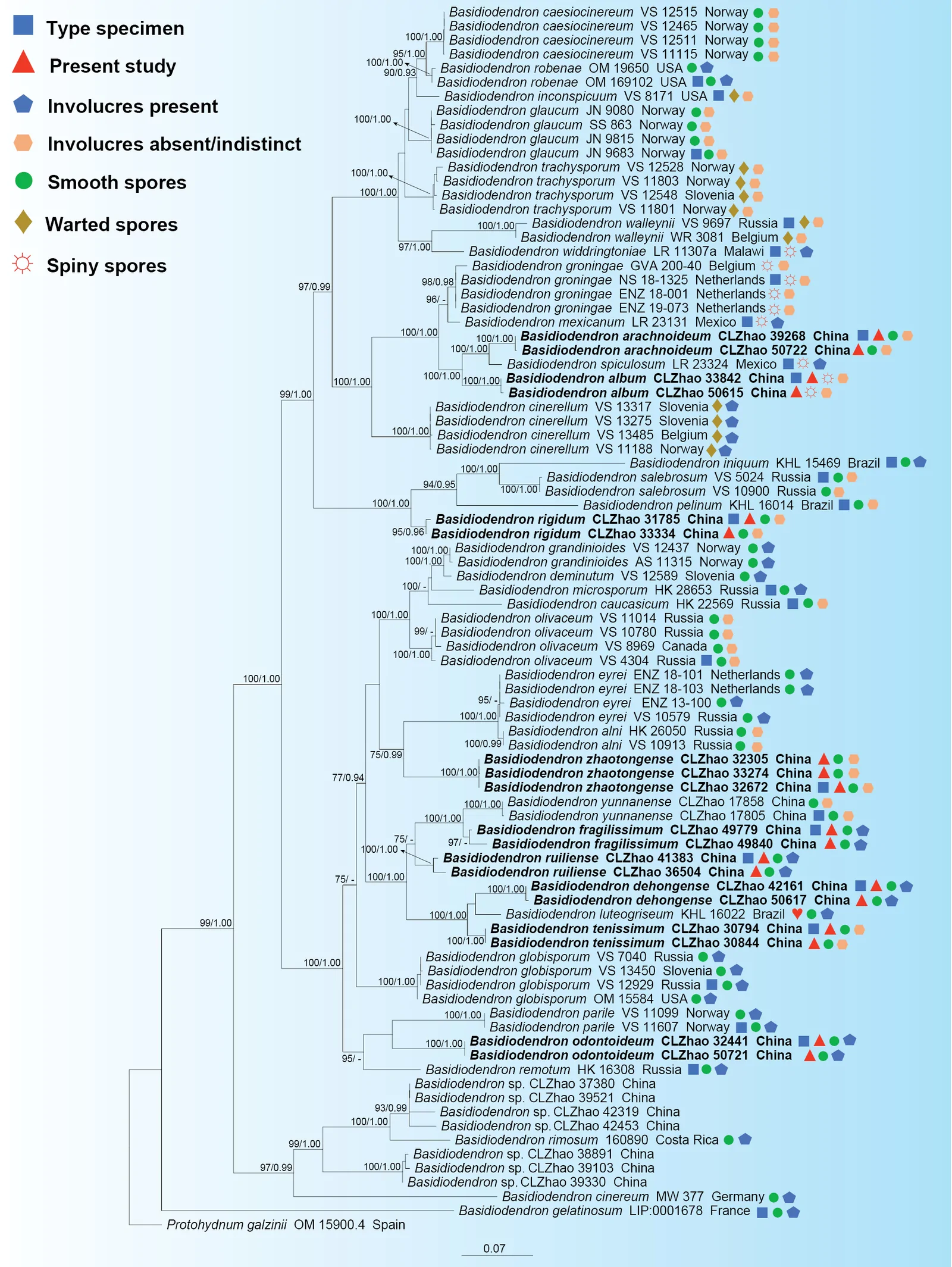
Maximum likelihood phylogenetic tree of nine new species within the genus *Basidiodendron* based on the combined ITS+nrLSU+*TEF*1 sequence data. *Protohydnum galzinii* was used as the outgroup. A total of 41 species were included in the combined analyses. Branches are labeled with ML bootstrap values ≥ 70% and BPPs ≥ 0.90. Newly described species are highlighted in bold.

The combined ITS+nrLSU+*TEF*1 dataset (Fig. 2) included sequences from 87 fungal specimens representing 41 species. Sequences of *Protohydnum galzinii* (Bres.) Trotter were retrieved from GenBank and used as the outgroup (Spirin et al. 2021). The best model for this dataset, estimated and applied in the Bayesian analysis, was GTR+I+G, with an average standard deviation of split frequencies of 0.005317 (BI) and the ESS across the two runs being double the average ESS (av. ESS = 1159.5). The dataset comprised an aligned length of 2581 characters, of which 1453 were constant, 271 were variable and parsimony-uninformative, and 857 were parsimony-informative. Four Markov chains were run twice from random starting trees, each for 3 million generations. The ML analysis resulted in a tree (Fig. 2) with a log-likelihood value of –21714.572. Bayesian and ML analyses resulted in similar tree topologies. The nine new *Basidiodendron* species were recognized in highly supported clades and are described below.

### Taxonomy

#### Basidiodendron

Taxon classificationFungiAuricularialesBasidiomycota

Rick

777B947D-8166-5154-AC58-E24B34301A0A

17148

##### Type species.

*Basidiodendron luteogriseum* Rick.

##### Description.

***Basidiomata*** annual, resupinate, adnate. ***Hymenial surface*** smooth, occasionally tuberculate, pruinose, continuous, becoming crustose, porose, or arachnoid upon drying. ***Hyphal system*** monomitic, generative hyphae with clamp connections. ***Cystidia*** comprising leptocystidia or gloeocystidia, subcylindrical to clavate, flexuous. ***Hyphidia*** present or absent. ***Basidia*** urniform, ovoid to ellipsoid, longitudinally septate, four-celled, involucres well-developed or indistinct. ***Basidiospores*** allantoid, cylindrical, globose to subglobose, often with a small, eccentric and asymmetric apiculus, walls smooth, warted, or spiny (Wells and Raitviir 1975).

##### Notes.

*Basidiodendron* was initially introduced by Rick (1938) as a monotypic genus typified by *B. luteogriseum* Rick. Luck-Allen (1963) reserved the genus for effused wood-inhabiting fungi with gloeocystidia and four-celled, longitudinally septate basidia. *Basidiodendron* is mainly characterized by an involucre-like arrangement of basidia on sinuous hyphae, with older basidia collapsed and stacked in the lower part (Wells and Raitviir 1975).

*Basidiodendron eyrei* (Wakef.) Luck-Allen is a core member of the genus *Basidiodendron* and has recently been thoroughly studied by Spirin et al. (2020). The species was originally described as *Sebacina eyrei* Wakef. based on two specimens characterized in the protologue (Wakefield 1914). Both specimens are deposited at the Kew fungarium (K-M000049485, K-M000049486) and were examined in the present study. The specimen K-M000049485 was collected in Whitby, on decorticated wood, in October 1914 by Miss G.A. Cooper, and it appears to be contaminated with microfungi. In comparison, K-M000049486 is in good condition and contains sufficient material and was selected to serve as the lectotype of *S. eyrei*.

Moreover, in the original description of *Sebacina eyrei*, an affinity to *Bourdotia* was speculated; therefore, it is suggested that the authority for the combination in the latter genus be cited as *Bourdotia eyrei* (Wakef.) Wakef. ex Bourdot & Galzin [vs. *B. eyrei* (Wakef.) Bourdot & Galzin].

#### Basidiodendron eyrei

Taxon classificationFungiAuriculariales

(Wakef.) Luck-Allen

F09A3B4D-E398-5374-8FA5-CCDB99BC98DC

≡ Sebacina eyrei Wakef., Trans. Br. mycol. Soc. 5: 126 (1915). **United Kingdom**, England, Hampshire, Alresford, on decorticated log of Fagus sylvatica, 7.V.1914, comm. Rev. W. Eyre (**lectotype designated here** K, no. K-M000049486; isolectotype PC, no. 0084215). Mycobank typification identifier: MBT 10033155.
= Bourdotia eyrei (Wakef.) Wakef. ex Bourdot & Galzin.

##### Note.

The phylogenetic study of the *Auriculariales* conducted by Weiss and Oberwinkler (2001) showed that five *Basidiodendron* species formed a single cluster, although without statistical support. As discussed below, the monophyly of *Basidiodendron* still cannot be confirmed.

#### Basidiodendron album

Taxon classificationFungiAuricularialesBasidiomycota

Q. Yuan & C.L. Zhao sp. nov.

9CC10672-DBDB-513C-84F6-B350F3BDD381

864138

Figs 3, 4, 5

##### Diagnosis.

*Basidiodendron album* differs from other species in the genus by its smooth, white when fresh, white to cream upon drying hymenial surface and spiny, globose basidiospores.

**Figure 3.**
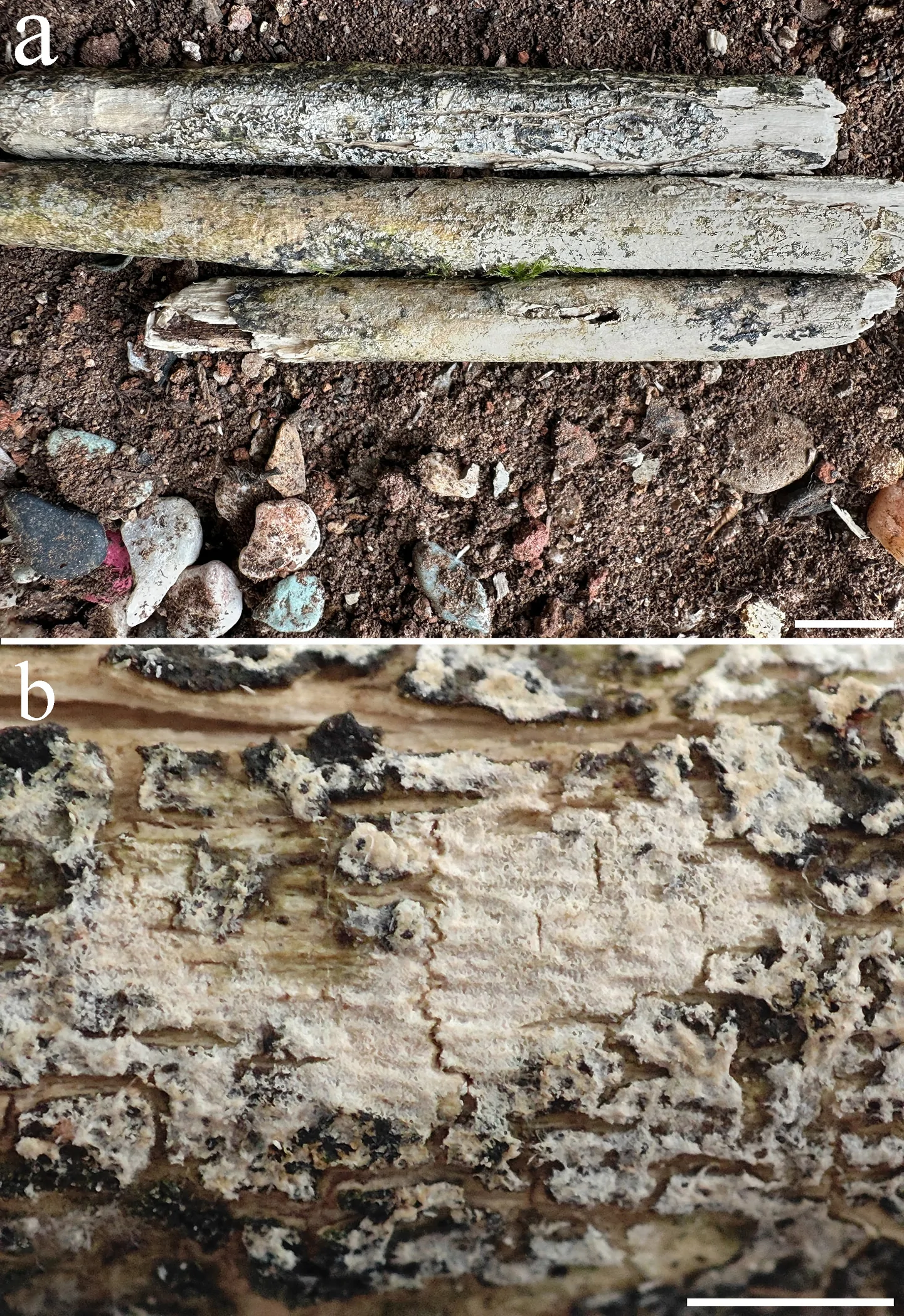
Basidiomata of *Basidiodendron album* (holotype CLZhao 33842). Scale bars: 1 cm (**a**); 2 mm (**b**).

**Figure 4.**
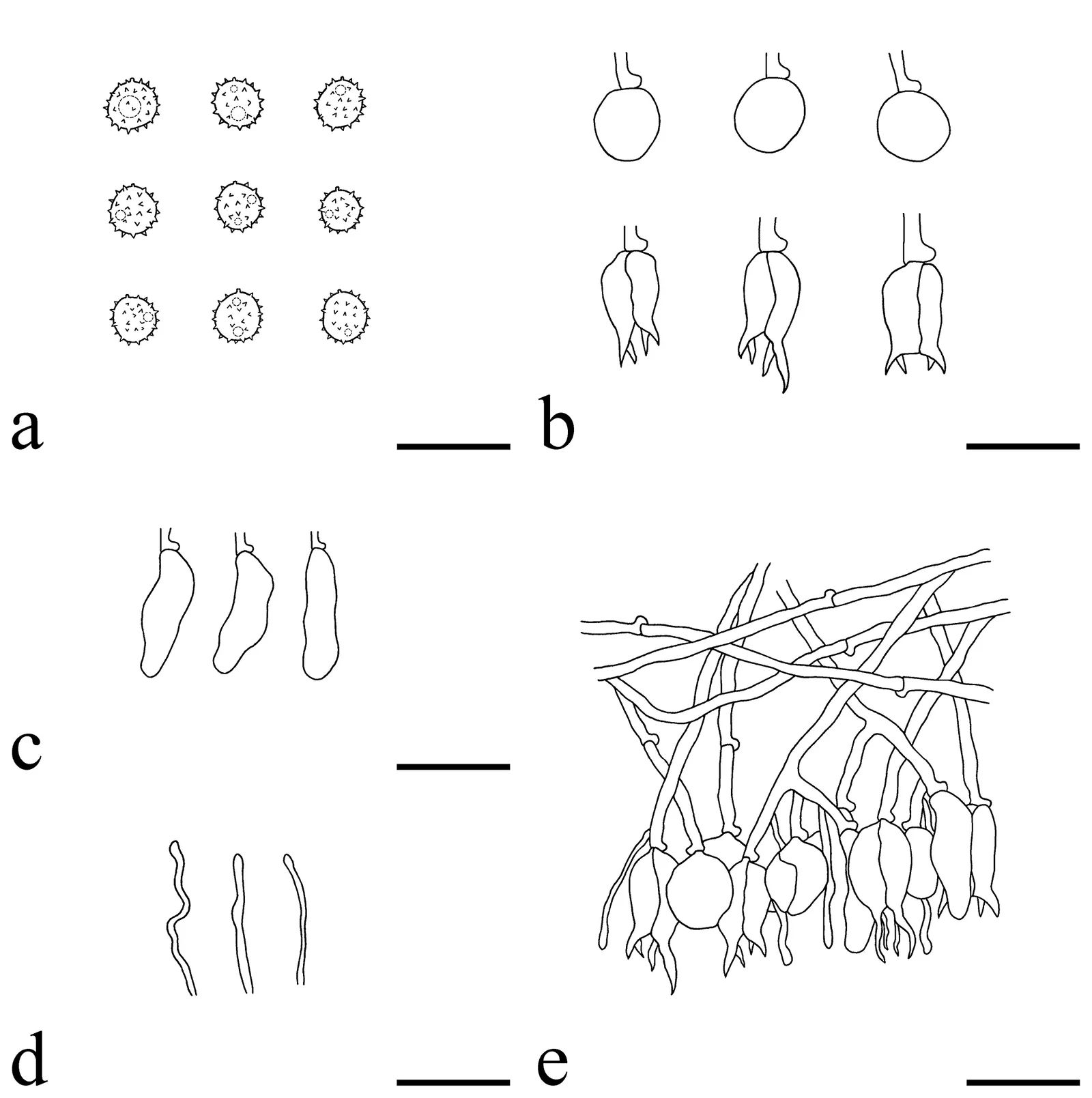
Microscopic structures of *Basidiodendron album* (holotype CLZhao 33842). **a** Basidiospores; **b** basidia and basidioles; **c** leptocystidia; **d** hyphidia; **e** part of a vertical section of the hymenium. Scale bars: 20 µm (**a–e**).

**Figure 5.**
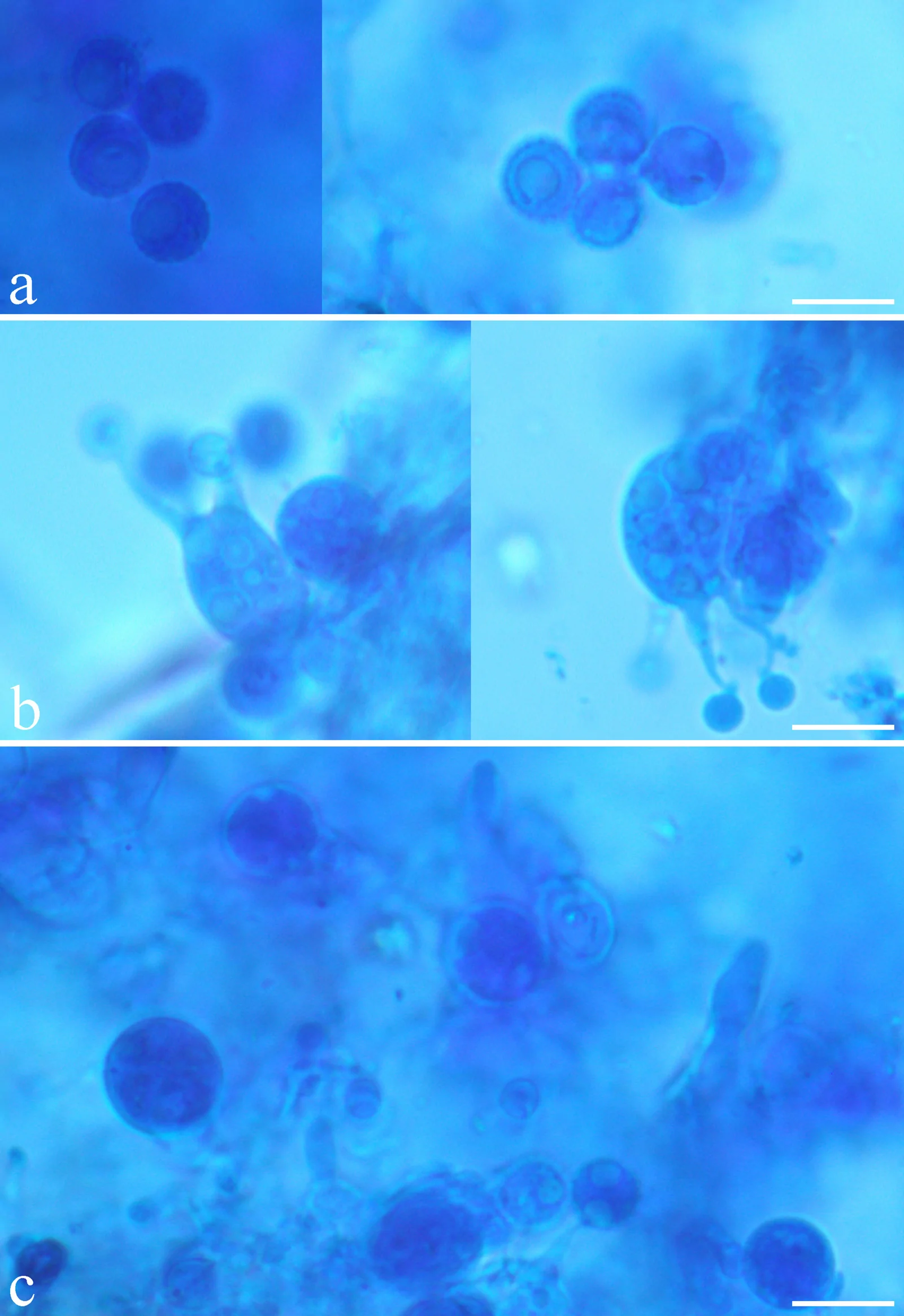
Sections of the hymenium of *Basidiodendron album* (holotype CLZhao 33842). **a** Basidiospores; **b** basidioles; **c** basidia and cystidia. Scale bars: 10 µm (**a–c**).

##### Etymology.

*Album* (Lat.). Referring to the white basidiomata of the type specimen.

##### Type.

CHINA • Yunnan Province, Zhaotong, Xiaocaoba Town, Wumengshan National Nature Reserve, 27°77'N, 104°29'E, elevation 2,900 m asl., on fallen angiosperm branch, leg. C.L. Zhao, 21 Sep. 2023, CLZhao 33842 (SWFC 00033842, holotype).

##### Description.

***Basidiomata*** annual, resupinate, closely adnate, membranous, without odor or taste when fresh, up to 6 cm long, 1 cm wide, and 50–100 µm thick. ***Hymenial surface*** smooth, white when fresh, white to cream upon drying. ***Sterile margin*** narrow, white, up to 1 mm. ***Hyphal system*** monomitic; generative hyphae with clamp connections, colorless, thin-walled, smooth, rarely branched, interwoven, 1.5–2.5 µm in diameter, IKI–, CB–; tissues unchanged in KOH. ***Leptocystidia*** clavate, colorless, thin-walled, smooth, 8.5–21.5 × 3–5.5 µm. ***Hyphidia*** arising from generative hyphae, nodulose, rarely branched, colorless, thin-walled, 1–2.5 µm in diameter. ***Basidia*** narrowly ovoid to ellipsoid, longitudinally septate, four-celled, 14–15.5 × 9–11.5 µm, involucres absent. ***Basidioles*** dominant, similar to basidia in shape, but slightly smaller. ***Basidiospores*** globose, colorless, thin-walled, spiny, usually with oil drops, IKI–, CB–, 7–9(–10) × (6–)6.5–8.5(–9) µm, *L* = 8.10 µm, *W* = 7.57 µm, *Q* = 1.07–1.12 (*n* = 60/2).

##### Additional specimen examined (Paratype).

CHINA • Yunnan Province, Zhaotong, Xiaocaoba Town, Wumengshan National Nature Reserve, 27°47'N, 103°51'E, elevation 1,650 m asl., on fallen angiosperm branch, leg. C.L. Zhao, 21 Sep. 2023, CLZhao 50615 (SWFC 00050615).

##### Notes.

The combined ITS + nrLSU + *TEF*1 phylogenetic analysis revealed that *Basidiodendron album* (CLZhao 33842, CLZhao 50615) formed a single lineage with strong support (100% ML, 1.00 BPP) and grouped with *B. arachnoideum* and *B. spiculosum* (L.S. Olive) Wojewoda (Fig. 2). However, *B. arachnoideum* can be distinguished from *B. album* by its arachnoid to farinaceous basidiomata with a grandinioid hymenial surface and its smaller basidiospores (3.5–4 × 3–3.5 µm vs. 7–9 × 6.5–8.5 µm). The key characters distinguishing *B. spiculosum* from *B. album* are its waxy basidiomata with a pruinose-reticulate hymenial surface and larger cystidia (28–51 × 6.8–10 µm vs. 8.5–21.5 × 3–5.5 µm; Spirin et al. 2021). Morphologically, *B. album* resembles three taxa, viz., *B. alni* Spirin & Malysheva, *B. caucasicum* Spirin, Malysheva & Kotiranta, and *B. cinerellum* (Bourdot & Galzin) Spirin & Malysheva, in having a smooth hymenial surface. However, *B. alni* differs from *B. album* by its pruinose basidiomata and smaller basidiospores (4.1–5 × 4.2–5 µm vs. 7–9 × 6.5–8.5 µm; Spirin et al. 2020). *Basidiodendron caucasicum* can be separated from *B. album* by its reticulate basidiomata and smaller basidiospores (4–5.1 × 3.4–4.5 µm vs. 7–9 × 6.5–8.5 µm; Spirin et al. 2020). *Basidiodendron cinerellum* is distinct from *B. album* in having pruinose-reticulate basidiomata and shorter basidiospores (4.9–7.2 × 5.2–7.7 µm vs. 7–9 × 6.5–8.5 µm; Spirin et al. 2021).

#### Basidiodendron arachnoideum

Taxon classificationFungiAuricularialesBasidiomycota

Q. Yuan & C.L. Zhao sp. nov.

73A69712-FE08-569E-9709-FF2BDC125C83

864140

Figs 6, 7, 8

##### Diagnosis.

*Basidiodendron arachnoideum* is characterized by its arachnoid to farinaceous basidiomata, grandinoid and white to slightly cream hymenial surface, as well as ellipsoid to short clavate basidia lacking longitudinal septa.

**Figure 6.**
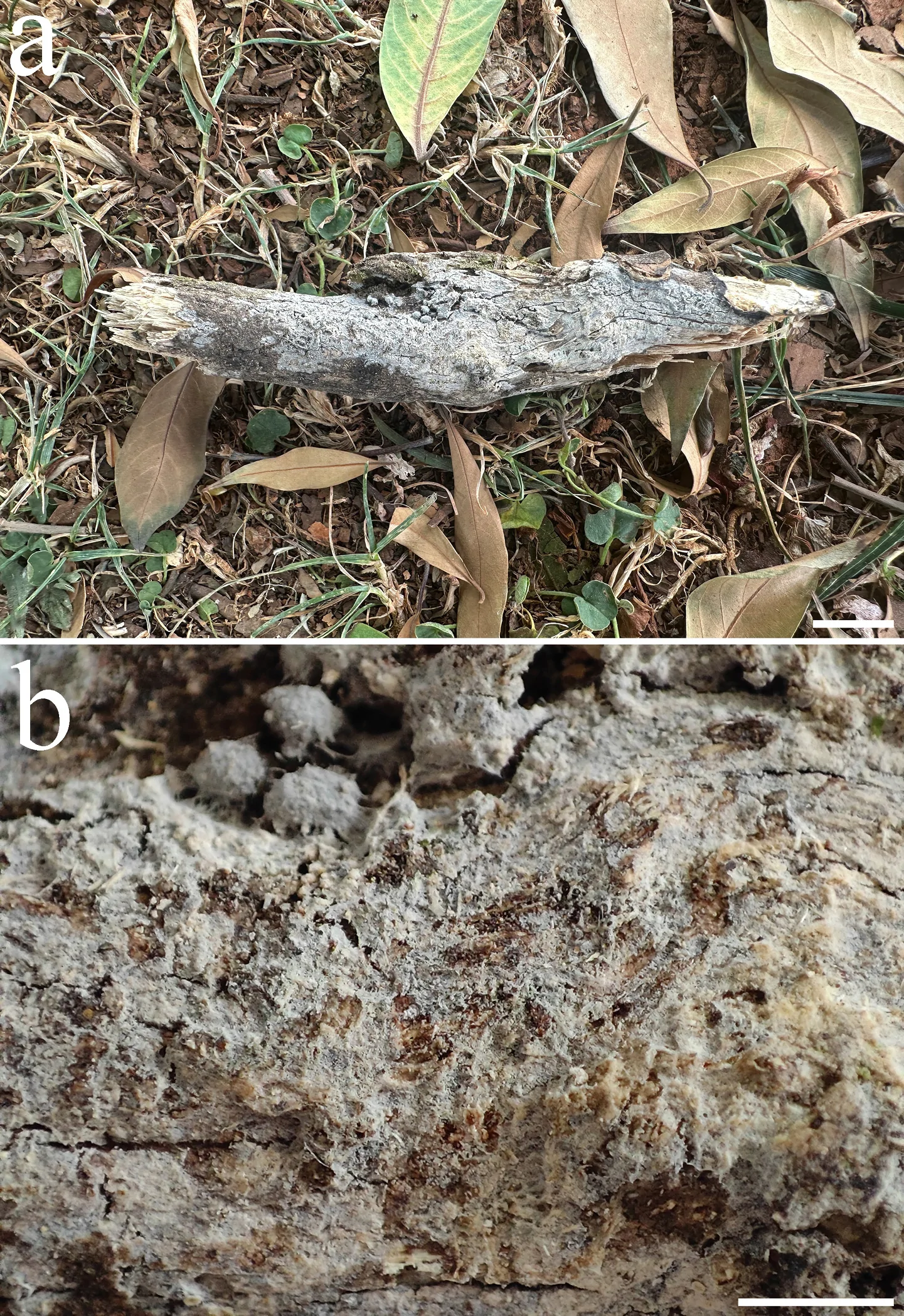
Basidiomata of *Basidiodendron arachnoideum* (holotype CLZhao 39268). Scale bars: 1 cm (**a**); 2 mm (**b**).

**Figure 7.**
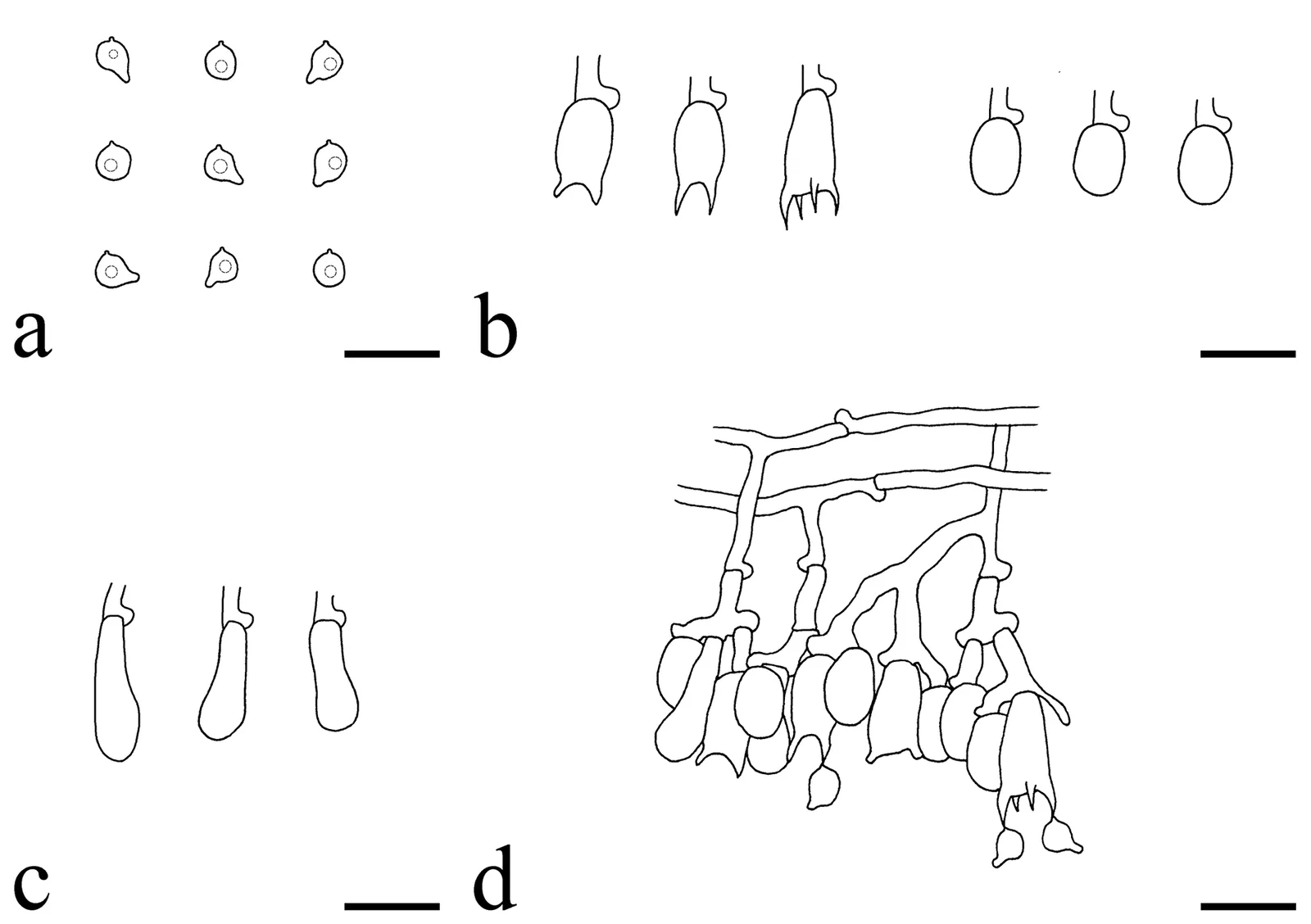
Microscopic structures of *Basidiodendron arachnoideum* (holotype CLZhao 39268). **a** Basidiospores; **b** basidia and basidioles; **c** leptocystidia; **d** part of a vertical section of the hymenium. Scale bars: 10 µm (**a–d**).

**Figure 8.**
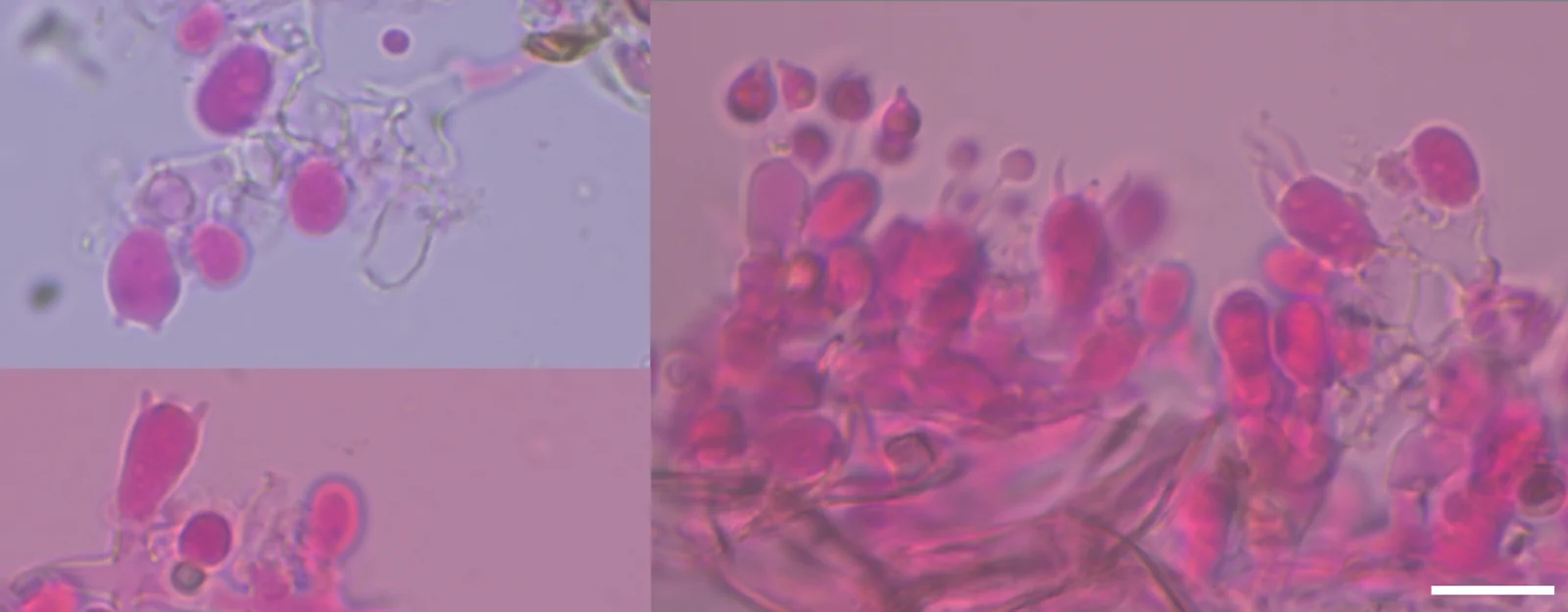
Sections of the hymenium of *Basidiodendron arachnoideum* (holotype CLZhao 39268). Scale bars: 10 µm.

##### Etymology.

*Arachnoideum* (Lat.). Referring to the arachnoid hymenial surface of the type specimen.

##### Type.

CHINA • Yunnan Province, Tengchong, Tuantian Town, Gaoligong Mountain National Nature Reserve, 24°43'N, 98°33'E, elevation 1,360 m asl., on fallen angiosperm branch, leg. C.L. Zhao, 7 Jul. 2024, CLZhao 39268 (SWFC 00039268, holotype).

##### Description.

***Basidiomata*** annual, resupinate, adnate, arachnoid to farinaceous, without odor or taste when fresh, up to 9 cm long, 4 cm wide, and up to 100 µm thick. ***Hymenial surface*** grandinoid, white when fresh, turning white to slightly cream upon drying. ***Sterile margin*** narrow, white, up to 1 mm. ***Hyphal system*** monomitic; generative hyphae with clamp connections, colorless, thin-walled, smooth, interwoven, 1–1.5 µm in diameter, IKI–, CB–; tissues unchanged in KOH. ***Leptocystidia*** clavate, colorless, thin-walled, smooth, 9.5–12 × 2.5–4 µm. ***Basidia*** ellipsoid to short clavate, with four sterigmata and a basal clamp connection, 7.5–8.5 × 5–5.5 µm, involucres absent. ***Basidioles*** dominant, similar to basidia in shape, but slightly smaller. ***Basidiospores*** subglobose, with a short, often eccentric and asymmetric apiculus, colorless, thin-walled, smooth, usually with one oil drop, IKI–, CB–, (3–)3.5–4 × 3–3.5 µm, *L* = 3.55 µm, *W* = 3.28 µm, *Q* = 1.08–1.10 (*n* = 60/2).

##### Additional specimen examined (Paratype).

CHINA • Yunnan Province, Tengchong, Tuantian Town, Gaoligong Mountain National Nature Reserve, 26°56'N, 98°42'E, elevation 1,580 m asl., on fallen angiosperm branch, leg. C.L. Zhao, 7 Jul. 2024, CLZhao 50722 (SWFC 00050722).

##### Notes.

The combined ITS+nrLSU+*TEF*1 phylogenetic analysis revealed that *Basidiodendron arachnoideum* was resolved as sister to *B. spiculosum* (Fig. 2). However, *B. spiculosum* can be delimited from *B. arachnoideum* by its waxy basidiomata with a smooth hymenial surface and spiny, larger basidiospores (6.9–8.2 × 7.1–8.9 µm vs. 3.5–4 × 3–3.5 µm; Spirin et al. 2021). Morphologically, *B. arachnoideum* resembles several taxa, viz., *B. alni*, *B. cinerellum*, and *B. glaucum* Spirin & K.H. Larss., in having farinaceous basidiomata. However, *B. alni* is distinct from *B. arachnoideum* by having a smooth hymenial surface and larger basidiospores (4.1–5 × 4.2–5 µm vs. 3.5–4 × 3–3.5 µm; Spirin et al. 2020). *Basidiodendron cinerellum* differs from *B. arachnoideum* by its smooth hymenial surface and larger basidiospores (4.9–7.2 × 5.2–7.7 µm vs. 3.5–4 × 3–3.5 µm; Spirin et al. 2021). *Basidiodendron glaucum* can be separated from *B. arachnoideum* by its smooth hymenial surface and larger basidiospores (5.1–6.8 × 5.2–7 µm vs. 3.5–4 × 3–3.5 µm; Spirin et al. 2021).

The generic concept of *Basidiodendron* has historically relied heavily on specific basidial morphologies, particularly the presence of longitudinally septate basidia. Interestingly, re-examination of *B. arachnoideum* revealed an absence of longitudinal septa in its mature basidia (Fig. 8). While this might traditionally exclude it from the genus, the phylogeny inferred from the ITS+nrLSU+*TEF*1 sequence data securely nests *B. arachnoideum* within the well-supported main *Basidiodendron* clade (Fig. 2). As demonstrated in the phylogenetic tree, this species forms a distinct lineage separate from the type species. This finding indicates that structural features such as basidial septation may undergo secondary loss during the evolutionary radiation of specific clades within the genus. Consequently, molecular phylogeny provides a more reliable basis for the generic placement of such morphologically atypical taxa than reliance solely on individual morphological traits.

#### Basidiodendron dehongense

Taxon classificationFungiAuricularialesBasidiomycota

Q. Yuan & C.L. Zhao sp. nov.

F293B30C-F587-5204-8785-1A496A9447C4

864141

Figs 9, 10, 11

##### Diagnosis.

*Basidiodendron dehongense* differs from other species by its slightly olivaceous to buff hymenial surface, subclavate cystidia, and broadly ellipsoid to subglobose basidiospores.

**Figure 9.**
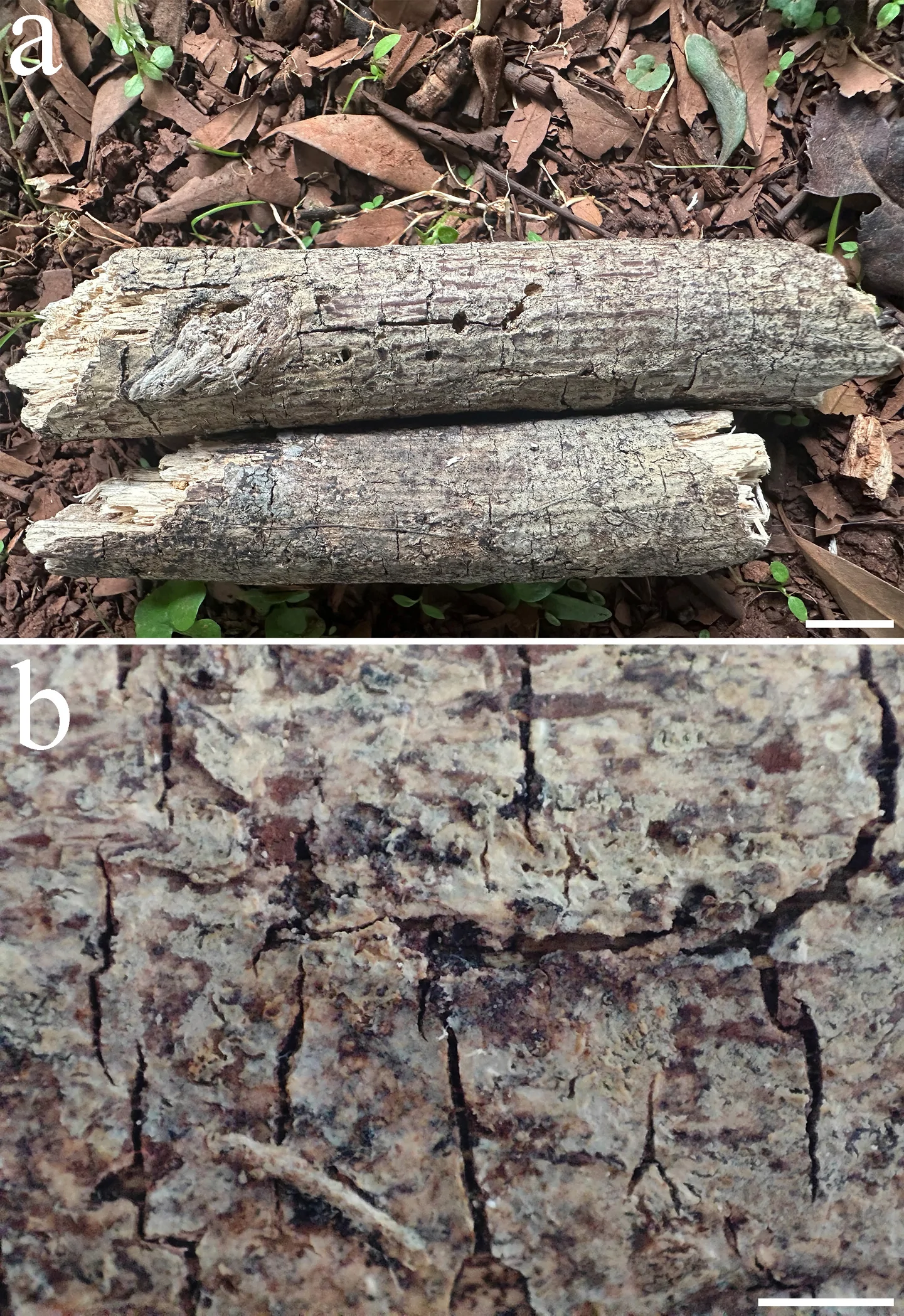
Basidiomata of *Basidiodendron dehongense* (holotype CLZhao 42161). Scale bars: 1 cm (**a**); 2 mm (**b**).

**Figure 10.**
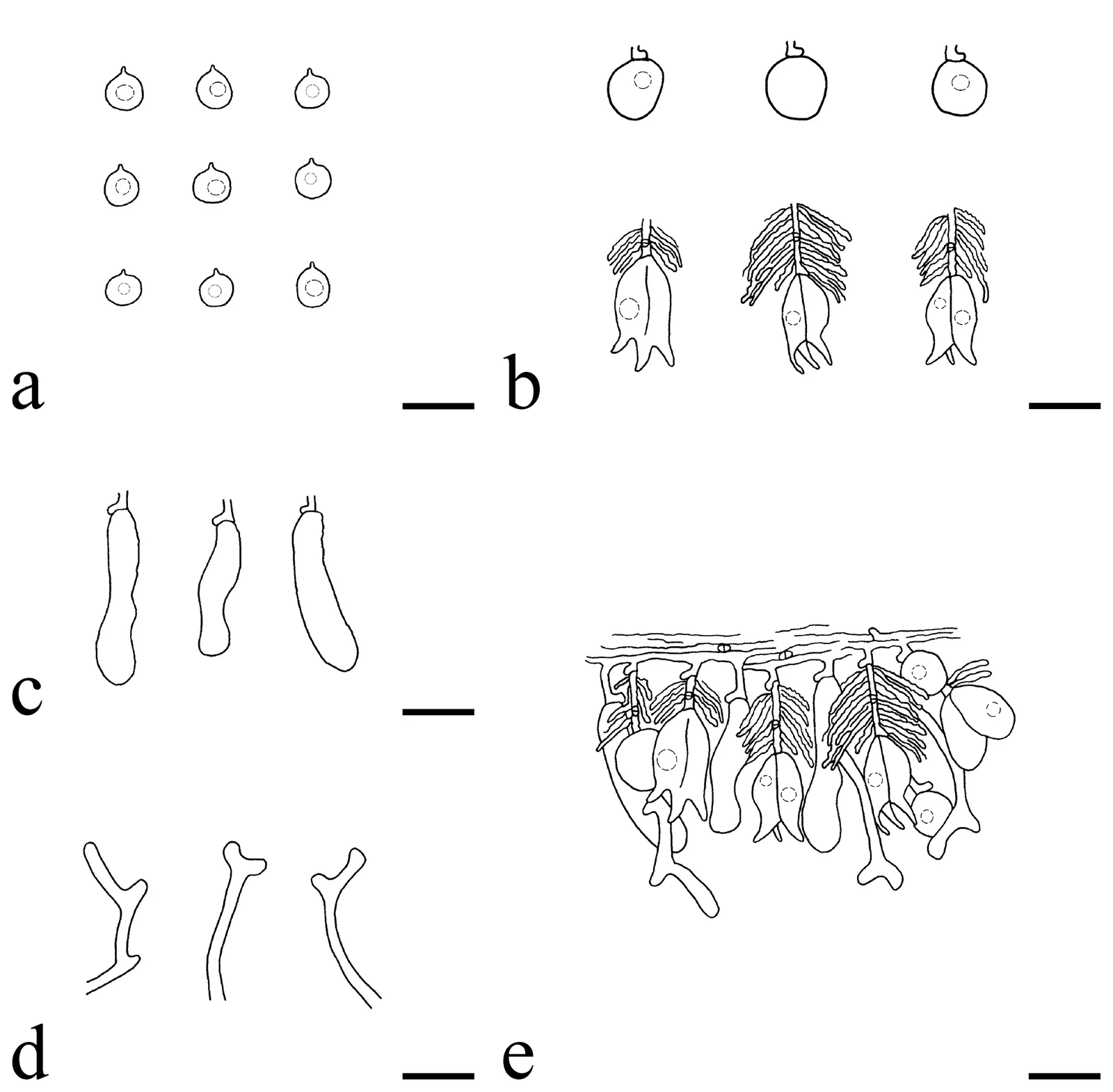
Microscopic structures of *Basidiodendron dehongense* (holotype CLZhao 42161). **a** Basidiospores; **b** basidia and basidioles; **c** leptocystidia; **d** hyphidia; **e** part of a vertical section of the hymenium. Scale bars: 10 µm (**a–e**).

**Figure 11.**
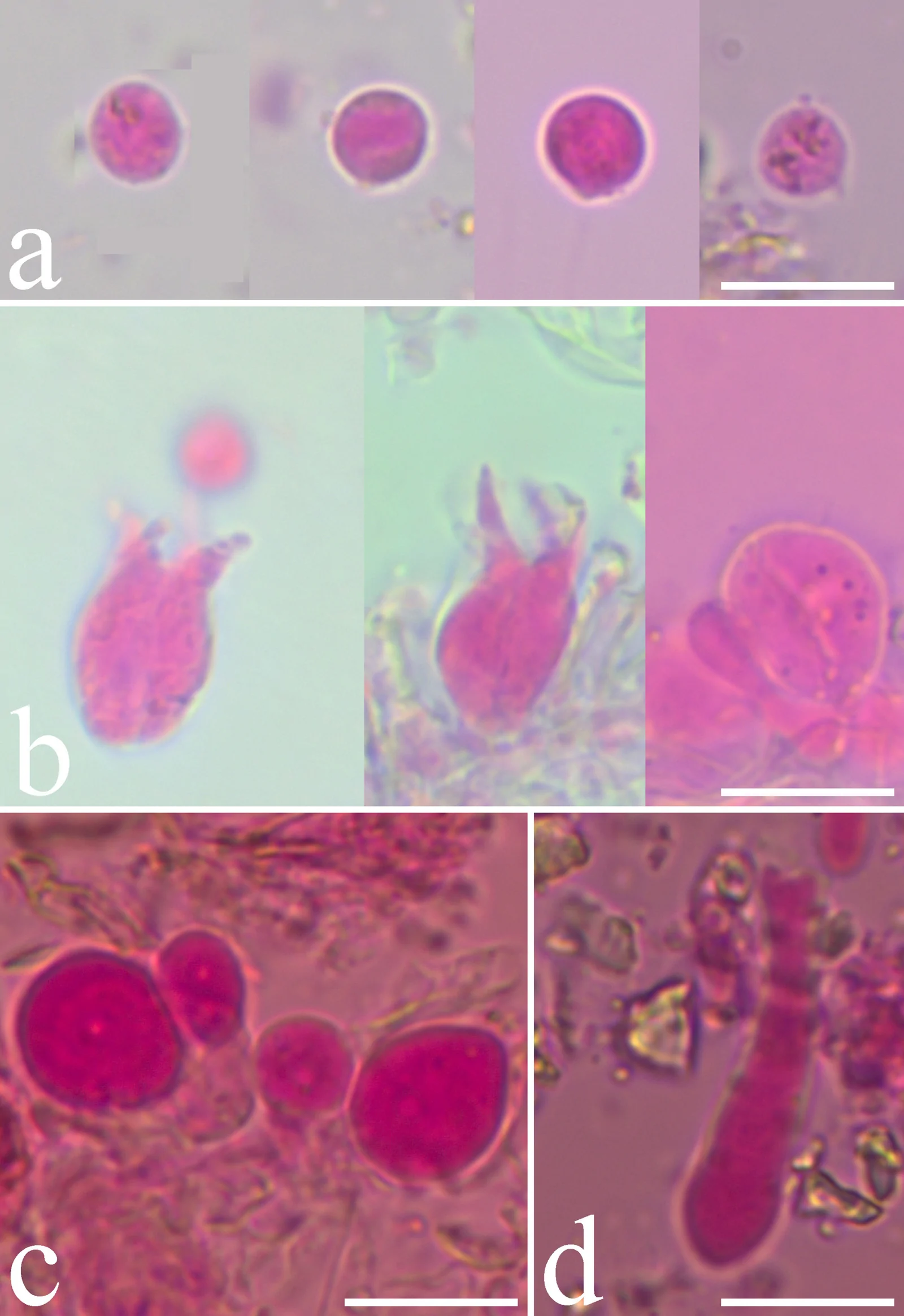
Sections of the hymenium of *Basidiodendron dehongense* (holotype CLZhao 42161). **a** Basidiospores; **b** basidia; **c** basidioles; **d** leptocystidia. Scale bars: 10 µm (**a–d**).

##### Etymology.

*Dehongense* (Lat.). Referring to the type locality, Dehong, Yunnan Province, China.

##### Type.

CHINA • Yunnan Province, Dehong, Ruili City, Tongbiguan Provincial Nature Reserve, 24°11'N, 97°31'E, elevation 1,945 m asl., on fallen angiosperm branch, leg. C.L. Zhao, 3 Nov. 2024, CLZhao 42161 (SWFC 00042161, holotype).

##### Description.

***Basidiomata*** annual, resupinate, closely adnate, membranaceous, without odor or taste when fresh, up to 10 cm long, 5 cm wide, and up to 100 µm thick. ***Hymenial surface*** smooth, slightly olivaceous to buff when fresh, turning to buff to cream upon drying. ***Sterile margin*** narrow, buff, up to 1 mm. ***Hyphal system*** monomitic; generative hyphae with clamp connections, colorless, thin-walled, smooth, interwoven, 2–4 µm in diameter, IKI–, CB–; tissues unchanged in KOH. ***Leptocystidia*** subclavate, colorless, thin-walled, smooth, 18.5–28 × 3.5–6.5 µm. ***Hyphidia*** arising from generative hyphae, nodulose, rarely branched, colorless, thin-walled, 1–2.5 µm in diameter. ***Basidia*** ellipsoid to ovoid, longitudinally septate, two- to four-celled, 10.5–13.5 × 7–10 µm, involucres well developed, often totally covering basidial cells. ***Basidioles*** dominant, similar to basidia in shape, but slightly smaller. ***Basidiospores*** broadly ellipsoid to subglobose, colorless, thin-walled, smooth, usually with one or more oil drops, IKI–, CB–, 5.5–7.5 × 4.5–6.5 µm, *L* = 6.46 µm, *W* = 5.33 µm, *Q* = 1.17–1.21 (*n* = 60/2).

##### Additional specimen examined (Paratype).

CHINA • Yunnan Province, Dehong, Ruili City, Tongbiguan Provincial Nature Reserve, 25°21'N, 98°06'E, elevation 1,600 m asl., on fallen angiosperm branch, leg. C.L. Zhao, 3 Nov. 2024, CLZhao 50617 (SWFC 00050617).

##### Notes.

*Basidiodendron dehongense* was shown to group within *Basidiodendron* in a sister relationship to *B. luteogriseum* in the combined ITS+nrLSU+*TEF*1 phylogenetic inference (Fig. 2). However, *B. luteogriseum* differs from *B. dehongense* by its pruinose hymenial surface and shorter basidiospores (4.1–5.1 × 4–5.1 µm vs. 5.5–7.5 × 4.5–6.5 µm; Spirin et al. 2020). Morphologically, *B. dehongense* resembles *B. cinerellum*, *B. groningae* Schoutt. & Spirin, and *B. inconspicuum* Spirin & Malysheva by having a smooth hymenial surface. However, *B. cinerellum* can be separated from *B. dehongense* by its pruinose basidiomata and warted basidiospores (Spirin et al. 2021). *Basidiodendron groningae* is distinct from *B. dehongense* by its pruinose-reticulate basidiomata and spiny, narrower basidiospores (6–7.9 × 6.2–8.2 µm vs. 5.5–7.5 × 4.5–6.5 µm; Spirin et al. 2021). *Basidiodendron inconspicuum* is separated from *B. dehongense* by its pruinose-reticulate basidiomata and minutely warted basidiospores (Spirin et al. 2021).

#### Basidiodendron fragilissimum

Taxon classificationFungiAuricularialesBasidiomycota

Q. Yuan & C.L. Zhao sp. nov.

968FC994-FDF0-5BF4-97CA-B4731863C419

864142

Figs 12, 13, 14

##### Diagnosis.

*Basidiodendron fragilissimum* differs from other species by its olivaceous, smooth hymenial surface, broadly globose to subglobose with a short, often eccentric and asymmetric apiculus basidiospores.

**Figure 12.**
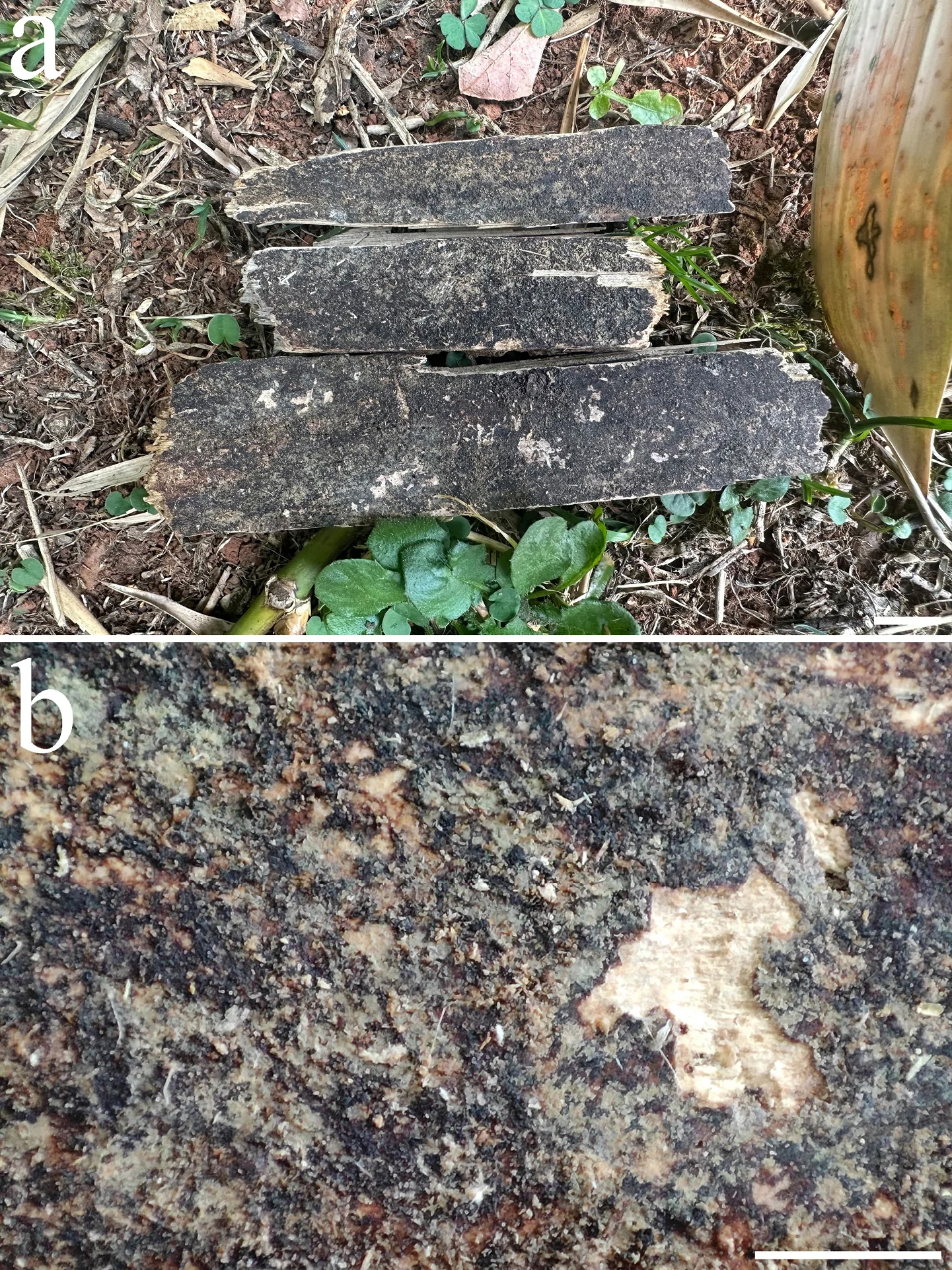
Basidiomata of *Basidiodendron fragilissimum* (holotype CLZhao 49840). Scale bars: 1 cm (**a**); 2 mm (**b**).

**Figure 13.**
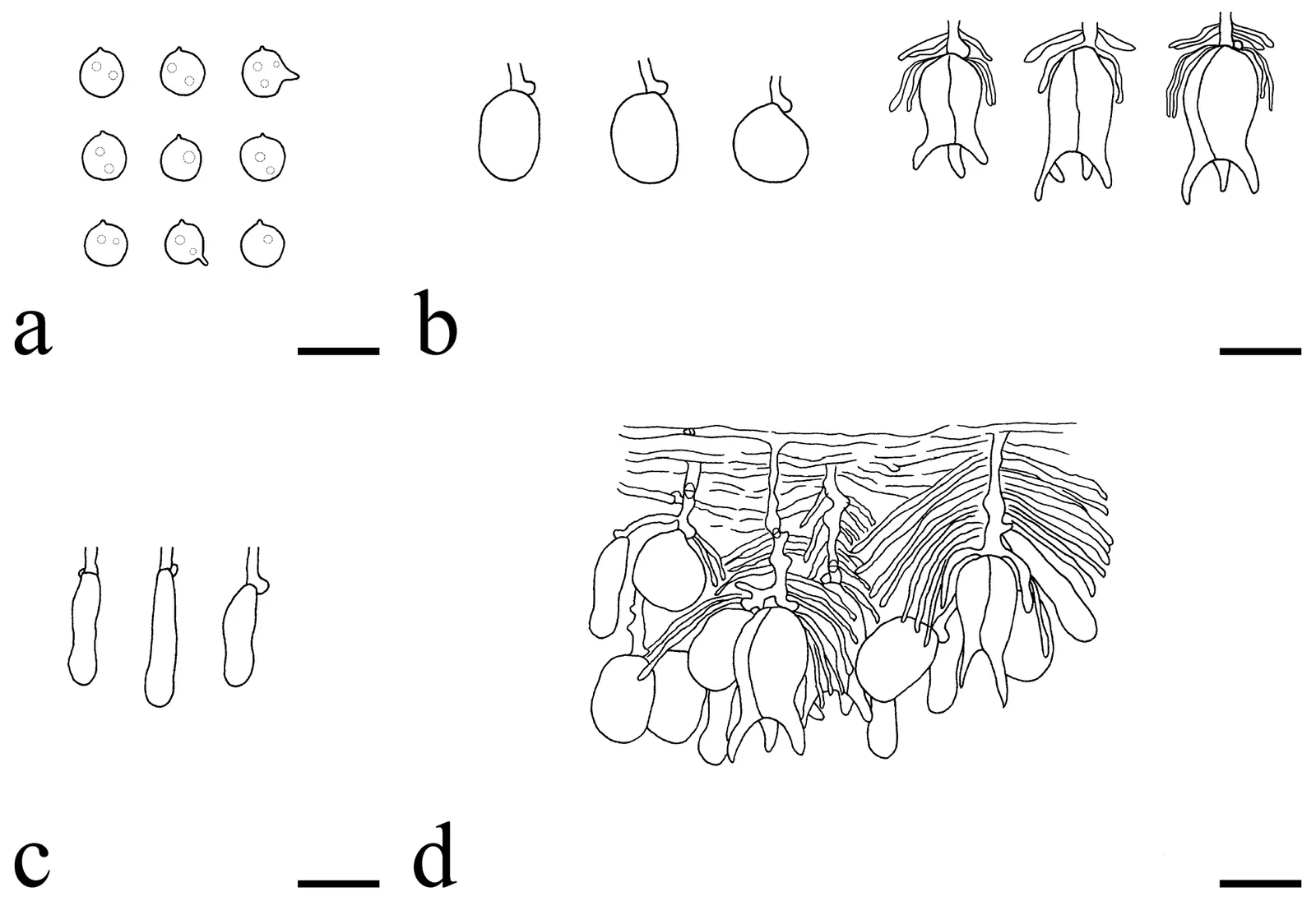
Microscopic structures of *Basidiodendron fragilissimum* (holotype CLZhao 49840). **a** Basidiospores; **b** basidia and basidioles; **c** leptocystidia; **d** part of a vertical section of the hymenium. Scale bars: 10 µm (**a–d**).

**Figure 14.**
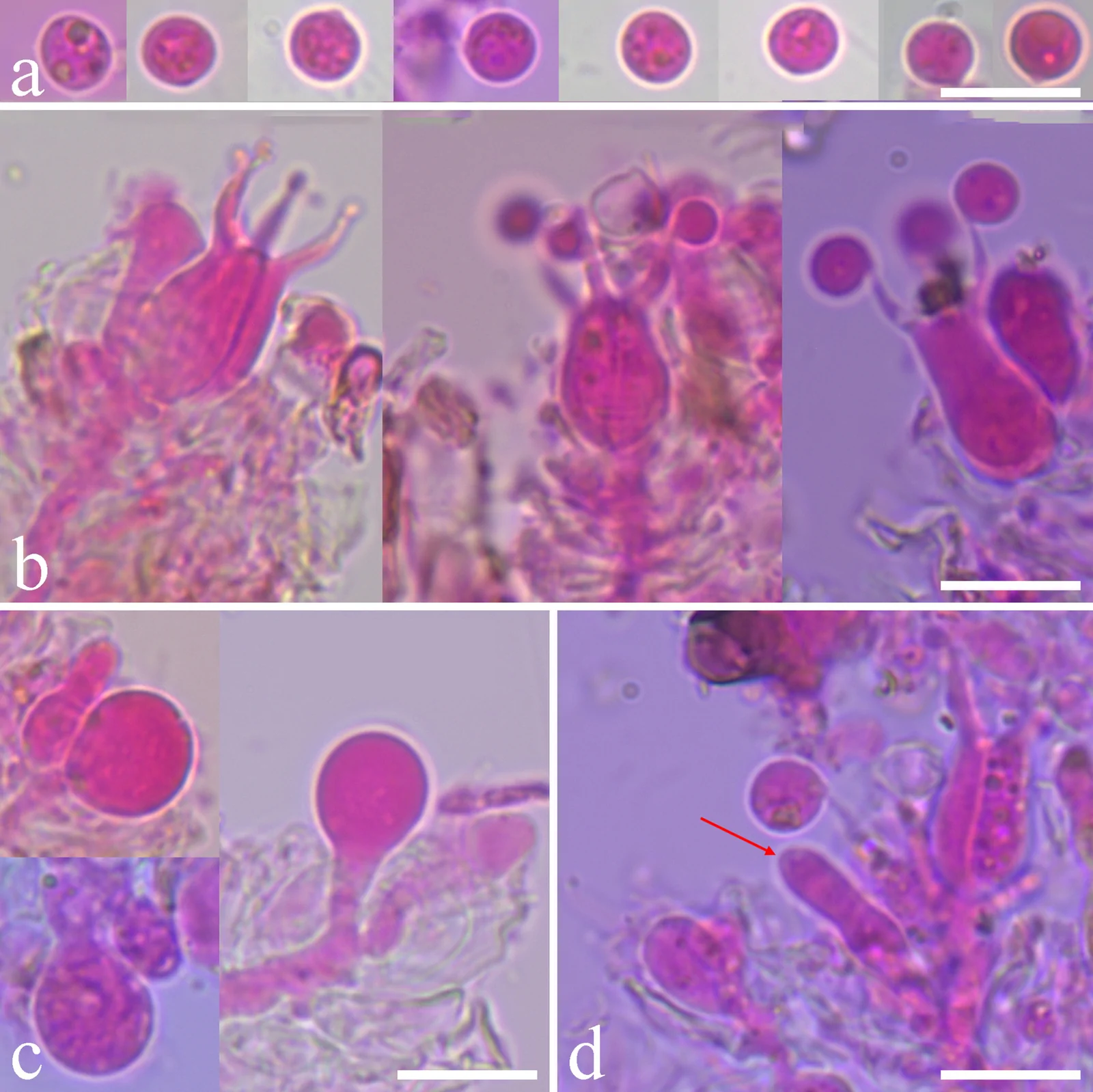
Sections of the hymenium of *Basidiodendron fragilissimum* (holotype CLZhao 49840). **a** Basidiospores; **b** basidia; **c** basidioles; **d** leptocystidia. Scale bars: 10 µm (**a–d**).

##### Etymology.

*Fragilissimum* (Lat.). Referring to the fragile basidiomata of the type specimen.

##### Type.

CHINA • Yunnan Province, Dehong, Longchuan County, Tongbiguan Provincial Reserve, 24°32'N, 97°68'E, elevation 1,807 m asl., on dead bamboo, leg. C.L. Zhao, 3 Oct. 2025, CLZhao 49840 (SWFC 00049840, holotype).

##### Description.

***Basidiomata*** annual, resupinate, closely adnate, membranaceous, without odor or taste when fresh, up to 15 cm long, 7 cm wide, and up to 50 µm thick. ***Hymenial surface*** smooth, slightly olivaceous when fresh, turning to slightly olivaceous to slightly brown upon drying, fragile. ***Sterile margin*** narrow, olivaceous, up to 1 mm. ***Hyphal system*** monomitic; generative hyphae with clamp connections, colorless, thin-walled, smooth, interwoven, 1–2.5 µm in diameter, IKI–, CB–; tissues unchanged in KOH. ***Leptocystidia*** subclavate, colorless, thin-walled, smooth, 9–15.5 × 2.5–5 µm. ***Hyphidia*** arising from generative hyphae, nodulose, frequently branched, colorless, thin-walled, 1–2 µm in diameter. ***Basidia*** ellipsoid to ovoid, longitudinally septate, two- to four-celled, 10–13 × 7–9 µm, involucres well developed, often totally covering basidial cells. ***Basidioles*** dominant, similar to basidia in shape, but slightly smaller. ***Basidiospores*** broadly globose to subglobose with a short, often eccentric and asymmetric apiculus, colorless, thin-walled, smooth, usually with one or more oil drops, IKI–, CB–, (4–)4.5–5.5 × 4–5(–5.5) µm, *L* = 5.00 µm, *W* = 4.74 µm, *Q* = 1.05–1.06 (*n* = 60/2).

##### Additional specimen examined (Paratype).

CHINA • Yunnan Province, Dehong, Longchuan County, Tongbiguan Provincial Reserve, 24°08'N, 97°39'E, elevation 2,218 m asl., on dead bamboo, leg. C.L. Zhao, 3 Oct. 2025, CLZhao 49779 (SWFC 00049779).

##### Notes.

Based on the ITS+nrLSU+*TEF*1 topology (Fig. 2), *Basidiodendron fragilissimum* is positioned as sister to *B. yunnanense*. However, *B. yunnanense* can be distinguished from *B. fragilissimum* by its reticulate to porulose, greyish-blue hymenial surface (Duan and Zhao 2022). Morphologically, *B. fragilissimum* resembles *B. inconspicuum* and *B. iniquum* R.L.M. Alvarenga & K.H. Larss. in having a smooth hymenial surface. However, *B. inconspicuum* is distinct from *B. fragilissimum* by its whitish or greyish hymenial surface and minutely warted, wider basidiospores (5–6.2 × 5.2–6.5 µm vs. 4.5–5.5 × 4–5 µm; Spirin et al. 2021). *Basidiodendron iniquum* can be separated from *B. fragilissimum* by its waxy basidiomata and pale cream-colored or greyish hymenial surface (Spirin et al. 2020).

#### Basidiodendron odontoideum

Taxon classificationFungiAuricularialesBasidiomycota

Q. Yuan & C.L. Zhao sp. nov.

39F0C704-04B9-54F6-B54E-D59990DC1DAA

864143

Figs 15, 16, 17

##### Diagnosis.

*Basidiodendron odontoideum* differs from other species by its soft membranaceous basidiomata, odontioid hymenial surface, short clavate cystidia, and globose to subglobose, smooth basidiospores.

**Figure 15.**
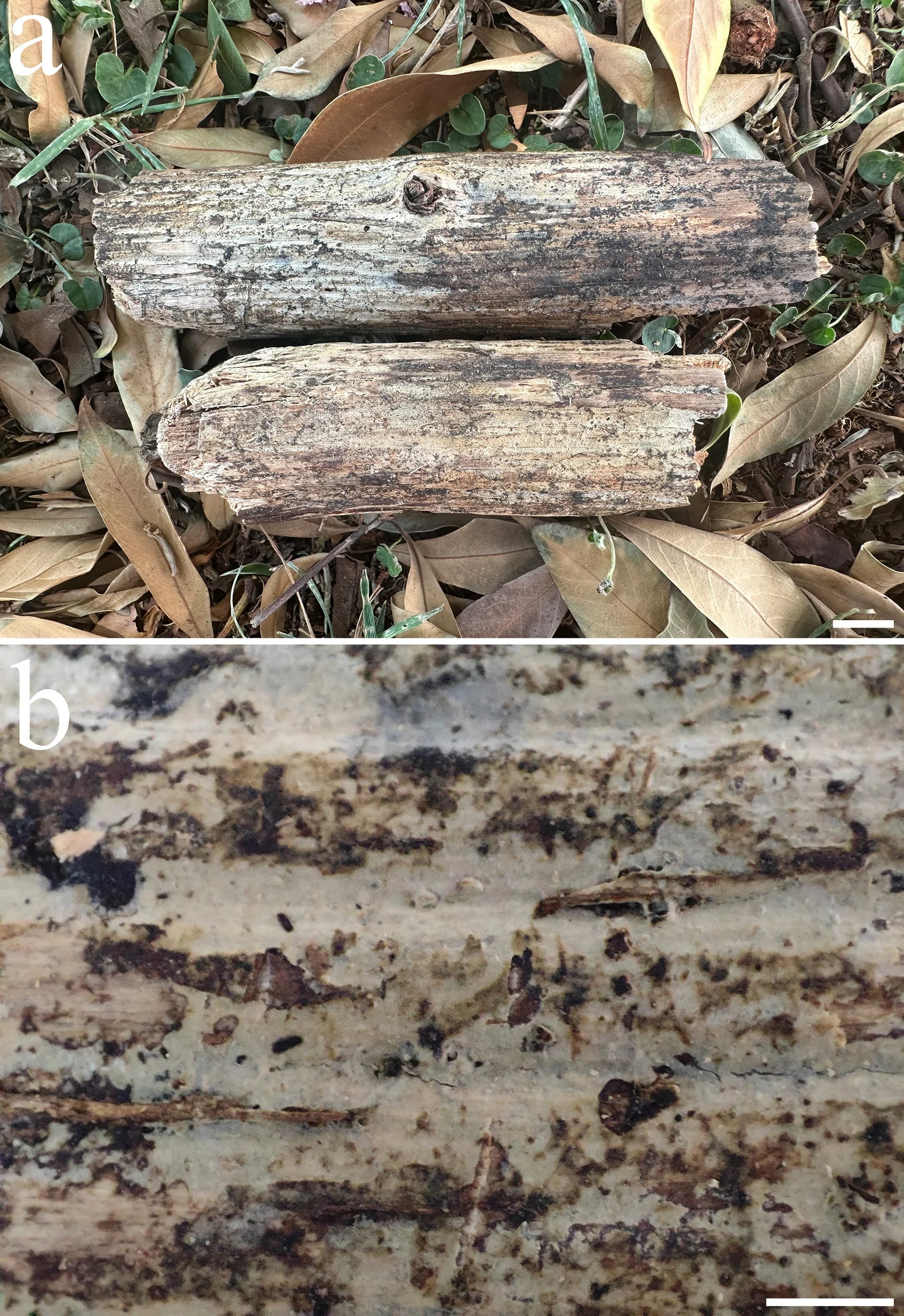
Basidiomata of *Basidiodendron odontoideum* (holotype CLZhao 32441). Scale bars: 1 cm (**a**); 2 mm (**b**).

**Figure 16.**
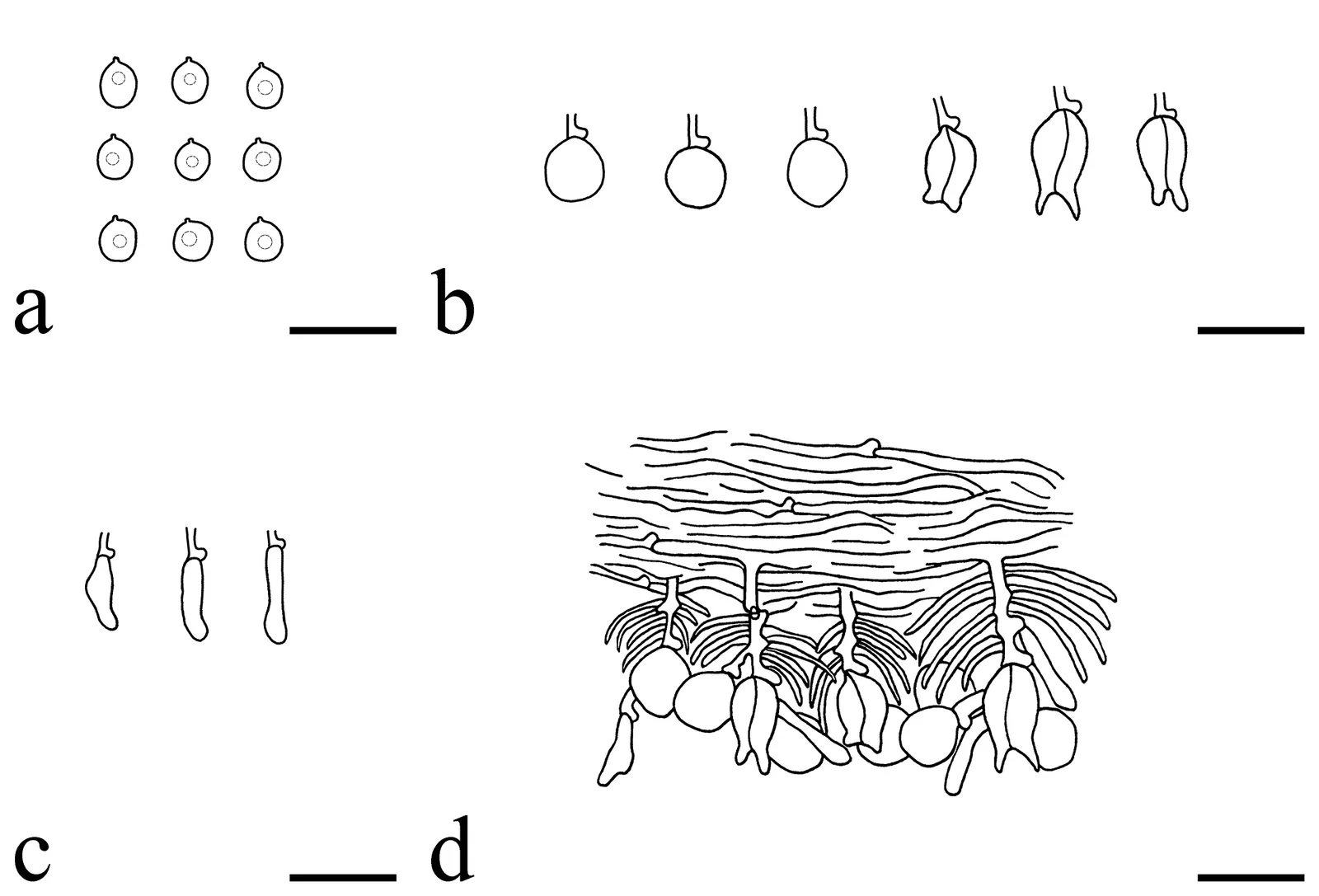
Microscopic structures of *Basidiodendron odontoideum* (holotype CLZhao 32441). **a** Basidiospores; **b** basidia and basidioles; **c** leptocystidia; **d** part of a vertical section of the hymenium. Scale bars: 10 µm (**a–d**).

**Figure 17.**
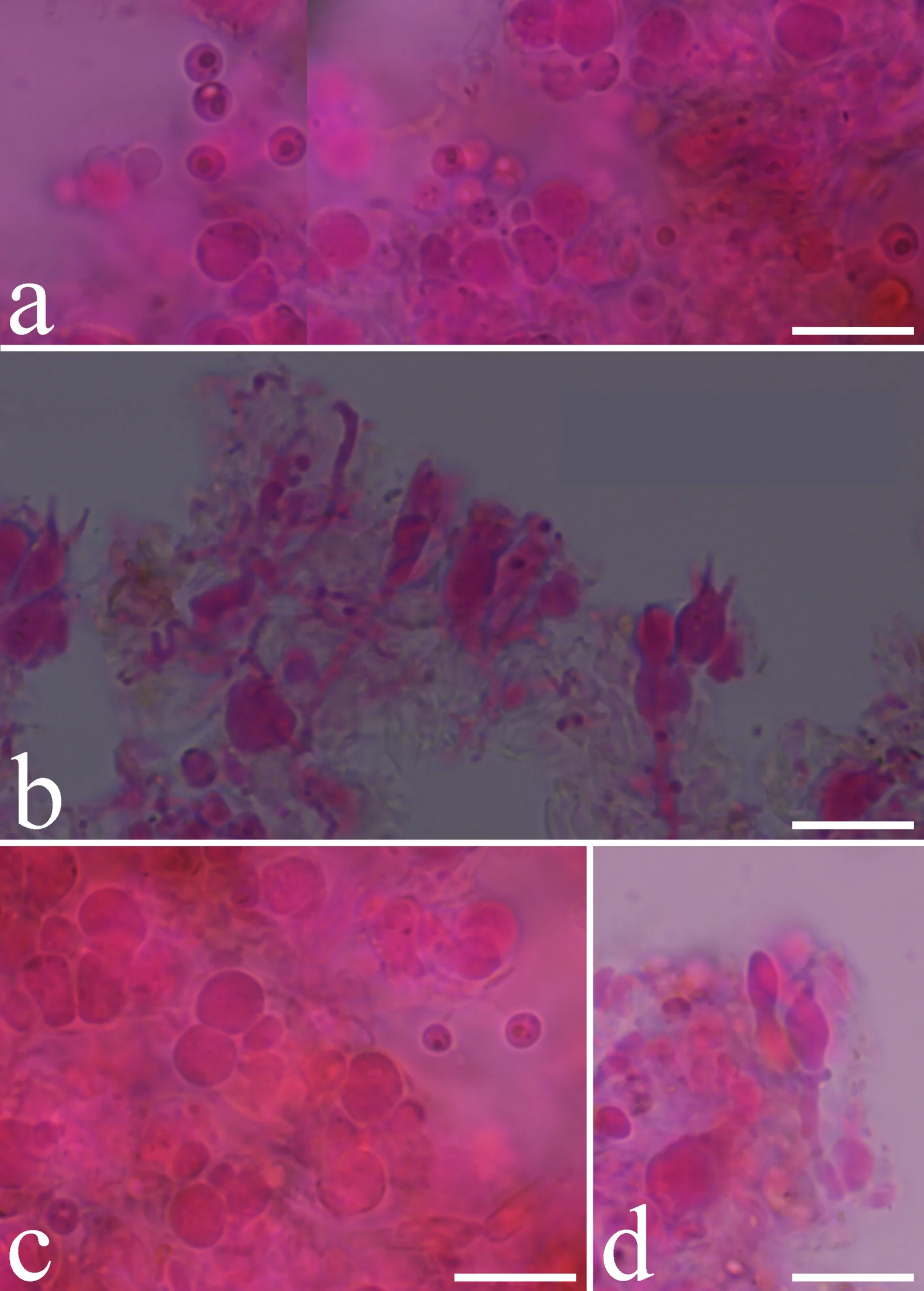
Sections of the hymenium of *Basidiodendron odontoideum* (holotype CLZhao 32441). **a** Basidiospores; **b** basidia; **c** basidioles; **d** leptocystidia. Scale bars: 10 µm (**a–d**).

##### Etymology.

*Odontoideum* (Lat.). Referring to the odontioid hymenophore of the type specimen.

##### Type.

CHINA • Yunnan Province, Zhaotong, Wumeng Mountain National Nature Reserve, 27°58'N, 104°15'E, elevation 2,154 m asl., on fallen angiosperm branch, leg. C.L. Zhao, 28 Aug. 2023, CLZhao 32441 (SWFC 00032441, holotype).

##### Description.

***Basidiomata*** annual, resupinate, closely adnate, soft membranaceous, without odor or taste when fresh, up to 11 cm long, 8 cm wide, and up to 50 µm thick. ***Hymenial surface*** odontioid, slightly cream when fresh, turning to cream upon drying. ***Sterile margin*** narrow, white, up to 1 mm. ***Hyphal system*** monomitic; generative hyphae with clamp connections, colorless, thin-walled, smooth, interwoven, 1–2 µm in diameter, IKI–, CB–; tissues unchanged in KOH. ***Leptocystidia*** short clavate, colorless, thin-walled, smooth, 6.5–8.5 × 2–2.5 µm. ***Hyphidia*** absent. ***Basidia*** ellipsoid to ovoid, longitudinally septate, two- to four-celled, 6.5–8.5 × 4.5–5 µm, involucres well developed, often totally covering basidial cells. ***Basidioles*** dominant, similar to basidia in shape, but slightly smaller. ***Basidiospores*** globose to subglobose, colorless, thin-walled, smooth, usually with one oil drop, IKI–, CB–, 3–3.5 × 2.5–3.5 µm, *L* = 3.33 µm, *W* = 3.05 µm, *Q* = 1.09–1.11 (*n* = 60/2).

##### Additional specimen examined (Paratype).

CHINA • Yunnan Province, Zhaotong, Wumeng Mountain National Nature Reserve, 28°02'N, 104°18'E, elevation 2,260 m asl., on fallen angiosperm branch, leg. C.L. Zhao, 28 Aug. 2023, CLZhao 50721 (SWFC 00050721).

##### Notes.

*Basidiodendron odontoideum* forms a single lineage (100% ML, 1.00 BPP) and resolves in a sister relationship to *B. parile* in the phylogenetic inference (Fig. 2). Key characters distinguishing *B. parile* from *B. odontoideum* are its waxy basidiomata with a smooth hymenial surface and larger basidiospores (4.7–5.9 × 4.1–5.6 µm vs. 3–3.5 × 2.5–3.5 µm; Spirin et al. 2020). Morphologically, *B. odontoideum* resembles *B. alni*, *B. mexicanum* Spirin & Malysheva, and *B. microsporum* Spirin, Malysheva & Kotiranta in having globose to subglobose basidiospores. However, *B. alni* can be separated from *B. odontoideum* by its smooth hymenial surface and larger basidiospores (4.1–5 × 4.2–5 µm vs. 3–3.5 × 2.5–3.5 µm; Spirin et al. 2020). *Basidiodendron mexicanum* is distinct from *B. odontoideum* by its pruinose basidiomata, smooth hymenial surface, and larger basidiospores (5.9–7.3 × 6.1–7.4 µm vs. 3–3.5 × 2.5–3.5 µm; Spirin et al. 2021). *Basidiodendron microsporum* differs from *B. odontoideum* by its pruinose-reticulate basidiomata and smooth hymenial surface (Spirin et al. 2020).

#### Basidiodendron rigidum

Taxon classificationFungiAuricularialesBasidiomycota

Q. Yuan & C.L. Zhao sp. nov.

B5EF6FAA-479B-5A16-8671-6315B8974848

864145

Figs 18, 19, 20

##### Diagnosis.

*Basidiodendron rigidum* differs from other species by its hard coriaceous basidiomata, brown to grayish brown hymenial surface and subcylindrical to subclavate cystidia.

**Figure 18.**
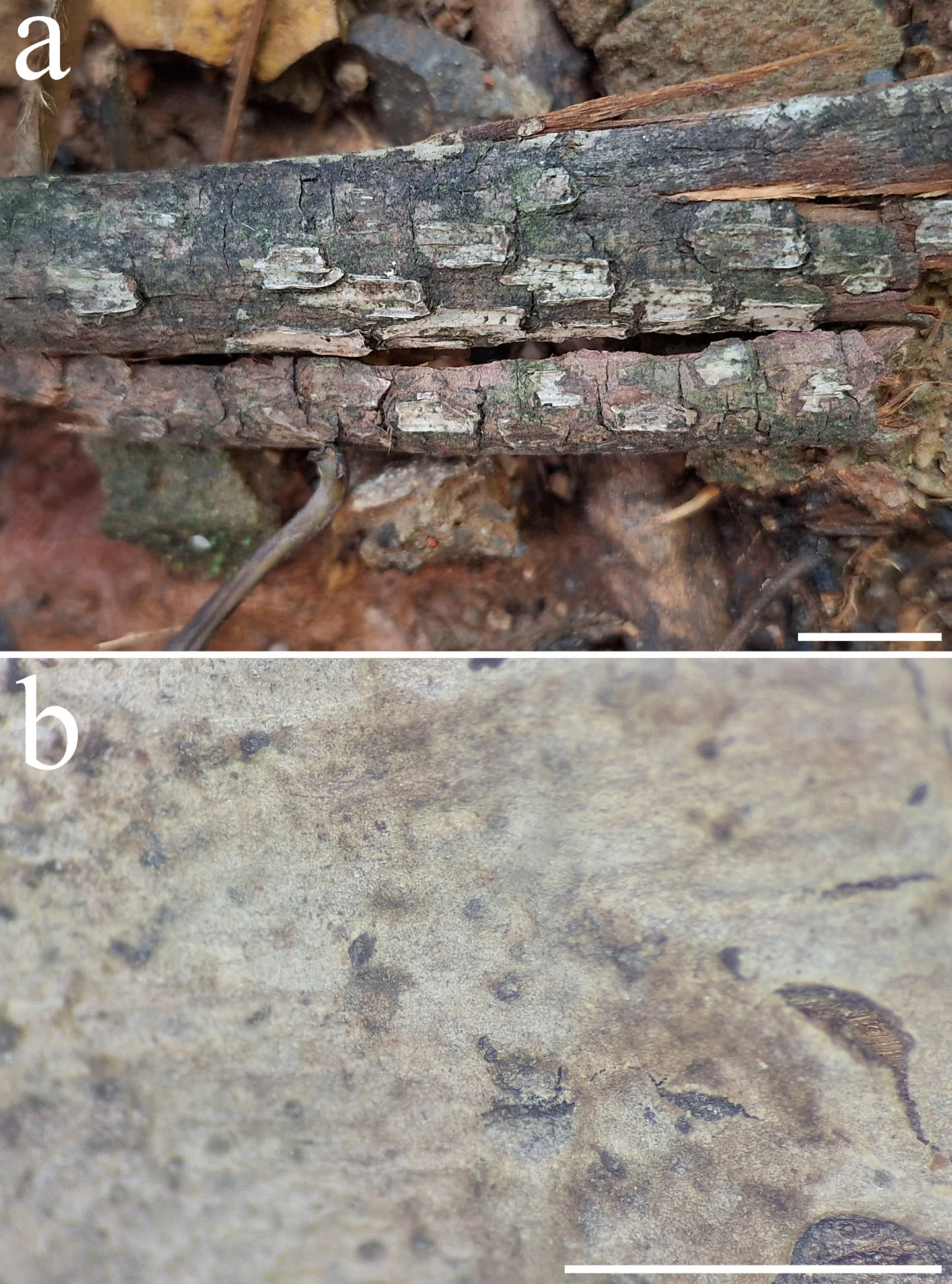
Basidiomata of *Basidiodendron rigidum* (holotype CLZhao 31785). Scale bars: 1 cm (**a**); 2 mm (**b**).

**Figure 19.**
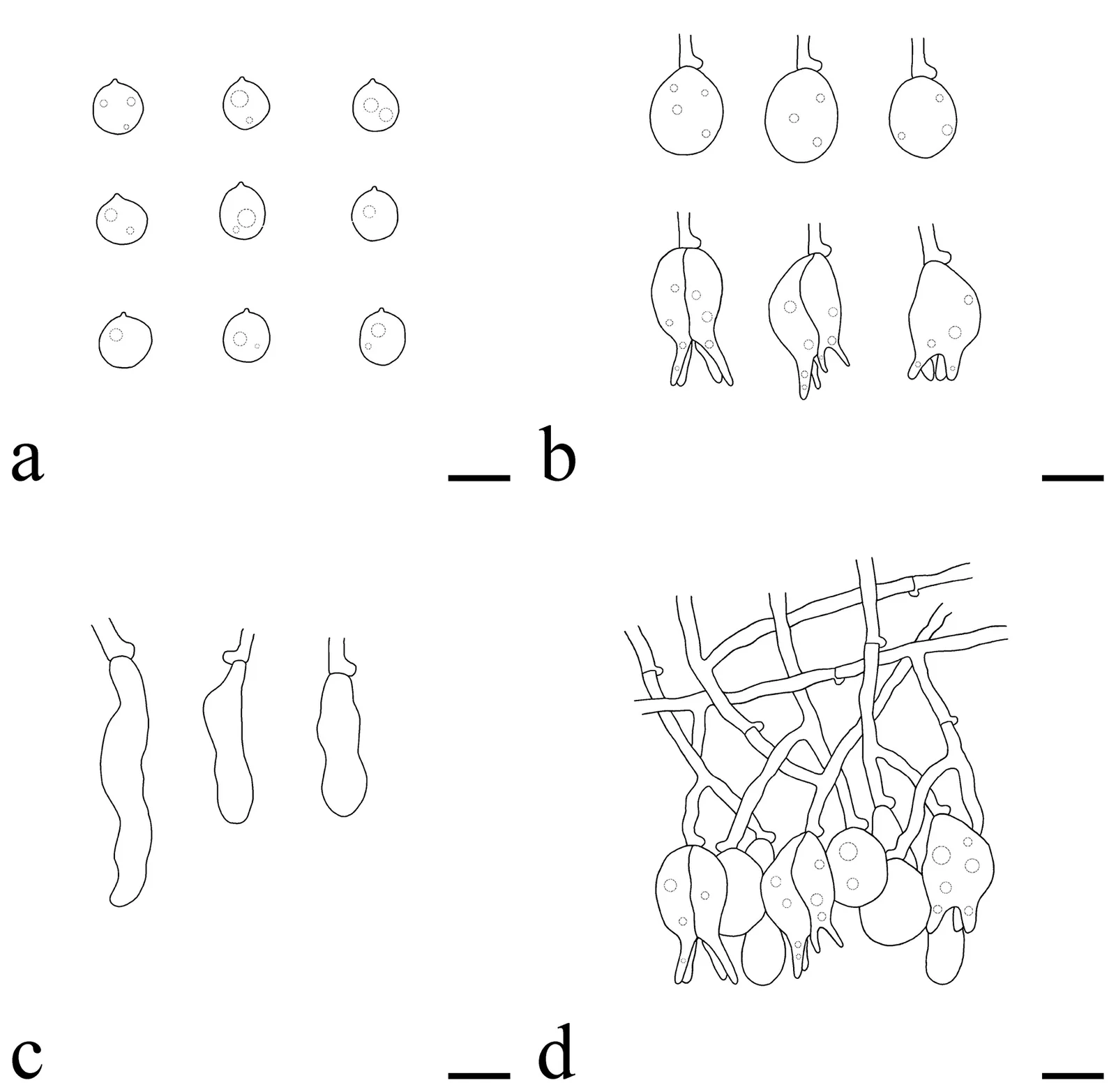
Microscopic structures of *Basidiodendron rigidum* (holotype CLZhao 31785). **a** Basidiospores; **b** basidia and basidioles; **c** leptocystidia; **d** part of a vertical section of the hymenium. Scale bars: 10 µm (**a–d**).

**Figure 20.**
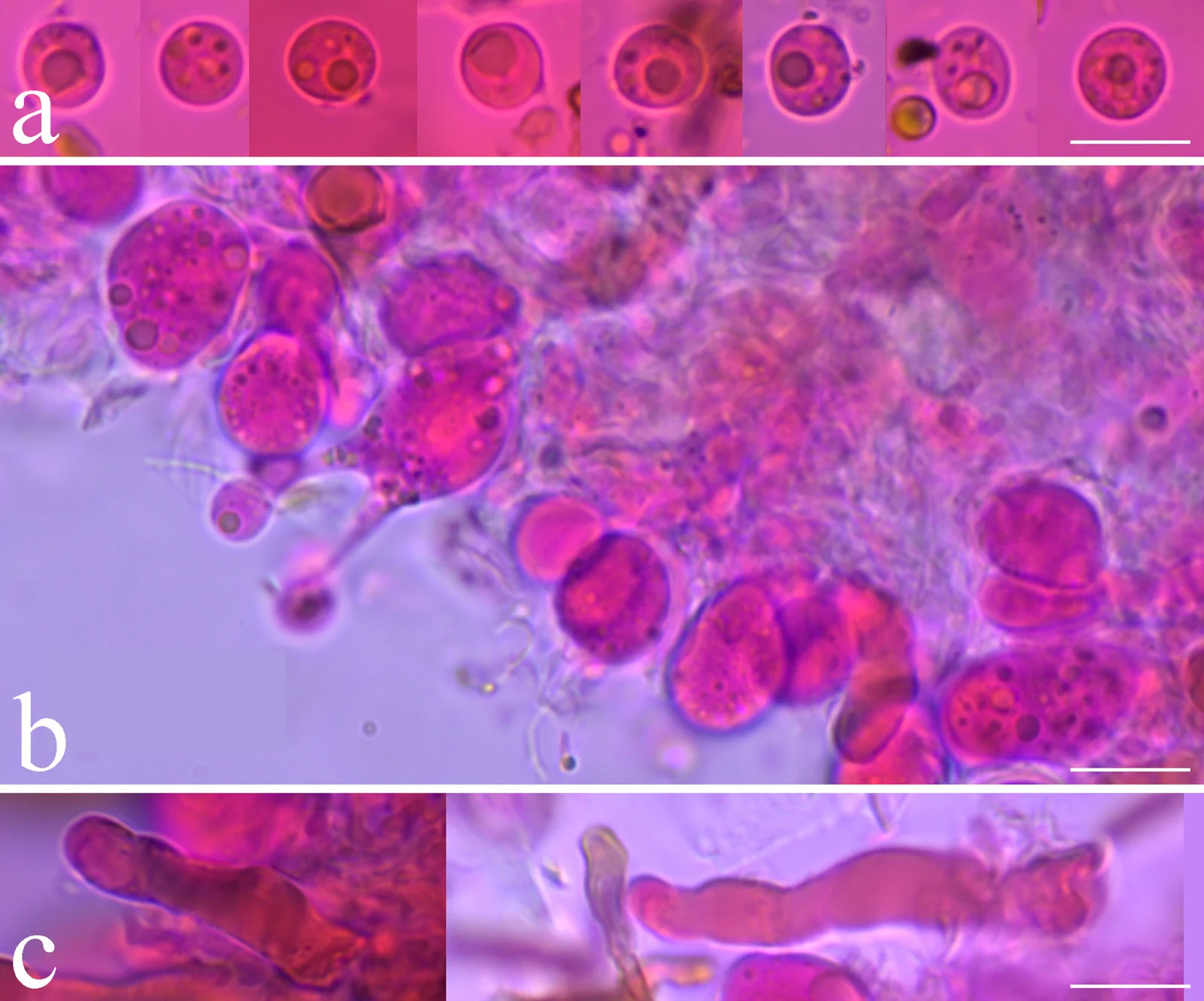
Sections of the hymenium of *Basidiodendron rigidum* (holotype CLZhao 31785). **a** Basidiospores; **b** basidia and basidioles; **c** leptocystidia. Scale bars: 10 µm (**a–c**).

##### Etymology.

*Rigidum* (Lat.). Referring to the rigid basidiomata of the type specimen.

##### Type.

CHINA • Yunnan Province, Zhaotong, Wumengshan National Nature Reserve, 28°03'N, 104°20'E, elevation 1,500 m asl., on fallen angiosperm branches, leg. C.L. Zhao, 26 Aug. 2023, CLZhao 31785 (SWFC 00031785, holotype).

##### Description.

***Basidiomata*** annual, resupinate, adnate, soft coriaceous, very hard to separate from substrate, without odor or taste when fresh, becoming hard coriaceous upon drying, up to 6.5 cm long, 0.5 cm wide, and 50 µm thick. ***Hymenial surface*** smooth, brown when fresh, turning to brown to grayish brown upon drying. ***Sterile margin*** narrow, grayish brown, up to 1 mm. ***Hyphal system*** monomitic; generative hyphae with clamp connections, colorless, thin-walled, smooth, interwoven, 2–2.5 µm in diameter, IKI–, CB–; tissues unchanged in KOH. ***Leptocystidia*** subcylindrical to subclavate, thin-walled, occasionally sinuous in the basal position, 21.5–51.5 × 6–6.5 µm. ***Hyphidia*** absent. ***Basidia*** ellipsoid to ovoid, longitudinally septate, four-celled, 13–19.5 × 10–11 µm, involucres indistinct. ***Basidioles*** dominant, similar to basidia in shape, but slightly smaller. ***Basidiospores*** globose, colorless, thin-walled, smooth, usually with oil drops, IKI–, CB–, (6.5–)7–8 × 6–7.5 µm, *L* = 7.49 µm, *W* = 6.83 µm, *Q* = 1.09–1.11 (*n* = 60/2).

##### Additional specimen examined (Paratype).

CHINA • Yunnan Province, Zhaotong, Wumengshan National Nature Reserve, 28°17'N, 104°45'E, elevation 2,454 m asl., on fallen angiosperm branches, leg. C.L. Zhao, 19 Sep. 2023, CLZhao 33334 (SWFC 00033334).

##### Notes.

Based on the ITS+nrLSU+*TEF*1 topology (Fig. 2), *Basidiodendron rigidum* (CLZhao 31785, CLZhao 33334) forms a single lineage (95% ML, 0.96 BPP) and clusters with *B. iniquum*, *B. pelinum*, and *B. salebrosum*. However, *B. iniquum* can be delimited from *B. rigidum* by its waxy basidiomata with a pale cream or greyish hymenial surface and smaller basidiospores (4.9–6.2 × 4.1–5.2 µm vs. 7–8 × 6–7.5 µm; Spirin et al. 2020). *Basidiodendron pelinum* can be delimited from *B. rigidum* by its pruinose basidiomata with a tuberculate hymenial surface and smaller basidiospores (4.9–7.1 × 3.9–5.2 µm vs. 7–8 × 6–7.5 µm; Spirin et al. 2020). *Basidiodendron salebrosum* differs from *B. rigidum* by its waxy or arid basidiomata with a greyish to pale ochraceous hymenial surface and narrower basidiospores (5.7–7.6 × 4.8–6.1 µm vs. 7–8 × 6–7.5 µm; Spirin et al. 2020). Morphologically, *B. rigidum* resembles *B. robenae* Spirin & Miettinen and *B. spiculosum* in having a smooth hymenial surface. However, *B. robenae* is distinct from *B. rigidum* by its pale ochraceous to greyish hymenial surface and shorter basidiospores (5.1–6.4 × 5.2–6.9 µm vs. 7–8 × 6–7.5 µm; Spirin et al. 2021). *Basidiodendron spiculosum* can be separated from *B. rigidum* by its pruinose-reticulate basidiomata and spiny basidiospores (Spirin et al. 2021).

#### Basidiodendron ruiliense

Taxon classificationFungiAuricularialesBasidiomycota

Q. Yuan & C.L. Zhao sp. nov.

FEF07298-3A1A-5AE9-BD8F-60ABF037592B

864146

Figs 21, 22, 23

##### Diagnosis.

*Basidiodendron ruiliense* differs from other species by its farinaceous basidiomata and globose to subglobose basidiospores.

**Figure 21.**
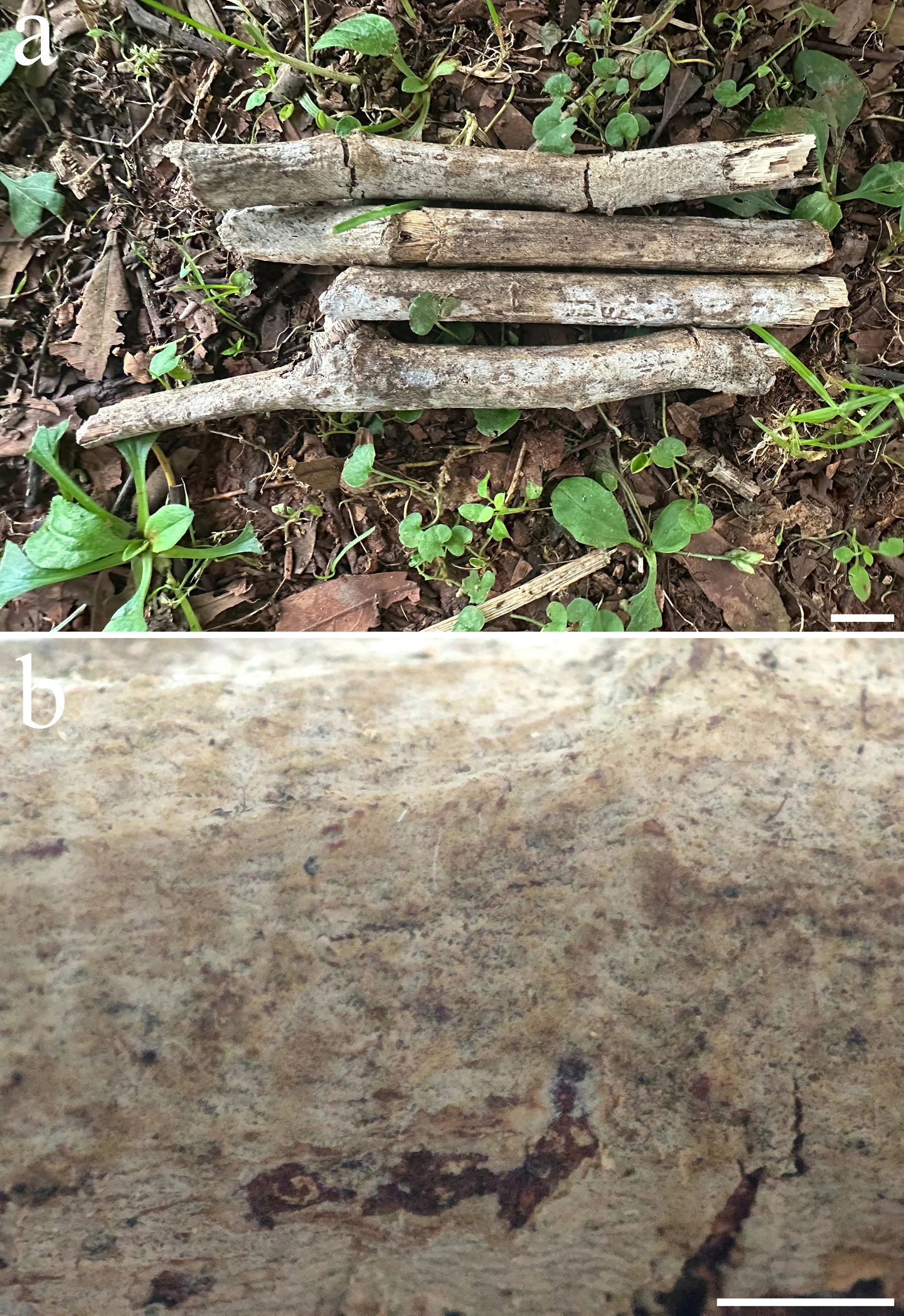
Basidiomata of *Basidiodendron ruiliense* (holotype CLZhao 41383). Scale bars: 1 cm (**a**); 2 mm (**b**).

**Figure 22.**
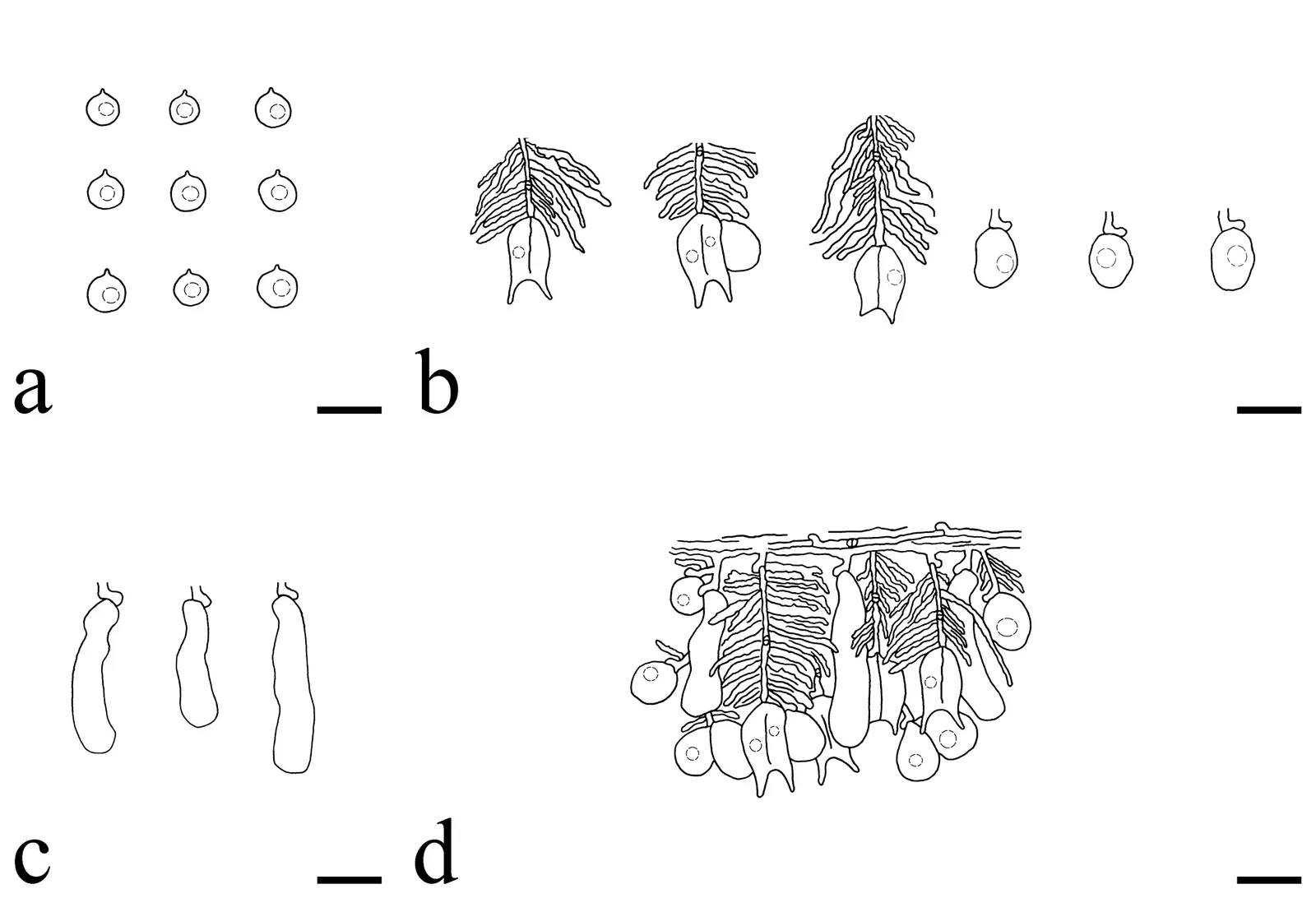
Microscopic structures of *Basidiodendron ruiliense* (holotype CLZhao 41383). **a** Basidiospores; **b** basidia and basidioles; **c** leptocystidia; **d** part of a vertical section of the hymenium. Scale bars: 10 µm (**a–d**).

**Figure 23.**
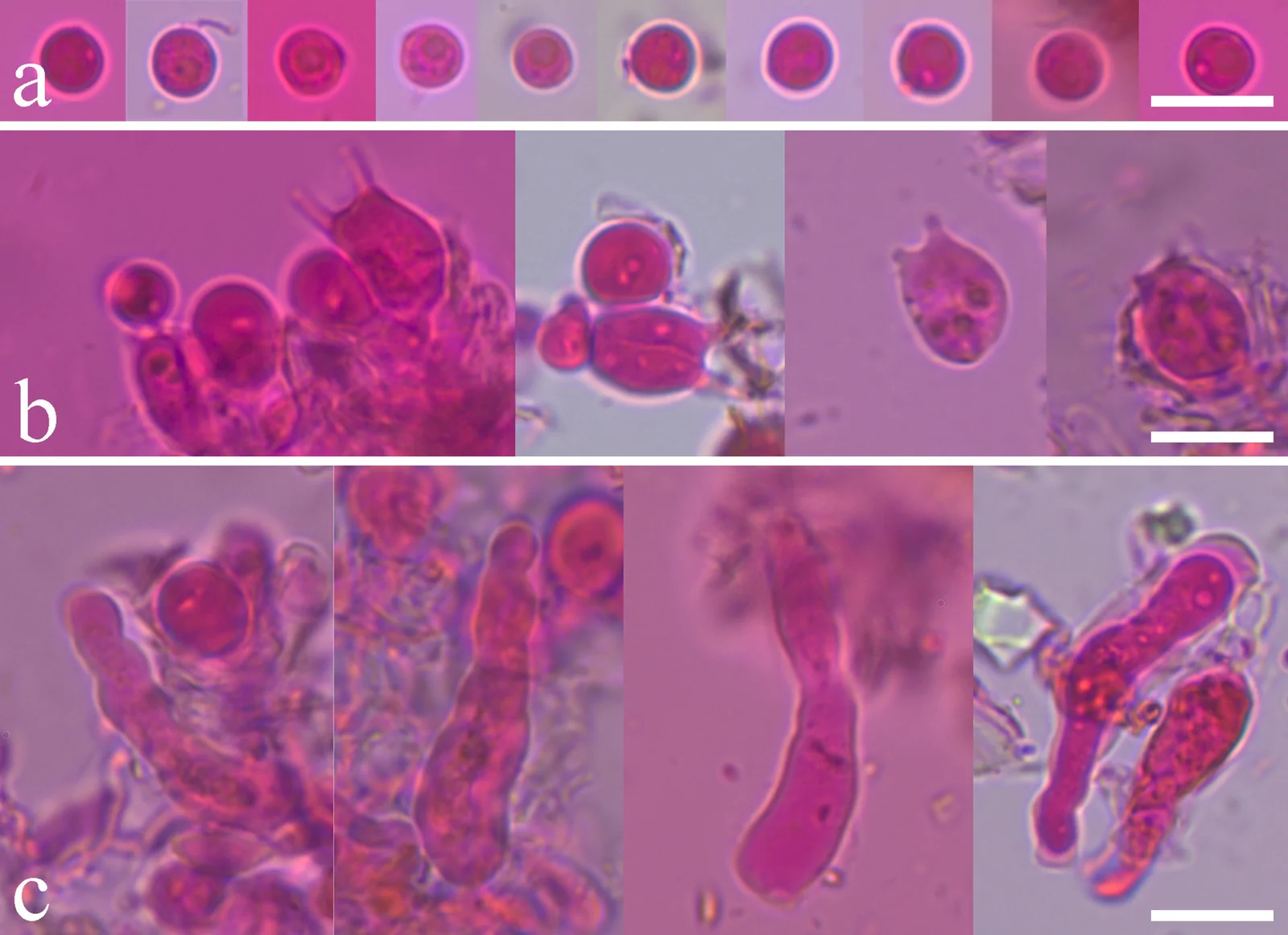
Sections of the hymenium of *Basidiodendron ruiliense* (holotype CLZhao 41383). **a** Basidiospores; **b** basidia and basidioles; **c** leptocystidia. Scale bars: 10 µm (**a–c**).

##### Etymology.

*Ruiliense* (Lat.). Referring to the type locality, Ruili, Yunnan Province, China.

##### Type.

CHINA • Yunnan Province, Dehong, Ruili City, Tongbiguan Provincial Nature Reserve, 24°11'N, 97°31'E, elevation 1,945 m asl., on fallen angiosperm branch, leg. C.L. Zhao, 21 Nov. 2024, CLZhao 41383 (SWFC 00041383, holotype).

##### Description.

***Basidiomata*** annual, resupinate, closely adnate, farinaceous, without odor or taste when fresh, up to 12.5 cm long, 1.5 cm wide, and 100 µm thick. ***Hymenial surface*** smooth, white to cream when fresh, turning to cream upon drying. ***Sterile margin*** narrow, white, up to 1 mm. ***Hyphal system*** monomitic; generative hyphae with clamp connections, colorless, thin-walled, smooth, interwoven, 1.5–2.5 µm in diameter, IKI–, CB–; tissues unchanged in KOH. ***Leptocystidia*** subclavate, colorless, thin-walled, smooth, 18.5–27.5 × 4–6.5 µm. ***Hyphidia*** absent. ***Basidia*** ellipsoid to ovoid, longitudinally septate, two- to four-celled, 9.5–11.5 × 6–7.5 µm, involucres well developed, often totally covering basidial cells. ***Basidioles*** dominant, similar to basidia in shape, but slightly smaller. ***Basidiospores*** globose to subglobose, colorless, thin-walled, smooth, usually with one oil drop, IKI–, CB–, (4–)4.5–6 × 4–5.5(–5.5) µm, *L* = 5.13 µm, *W* = 4.98 µm, *Q* = 1.01–1.03 (*n* = 60/2).

##### Additional specimen examined (Paratype).

CHINA • Yunnan Province, Dehong, Mang City, Mengga Town, Tongbiguan Provincial Nature Reserve, 24°21'N, 98°05'E, elevation 2,107 m asl., on fallen angiosperm branch, leg. C.L. Zhao, 29 Jul. 2024, CLZhao 36504 (SWFC 00036504).

##### Notes.

*Basidiodendron ruiliense* formed a well-supported single lineage (100% ML, 1.00 BPP) and grouped with *B. fragilissimum* and *B. yunnanense* in the phylogenetic inference using ITS+nrLSU+*TEF*1 data (Fig. 2). However, *B. fragilissimum* can be delimited from *B. ruiliense* by its membranaceous basidiomata with a slightly olivaceous to slightly brown hymenial surface and shorter subclavate cystidia (9–15.5 × 2.5–5 µm vs. 18.5–27.5 × 4–6.5 µm). *Basidiodendron yunnanense* differs from *B. ruiliense* by its grayish-blue, reticulate to porulose hymenial surface (Duan and Zhao 2022). Morphologically, *B. ruiliense* resembles *B. alni* and *B. mexicanum* in having a smooth hymenial surface. However, *B. alni* differs from *B. ruiliense* by its waxy or partly gelatinized basidiomata. *Basidiodendron mexicanum* can be separated from *B. ruiliense* by its grayish to pale ochraceous hymenial surface and spiny, larger basidiospores (5.9–7.3 × 6.1–7.4 µm vs. 4.5–6 × 4–5.5 µm; Spirin et al. 2021).

#### Basidiodendron tenissimum

Taxon classificationFungiAuricularialesBasidiomycota

Q. Yuan & C.L. Zhao sp. nov.

D2AB2096-D0B1-5C74-BE65-F636439E1A52

864148

Figs 24, 25, 26

##### Diagnosis.

*Basidiodendron tenissimum* differs from other species by its buff to cinnamon-buff, smooth hymenial surface, subcylindrical to subclavate with an obtuse apex, occasionally sinuous in the basal position cystidia, and ellipsoid to globose basidiospores.

**Figure 24.**
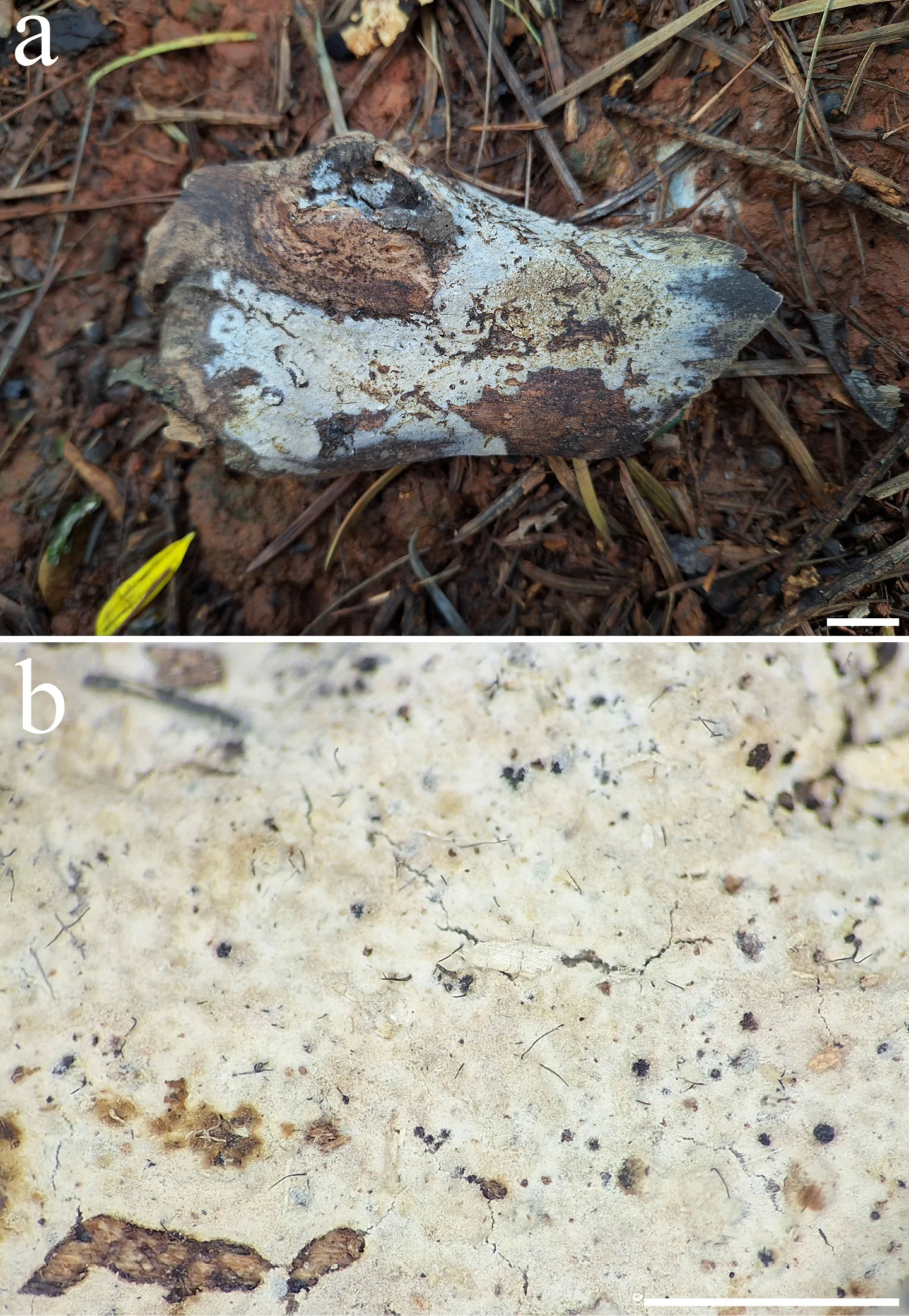
Basidiomata of *Basidiodendron tenissimum* (holotype CLZhao 30794). Scale bars: 1 cm (**a**); 2 mm (**b**).

**Figure 25.**
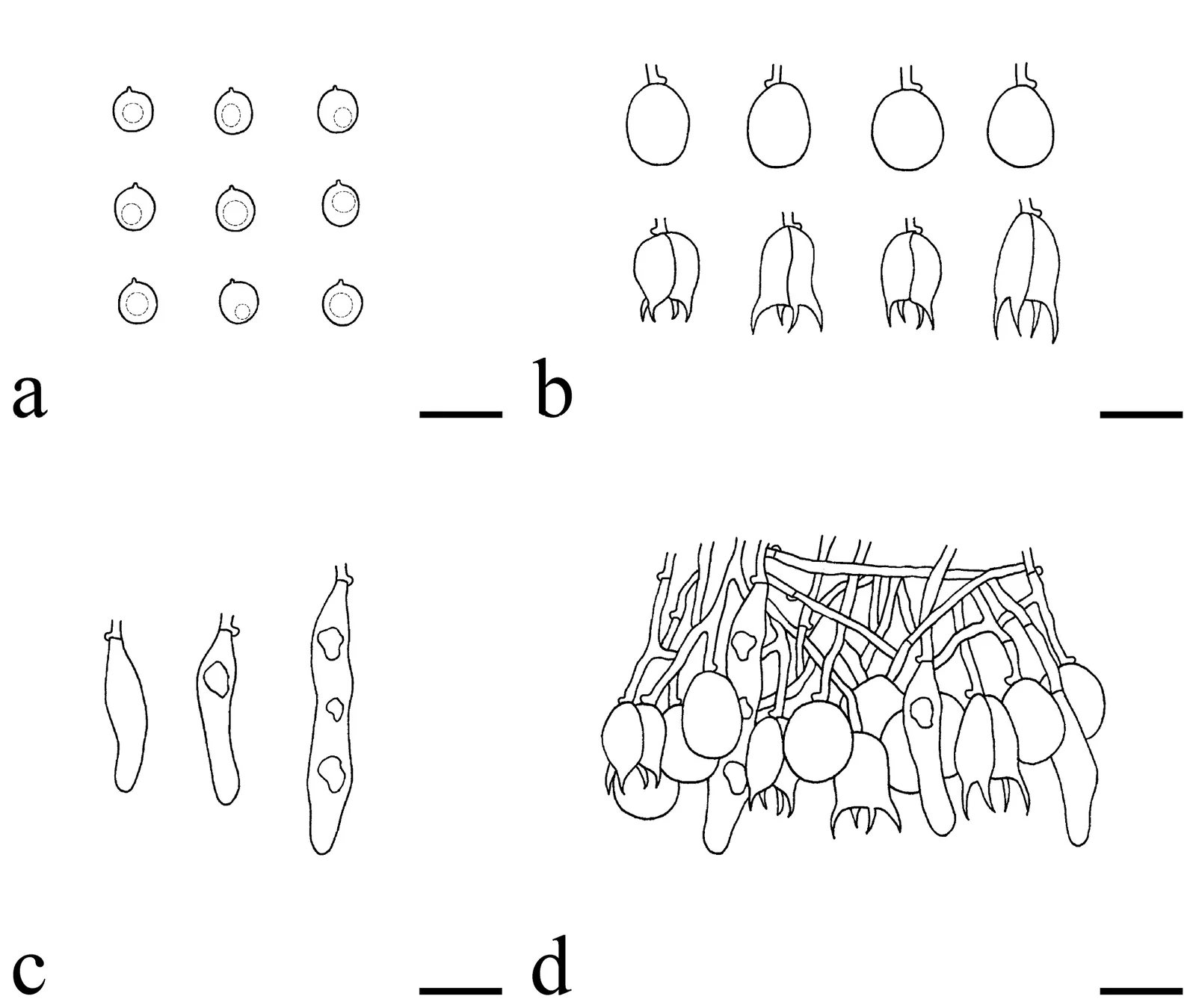
Microscopic structures of *Basidiodendron tenissimum* (holotype CLZhao 30794). **a** Basidiospores; **b** basidia and basidioles; **c** gloeocystidia; **d** part of a vertical section of the hymenium. Scale bars: 10 µm (**a–d**).

**Figure 26.**
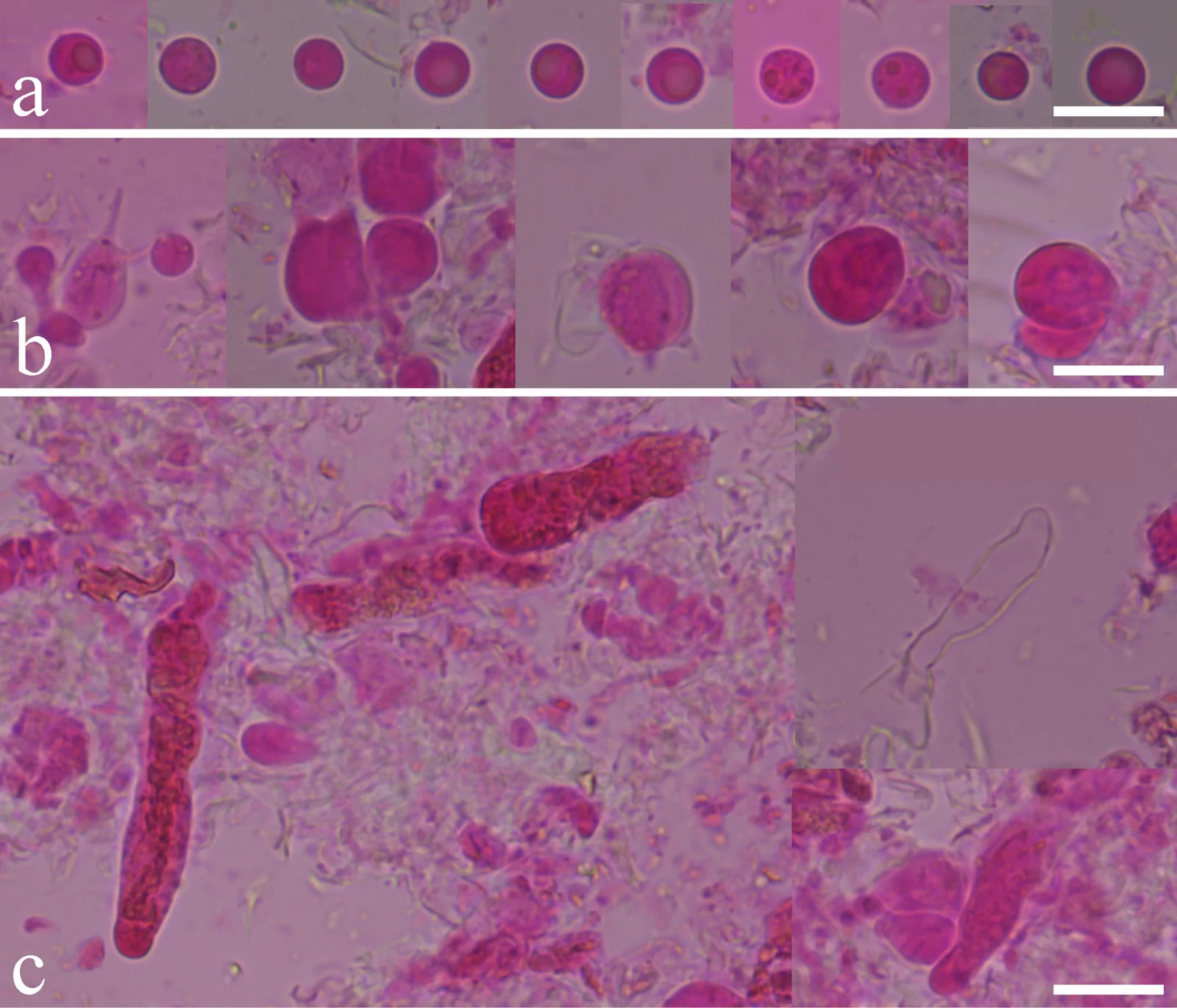
Sections of the hymenium of *Basidiodendron tenissimum* (holotype CLZhao 30794). **a** Basidiospores; **b** basidia and basidioles; **c** gloeocystidia. Scale bars: 10 µm (**a–c**).

##### Etymology.

*Tenissimum* (Lat.). Referring to the type specimens having tenuous basidiocarps.

##### Type.

CHINA • Yunnan Province, Dehong, Yingjiang County, Tongbiguan Provincial Nature Reserve, 25°50'N, 97°36'E, elevation 1,000 m asl., on fallen angiosperm branch, leg. C.L. Zhao, 21 Jul. 2023, CLZhao 30794 (SWFC 00030794, holotype).

##### Description.

***Basidiomata*** annual, resupinate, closely adnate, coriaceous, without odor or taste when fresh, up to 8 cm long, 4 cm wide, and 50–100 µm thick. ***Hymenial surface*** smooth, cream to buff when fresh, buff to cinnamon-buff upon drying, cracking. ***Sterile margin*** narrow, white to cream, up to 1 mm. ***Hyphal system*** monomitic; generative hyphae with clamp connections, colorless, thin-walled, smooth, rarely branched, interwoven, 1.5–2 µm in diameter, IKI–, CB–; tissues unchanged in KOH. ***Gloeocystidia*** numerous, thin-walled, subcylindrical to subclavate with an obtuse apex, occasionally sinuous in the basal position, 17–34 × 3.5–6 µm. ***Hyphidia*** absent. ***Basidia*** narrowly ovoid to ellipsoid, longitudinally septate, four-celled, 8–10 × 5.5–9 µm, involucres absent. ***Basidioles*** dominant, similar to basidia in shape, but slightly smaller. ***Basidiospores*** ellipsoid to globose, colourless, thin-walled, smooth, usually with one oil drop, IKI–, CB–, 4–5(–5.5) × 4–5 µm, *L* = 4.79 µm, *W* = 4.64 µm, *Q* = 1.02–1.04 (*n* = 60/2).

##### Additional specimen examined (Paratype).

CHINA • Yunnan Province, Dehong, Yingjiang County, Tongbiguan Provincial Nature Reserve, 24°45'N, 97°35'E, elevation 1,760 m asl., on fallen angiosperm branch, leg. C.L. Zhao, 21 Jul. 2023, CLZhao 30844 (SWFC 00030844).

##### Notes.

The combined ITS+nrLSU+*TEF*1 phylogenetic analysis revealed that *Basidiodendron tenissimum* formed a single lineage (100% ML, 1.00 BPP) and grouped with two taxa, *B. dehongense* and *B. luteogriseum* (Fig. 2). However, *B. dehongense* can be delimited from *B. tenissimum* by its membranaceous basidiomata with a slightly olivaceous to buff hymenial surface and longer basidiospores (5.5–7.5 × 4.5–6.5 µm vs. 4–5 × 4–5 µm). *Basidiodendron luteogriseum* differs from *B. tenissimum* by its pruinose hymenial surface (Spirin et al. 2020). Morphologically, *B. tenissimum* resembles *B. inconspicuum* and *B. iniquum* in having a smooth hymenial surface. However, *B. inconspicuum* is distinct from *B. tenissimum* by its pruinose-reticulate hymenial surface and larger basidiospores (5–6.2 × 5.2–6.5 µm vs. 4–5 × 4–5 µm; Spirin et al. 2021). *Basidiodendron iniquum* can be separated from *B. tenissimum* by its pale cream-colored or greyish hymenial surface and longer basidiospores (4.9–6.2 × 4.1–5.2 µm vs. 4–5 × 4–5 µm; Spirin et al. 2020).

#### Basidiodendron zhaotongense

Taxon classificationFungiAuricularialesBasidiomycota

Q. Yuan & C.L. Zhao sp. nov.

51D679D2-E80B-5E7A-86DB-A92DE803EB0D

864149

Figs 27, 28, 29

##### Diagnosis.

*Basidiodendron zhaotongense* differs from other species by its membranaceous basidiomata, reticulate hymenial surface.

**Figure 27.**
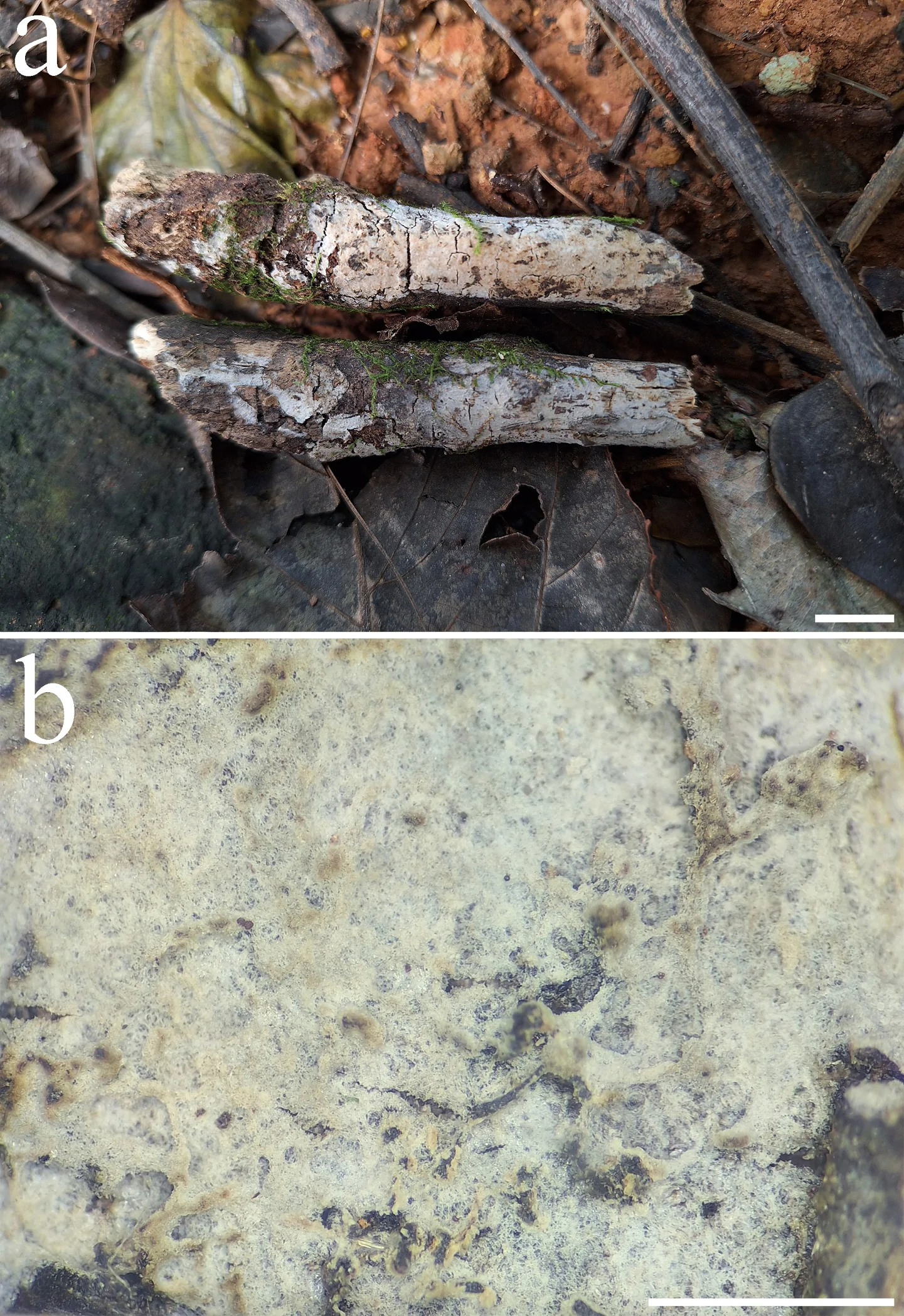
Basidiomata of *Basidiodendron zhaotongense* (holotype CLZhao 32672). Scale bars: 1 cm (**a**); 2 mm (**b**).

**Figure 28.**
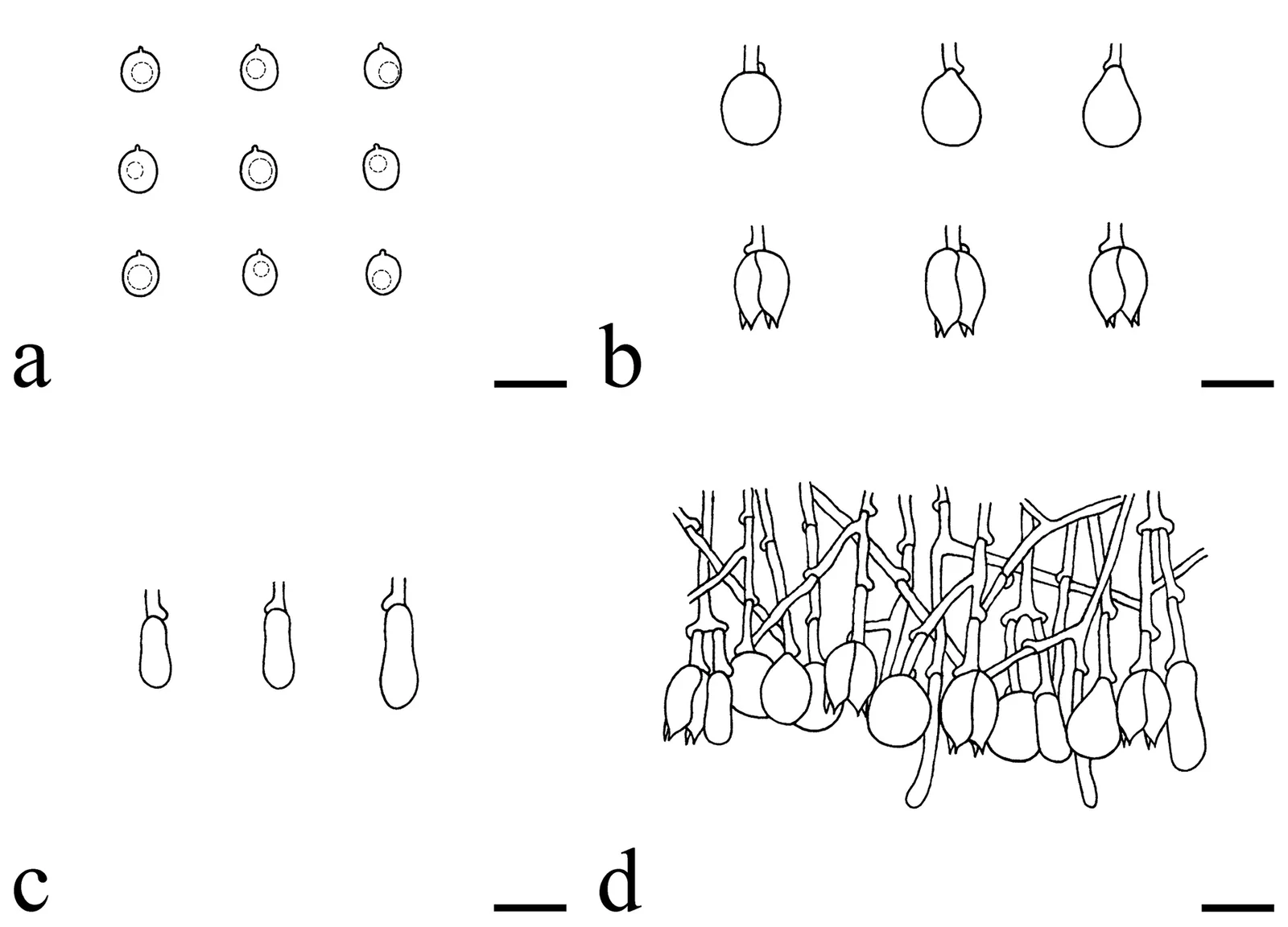
Microscopic structures of *Basidiodendron zhaotongense* (holotype CLZhao 32672). **a** Basidiospores; **b** basidia and basidioles; **c** leptocystidia; **d** part of a vertical section of the hymenium. Scale bars: 10 µm (**a–d**).

**Figure 29.**
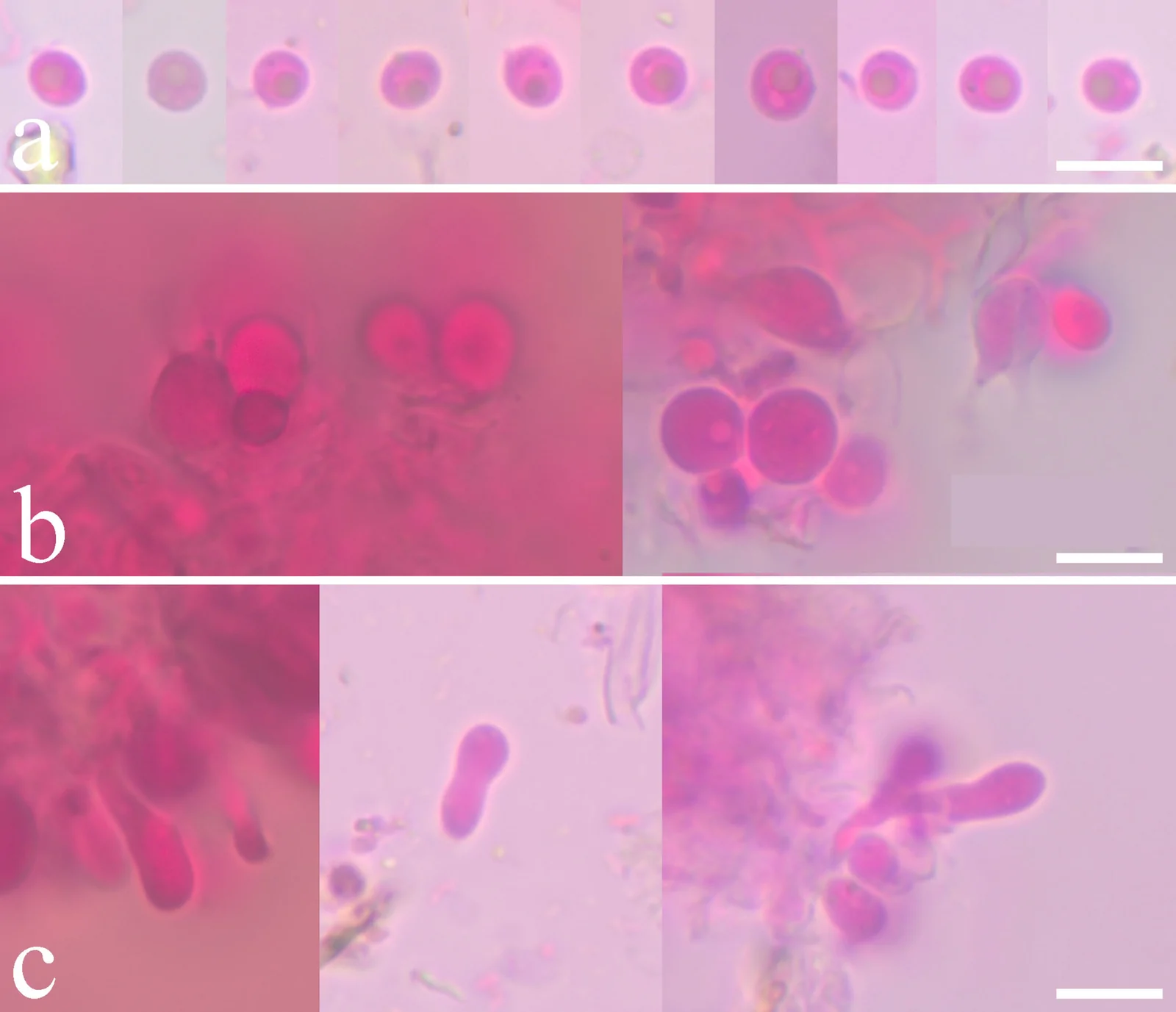
Sections of the hymenium of *Basidiodendron zhaotongense* (holotype CLZhao 32672). **a** Basidiospores; **b** basidia and basidioles; **c** leptocystidia. Scale bars: 10 µm (**a–c**).

##### Etymology.

*Zhaotongense* (Lat.). Referring to the type locality, Zhaotong, Yunnan Province, China.

##### Type.

CHINA • Yunnan Province, Zhaotong, Wumeng Mountain National Nature Reserve, 27°77'N, 104°29'E, elevation 2,900 m asl., on fallen angiosperm branch, leg. C.L. Zhao, 29 Aug. 2023, CLZhao 32672 (SWFC 00032672, holotype).

##### Description.

***Basidiomata*** annual, resupinate, closely adnate, membranaceous, without odor or taste when fresh, up to 5 cm long, 2 cm wide, and 150 µm thick. ***Hymenial surface*** reticulate, white to cream when fresh, turning to cream to slightly buff upon drying. ***Sterile margin*** narrow, white to cream, up to 1 mm. ***Hyphal system*** monomitic; generative hyphae with clamp connections, colorless, thin-walled, smooth, interwoven, 1.5–2.5 µm in diameter, IKI–, CB–; tissues unchanged in KOH. ***Leptocystidia*** subclavate, colorless, thin-walled, smooth, 9–14 × 4–4.5 µm. ***Hyphidia*** absent. ***Basidia*** ellipsoid to ovoid, longitudinally septate, four-celled, 8–10 × 6.5–8.5 µm, involucres absent. ***Basidioles*** dominant, similar to basidia in shape, but slightly smaller. ***Basidiospores*** ellipsoid to globose, colorless, thin-walled, smooth, usually with one or more oil drops, IKI–, CB–, 4–5(–5.5) × (3–)3.5–4.5(–5.5) µm, *L* = 4.63 µm, *W* = 3.89 µm, *Q* = 1.15–1.22 (*n* = 90/3).

##### Additional specimens examined (Paratype).

CHINA • Yunnan Province, Zhaotong, Wumeng Mountain National Nature Reserve, 28°11'N, 104°01'E, elevation 2,200 m asl., on fallen angiosperm branch, leg. C.L. Zhao, 28 Aug. 2023, CLZhao 32305 (SWFC 00032305); 19 Sep. 2023, CLZhao 33274 (SWFC 00033274).

##### Notes.

In the present study, the phylogram inferred from the ITS+nrLSU+*TEF*1 data showed that *B. zhaotongense* formed a single lineage (100% ML, 1.00 BPP) and grouped with *B. alni* and *B. eyrei* (Fig. 2). However, *B. alni* can be distinguished from *B. zhaotongense* by its waxy or partly gelatinized basidiomata with a pruinose hymenial surface and wider basidiospores (4.1–5 × 4.2–5 µm vs. 4–5 × 3.5–4.5 µm; Spirin et al. 2020). *Basidiodendron eyrei* differs from *B. zhaotongense* by its pruinose hymenial surface and wider basidiospores (3.8–5.2 × 4.2–5.4 µm vs. 4–5 × 3.5–4.5 µm; Spirin et al. 2020). Morphologically, *B. zhaotongense* resembles *B. spiculosum*, *B. trachysporum* (Bourdot & Galzin) Spirin, M. Weiß & Miettinen, and *B. walleynii* Spirin, Malysheva & Schoutt. in having ovoid basidia. However, *B. spiculosum* is distinct from *B. zhaotongense* by its pruinose-reticulate basidiomata and spiny, larger basidiospores (6.9–8.2 × 7.1–8.9 µm vs. 4–5 × 3.5–4.5 µm; Spirin et al. 2021). *Basidiodendron trachysporum* differs from *B. zhaotongense* by its pruinose basidiomata and warted, wider basidiospores (4.8–7.4 × 5–7.8 µm vs. 4–5 × 3.5–4.5 µm; Spirin et al. 2021).

## Discussion

The order *Auriculariales* currently accommodates two widely recognized families, *Auriculariaceae* and *Hyaloriaceae* (He et al. 2024). However, a substantial number of distinct genera, notably *Basidiodendron*, remain *incertae sedis* at the family level (Spirin et al. 2020, 2021). The present study incorporated nine newly described species of *Basidiodendron* from southwestern China into a comprehensive multigene dataset (ITS+nrLSU+*RPB*1+*RPB*2+*TEF*1). Although the five-gene phylogenetic analyses provide a highly resolved topological framework for these novel taxa within the core *Basidiodendron* clade, suprageneric reclassifications (e.g., establishing a new family) are not proposed at this stage. As indicated by recent studies and the phylogenetic evaluations conducted here, deep-node evolutionary relationships within *Auriculariales* remain partially unresolved because of missing data across available taxa and the absence of critical ultrastructural evidence, particularly regarding septal pore architecture (Spirin et al. 2020, 2021; Spirin et al. 2025). Therefore, a definitive family-level reassessment of the *Basidiodendron* lineage awaits future investigations with expanded, unbiased global sampling and ultrastructural data.

The generic boundaries and monophyly of *Basidiodendron* have historically been controversial. In comprehensive phylogenetic reconstructions, the genus is frequently recovered as polyphyletic. Notably, certain divergent taxa, such as *B. cinereum*, *B. gelatinosum*, and *B. rimosum*, consistently form separate lineages distinct from the core *Basidiodendron* clade (Fig. 2). This topological instability is partially exacerbated by the inclusion of historical specimens with incomplete molecular data (e.g., *B. cinereum* and *B. rimosum* are represented solely by nrLSU sequences in this clade) (Weiss and Oberwinkler 2001). Despite their phylogenetic divergence and incomplete multilocus data, these three species are conservatively retained within *Basidiodendron* in the present study (Fig. 2). This taxonomic decision is primarily based on their possession of basidia with well-developed involucres, which have historically been regarded as a unique and defining morphological diagnostic characteristic of the genus (Wells and Raitviir 1975; Spirin et al. 2020, 2021). However, these species exhibit cylindrical basidiospores, which conspicuously differ from the globose to subglobose basidiospores characteristic of the core *Basidiodendron* clade (Luck-Allen 1963). Although the presence of involucres justifies their temporary retention within the genus, their differing basidiospore morphology and isolated phylogenetic positions strongly suggest that they may require separate generic treatments in future studies when expanded sampling and complete multigene datasets become available. To date, *Basidiodendron* includes 38 accepted species (MycoBank, accessed August 2026). With the addition of the nine new species delimited in the present study, the total number of species in the genus reaches 47.

Morphologically, most members of *Auriculariales (Basidiomycota)* have small- or medium-sized, colorless (hyaline), smooth basidiospores, whereas three species with ornamented (warted or spiny) basidiospores have been reported in the genus *Basidiodendron* (Spirin et al. 2020). In the present study, the discovery of distinctively spiny spores in the new species *B. album* further enriches this rare morphological spectrum. Furthermore, examination of the distribution of spore ornamentation and involucre-like basidia across the phylogenetic tree (Fig. 2) demonstrates that both traits are homoplastic and have diverged in multiple distantly related species within the genus. This remarkable evolutionary plasticity highlights the limitations of using traditional micromorphological traits alone for species delimitation without robust molecular evidence.

Historically, classification within *Auriculariales* relied heavily on macroscopic traits, such as coriaceous hymenophores and gelatinous basidiomata (Bandoni 1984; Weiss and Oberwinkler 2001). However, consistent with recent molecular findings (Spirin et al. 2025), the multigene phylogeny demonstrates that such macroscopic features are highly homoplastic. A similar phenomenon is observed within the established family *Auriculariaceae*, where the genus *Auricularia* is characterized by conspicuously gelatinous basidiomata, whereas cofamilial genera such as *Adustochaete* and *Heterochaete* possess entirely coriaceous basidiomata (Wu et al. 2014, 2015; Malysheva and Spirin 2017; Alvarenga et al. 2019). The phylogenetic clustering of taxa with distinctly gelatinous basidiomata alongside those with predominantly coriaceous or waxy basidiomata indicates that transitions between gelatinous and non-gelatinous states are evolutionarily labile across *Auriculariales*. These structural variations represent convergent ecological adaptations; specifically, gelatinous basidiomata enable moisture absorption and prevent desiccation, whereas coriaceous basidiomata enhance structural longevity (Halbwachs et al. 2016). By resolving the phylogenetic positions of these morphologically diverse novel species within the core *Basidiodendron* clade, the present study further confirms that suprageneric classification must be based on true evolutionary homologies rather than plastic macroscopic traits.

Notably, nine new species described in this study were found on the Yunnan–Guizhou Plateau in southwestern China. As a globally recognized biodiversity hotspot, this region features complex topography and diverse forest ecosystems, ranging from tropical rainforests to subtropical monsoon forests. These varied habitats provide extensive ecological niches and ideal conditions for the geographic isolation and speciation of wood-inhabiting fungi. In conclusion, through integrative morphological and molecular analyses, the present study describes nine new species in the core *Basidiodendron* clade, significantly expanding the known species diversity of the genus. This rigorous multilocus taxonomic framework provides a robust foundation for future fine-scale generic revisions and ecological research within *Auriculariales*.

## Supplementary Material

XML Treatment for Basidiodendron

XML Treatment for Basidiodendron eyrei

XML Treatment for Basidiodendron album

XML Treatment for Basidiodendron arachnoideum

XML Treatment for Basidiodendron dehongense

XML Treatment for Basidiodendron fragilissimum

XML Treatment for Basidiodendron odontoideum

XML Treatment for Basidiodendron rigidum

XML Treatment for Basidiodendron ruiliense

XML Treatment for Basidiodendron tenissimum

XML Treatment for Basidiodendron zhaotongense

## Additional information

### Artificial Intelligence (AI) use

The authors accept full responsibility for the content of the manuscript, including the disclosure of any use of AI.

No AI tools were used in the preparation of this manuscript.

### Adherence to national and international regulations

All the fungal strains used in this study have been legally obtained, respecting the Convention on Biological Diversity (Rio Convention).

### Author ORCIDs

Q. Yuan https://orcid.org/0000-0002-6732-1656

S. You https://orcid.org/0009-0005-3948-6217

S.N. Wijesinghe https://orcid.org/0000-0002-5625-9578

L. Wang https://orcid.org/0009-0004-6274-5953

X. Liu https://orcid.org/0000-0003-0100-2094

M. Ghobad-Nejhad https://orcid.org/0000-0002-7807-4187

C. Zhao https://orcid.org/0000-0002-8668-1075

### Data availability

All of the data that support the findings of this study are available in the main text.

## References

[B1] Alvarenga RLM, Spirin V, Malysheva V et al. (2019) Two new genera and six other novelties in *Heterochaete**sensu lato* (*Auriculariales*, *Basidiomycota*). Botany 97: 439–451. 10.1139/cjb-2019-0046

[B2] Alvarenga RLM, Westphalen MC, Gibertoni TB (2023) *Elmerina* and *Protomerulius*: novelties from Brazil based in morphological and molecular analyses. Plant Systematics and Evolution 309: 39. 10.1007/s00606-023-01875-x

[B3] Bandoni RJ (1984) The *Tremellales* and *Auriculariales*: An alternative classification. Nippon Kingakkai Kaiho 25: 489–530.

[B4] Bromhead EF (1840) Remarks on the botanical system of Professor Perleb. Mag Nat Hist J Zool Bot Mineral Geol Meteorol 4: 329–338.

[B5] Crous PW, Groenewald JZ, Bensch K et al. (2025) Genera of phytopathogenic fungi known from culture: 1–379. Studies in Mycology 112: 261–633. 10.3114/sim.2025.112.05 PMC12786776 41522876

[B6] Crous PW, Akram W, Albuquerque GMR et al. (2026) Fungal Planet description sheets: 1868–1920. Persoonia 56: 1–173. 10.3114/persoonia.2026.56.01 PMC13409184 42524220

[B7] Cui YY, Fan XP, Guo LJ et al. (2024) New elements of funga from southwestern China: species of *Tremellodendropsis* and *Guepinia*. Mycosystema 43: 230266. 10.13346/j.mycosystema.230266

[B8] Dai YC, Yang ZL, Cui BK et al. (2021) Diversity and systematics of the important macrofungi in Chinese forests. Mycosystema 40: 770–805. 10.13346/j.mycosystema.210036

[B9] Deng YL, Chen M, Zhang SC et al. (2026) Notes, taxonomy, and phylogeny of wood-inhabiting fungi in *Russulales*. Mycosphere 17: e003. 10.48130/mycosphere-0026-0003

[B10] Dissanayake AJ, Bhunjun CS, Maharachchikumbura SS et al. (2020) Applied aspects of methods to infer phylogenetic relationships amongst fungi. Mycosphere 11: 2652–2676. 10.5943/mycosphere/11/1/18

[B11] Dong JH, Li Q, Su JQ et al. (2025a) *Punctochaete murina* gen. et sp. nov. (*Agaricomycetes*, *Basidiomycota*) from southwestern China. European Journal of Taxonomy 981: 96–113. 10.5852/ejt.2025.981.2821

[B12] Dong JH, Xu Y, Jiang QQ et al. (2025b) A new genus and two new species of *Auriculariales (Basidiomycota)* from southwest China, evidenced by morphological characteristics and phylogenetic analyses. Mycological Progress 24: 1–17. 10.21203/rs.3.rs-5169056/v1

[B13] Dong JH, Zhang JL, He SY et al. (2025c) A new corticioid genus of *Auriculariales*—*Nigrochaete*, collected from Yunnan China. Journal of Fungal Research 23: 190–201. 10.13341/j.jfr.2024.1826

[B14] Dong JH, Chen ML, Chen M et al. (2025d) Notes, outline, taxonomy and phylogeny of wood-inhabiting *Agaricales*. Mycosphere 16: 2599–2711. 10.5943/mycosphere/16/1/16

[B15] Duan ZY, Zhao CL (2022) *Basidiodendron yunnanense* (*Auriculariales*), a new species from Southern China based on morphological and molecular evidence. Annales Botanici Fennici 59: 177–183. 10.5735/085.059.0126

[B16] Glez-Peña D, Gómez-Blanco D, Reboiro-Jato M et al. (2010) ALTER: programoriented conversion of DNA and protein alignments. Nucleic Acids Research 38: 14–18. 10.1093/nar/gkq321 PMC2896128 20439312

[B17] Halbwachs H, Simmel J, Bässler C (2016) Tales and mysteries of fungal fruiting: How morphological and physiological traits affect a pileate lifestyle. Fungal Biology Reviews 30: 36–61. 10.1016/j.fbr.2016.04.002

[B18] He MQ, Zhao RL, Hyde KD et al. (2019) Notes, outline and divergence times of *Basidiomycota*. Fungal Diversity 99: 105–367. 10.1007/s13225-019-00435-4

[B19] He MQ, Cao B, Liu F et al. (2024) Phylogenomics, divergence times and notes of orders in *Basidiomycota*. Fungal Diversity 126: 127–406. 10.1007/s13225-024-00535-w

[B20] Hibbett D, Nagy LG, Nilsson RH (2025) Fungal diversity, evolution, and classification. Current Biology 35: R427–R526. 10.1016/j.cub.2025.01.053 40494297

[B21] Kalyaanamoorthy S, Minh BQ, Wong TKF et al. (2017) ModelFinder: Fast model selection for accurate phylogenetic estimates. Nature Methods 14: 587–589. 10.1038/nmeth.4285 PMC5453245 28481363

[B22] Katoh K, Rozewicki J, Yamada KD (2019) MAFFT online service: multiple sequence alignment, interactive sequence choice and visualization. Briefings in Bioinformatics 20: 1160–1166. 10.1093/bib/bbx108 PMC6781576 28968734

[B23] Kirschner R, Yang ZL, Zhao Q et al. (2010) *Ovipoculum album*, a new anamorph with gelatinous cupulate bulbilliferous conidiomata from China and with affinities to the *Auriculariales (Basidiomycota)*. Fungal Diversity 43: 55–65. 10.1007/s13225-010-0038-0

[B24] Kirschner R, Lee IS, Piepenbring M (2012) A new pycnidial fungus with clamped hyphae from Central America. Mycological Progress 11: 561–568. 10.1007/s11557-011-0771-0

[B25] Larsson A (2014) AliView: a fast and lightweight alignment viewer and editor for large data sets. Bioinformatics 30: 3276–3278. 10.1093/bioinformatics/btu531 PMC4221126 25095880

[B26] Larsson KH (2007) Re-thinking the classification of corticioid fungi. Mycological Research 111: 1040–1063. 10.1016/j.mycres.2007.08.001 17981020

[B27] Li Y, Nie T, Nakasone KK et al. (2023) Taxonomy and phylogeny of corticioid fungi in *Auriculariaceae* (*Auriculariales*, *Basidiomycota*): A new genus, five new species and four new combinations. Journal of Fungi 9: 318. 10.3390/jof9030318 PMC10056916 36983486

[B28] Liu SL, Shen ZQ, Li QZ et al. (2022) *Alloexidiopsis* gen. nov., a revision of generic delimitation in *Auriculariales (Basidiomycota)*. Frontiers in Microbiology 13: 1–12. 10.3389/fmicb.2022.894641 PMC9315202 35903469

[B29] Luck-Allen R (1963) The genus *Basidiodendron*. Canadian Journal of Botany 41: 1025–1052. 10.1139/b63-087

[B30] Lutzoni F, Kauff F, Cox CJ et al. (2004) Assembling the fungal tree of life: progress, classification, and evolution of subcellular traits. American Journal of Botany 91: 1446–1480. 10.3732/ajb.91.10.1446 21652303

[B31] Malysheva V, Spirin V (2017) Taxonomy and phylogeny of the *Auriculariales* (*Agaricomycetes*, *Basidiomycota*) with stereoid basidiocarps. Fungal Biology 121: 689–715. 10.1016/j.funbio.2017.05.001 28705397

[B32] Malysheva V, Spirin V, Miettinen O et al. (2018) Revision of *Protohydnum* (*Auriculariales*, *Basidiomycota*). Mycological Progress 17: 805–814. 10.1007/s11557-018-1393-6

[B33] Matheny PB (2005) Improving phylogenetic inference of mushrooms with *RPB*1 and *RPB*2 nucleotide sequences (*Inocybe*, *Agaricales*). Molecular Phylogenetics and Evolution 35: 1–20. 10.1016/j.ympev.2004.11.014 15737578

[B34] Matheny PB, Liu YJ, Ammirati JF et al. (2002) Using *RPB*1 sequences to improve phylogenetic inference among mushrooms (*Inocybe*, *Agaricales*). American Journal of Botany 89: 688–698. 10.3732/ajb.89.4.688 21665669

[B35] Miller MA, Pfeiffer W, Schwartz T et al. (2012) The CIPRES science gateway. In: Proceedings of the 1^st^ Conference of the Extreme Science and Engineering Discovery Environment: Bridging from the Extreme to the Campus and Beyond. USA, 1–39.

[B36] Nagy LG, Vonk PJ, Künzler M et al. (2023) Lessons on fruiting body morphogenesis from genomes and transcriptomes of *Agaricomycetes*. Studies in Mycology 104: 1–85. 10.3114/sim.2022.104.01 PMC10282164 37351542

[B37] Nitare J (2014) *Tremellodendropsis helvetica* (Schild) Nitare comb. et stat. nov. found on the island of Öland, SE Sweden (in Swedish with English abstract). Svensk Mykologisk Tidskrift 35: 24–31.

[B38] Pawlowski J, Audic S, Adl S et al. (2012) CBOL protist working group: barcoding eukaryotic richness beyond the animal, plant, and fungal kingdoms. PLOS Biology 10: e1001419. 10.1371/journal.pbio.1001419 PMC3491025 23139639

[B39] Petersen JH (1996) The Danish mycological societýs colour-chart. Foreningen til Svampekundskabens Fremme Greve, Farvekort, Denmark.

[B40] Rehner S, Samuels GJ (1994) Taxonomy and phylogeny of *Gliocladium* analysed from nuclear large subunit ribosomal DNA sequences. Mycological Research 98: 625–634. 10.1016/S0953-7562(09)80409-7

[B41] Rick SJ (1938) Resupinati Riograndenses. Brotéria 7: 74.

[B42] Roberts P (1998) A revision of the genera *Heterochaetella*, *Myxarium*, *Protodontia*, and *Stypella (Heterobasidiomycetes)*. Mycotaxon 69: 209–248. 10.5962/p.415553

[B43] Ronquist F, Teslenko M, van der Mark et al. (2012) MrBayes 3.2: efficient Bayesian phylogenetic inference and model choice across a large model space. Systematic Biology 61: 539–542. 10.1093/sysbio/sys029 PMC3329765 22357727

[B44] Savchenko A, Zamora JC, Shirouzu T et al. (2021) Revision of *Cerinomyces* (*Dacrymycetes*, *Basidiomycota*) with notes on morphologically and historically related taxa. Studies in Mycology 99: 100117. 10.1016/j.simyco.2021.100117 PMC8645972 34934464

[B45] Sotome K, Maekawa N, Nakagiri A et al. (2014) Taxonomic study of Asian species of poroid *Auriculariales*. Mycological Progress 13: 987–997. 10.1007/s11557-014-0984-0

[B46] Spirin V, Malysheva V, Larsson KH et al. (2018) On some forgotten of *Exidia* and *Myxarium* (*Auriculariales*, *Basidiomycota*). Nordic Journal Botany 36: e01601. 10.1111/njb.01601

[B47] Spirin V, Malysheva V, Roberts P et al. (2019a) A convolute diversity of the *Auriculariales* (*Agaricomycetes*, *Basidiomycota*) with sphaeropedunculate basidia. Nordic Journal of Botany 37: 1–26. 10.1111/njb.02394

[B48] Spirin V, Malysheva V, Miettinen O et al. (2019b) On *Protomerulius* and *Heterochaetella* (*Auriculariales*, *Basidiomycota*). Mycological Progress 18: 1079–1099. 10.1007/s11557-019-01507-0

[B49] Spirin V, Malysheva V, Haelewaters D et al. (2019c) Studies in the *Stypella vermiformis* group (*Auriculariales*, *Basidiomycota*). Antonie van Leeuwenhoek 112: 753–764. 10.1007/s10482-018-01209-9 PMC6456474 30535961

[B50] Spirin V, Malysheva V, Mendes-Alvarenga RL et al. (2020) Studies in *Basidiodendron eyrei* and similar-looking taxa (*Auriculariales*, *Basidiomycota*). Botany 98: 623–638. 10.1139/cjb-2020-0045

[B51] Spirin V, Malysheva V, Schoutteten N et al. (2021) Studies in the *Basidiodendron caesiocinereum* complex (*Auriculariales*, *Basidiomycota*). Mycological Progress 20: 1275–1296. 10.1007/s11557-021-01724-6

[B52] Spirin V, Malysheva V, Viner I et al. (2025) Additions to the taxonomy of the *Auriculariales (Basidiomycota)* with pedunculate basidia. Mycokeys 120: 339–392. 10.3897/mycokeys.120.155492 PMC12381581 40881510

[B53] Talbot PHB (1965) Studies of “*Pellicularia*” and associated genera of *Hymenomycetes*. Persoonia 3: 371–406.

[B54] Tohtirjap A, Hou SX, Rivoire B et al. (2023) Two new species of *Exidia**sensu lato* (*Auriculariales*, *Basidiomycota*) based on morphology and DNA sequences. Frontiers in Microbiology 13: 1080290. 10.3389/fmicb.2022.1080290 PMC9973447 36866163

[B55] Vilgalys R, Hester M (1990) Rapid genetic identification and mapping of enzymatically amplified 294 ribosomal DNA from several *Cryptococcus* species. Journal of Bacteriology 172: 4238–4246. 10.1128/jb.172.8.4238-4246.1990 PMC213247 2376561

[B56] Wakefield EM (1914) Some new British hymenomycetes. Transactions of the British Mycological Society 5: 126–134. 10.1016/S0007-1536(14)80010-7

[B57] Wang L, He SY, Lambert C et al. (2026) Comprehensive morphological and phylogenetic analyses of *Hymenochaetales (Basidiomycota)* unveil one new genus and twenty-six novel wood-inhabiting species from southwestern China. Persoonia 56: 328–380. 10.3114/persoonia.2026.56.05 PMC13409181 42524241

[B58] Wang XW, Liu SL, Zhou LW (2023) An updated taxonomic framework of *Hymenochaetales* (*Agaricomycetes*, *Basidiomycota*). Mycosphere 14: 452–496. 10.5943/mycosphere/14/1/6

[B59] Weiss M, Oberwinkler F (2001) Phylogenetic relationships in *Auriculariales* and related groups – hypotheses derived from nyclear ribosomal DNA sequences. Mycological Research 105: 403–415. 10.1017/S095375620100363X

[B60] Wells K (1994) Jelly fungi, then and now! Mycologia 86: 18–48. 10.1080/00275514.1994.12026372

[B61] Wells K, Raitviir A (1975) The Species of *Bourdotia* and *Basidiodendron (Tremellaceae)* of the U.S.S.R. Mycologia 67: 904–922. 10.1080/00275514.1975.12019824

[B62] White TJ, Bruns T, Lee S et al. (1990) Amplification and direct sequencing of fungal ribosomal RNA genes for phylogenetics. In: Innis MA, Gelfand DH, Sninsky JJ et al. (Eds) PCR protocols: a guide to methods and applications. Academic Press, New York, USA, 315–322. 10.1016/B978-0-12-372180-8.50042-1

[B63] Wu F, Yuan Y, Malysheva VF et al. (2014) Species clarification of the most important and cultivated *Auricularia* mushroom “Heimuer”: evidence from morphological and molecular data. Phytotaxa 186(5): 241–253. 10.11646/phytotaxa.186.5.1

[B64] Wu F, Yuan Y, He SH et al. (2015) Global diversity and taxonomy of the *Auricularia auricula-judae* complex (*Auriculariales*, *Basidiomycota*). Mycological Progress 14: 95. 10.1007/s11557-015-1113-4

[B65] Wu F, Zhao Q, Yang ZL et al. (2020) *Exidia yadongensis*, a new edible species from East Asia. Mycosystema 39: 1203–1214. 10.13346/j.mycosystema.200205

[B66] Wu F, Tohtirjap A, Fan LF et al. (2021) Global diversity and updated phylogeny of *Auricularia* (*Auriculariales*, *Basidiomycota*). Journal of Fungi 7: 933. 10.3390/jof7110933 PMC8625027 34829220

[B67] Yang Y, Xu Y, Wang L et al. (2025) Multigene phylogeny of seven wood-inhabiting fungal orders in *Basidiomycota*, and proposal of a new genus and thirteen new species. Mycosphere 16: 245–295. 10.5943/mycosphere/16/1/4

[B68] Zhou LW, Dai YC (2013) Phylogeny and taxonomy of poroid and lamellate genera in the *Auriculariales (Basidiomycota)*. Mycologia 105: 1219–1230. 10.3852/12-212 23709572

